# Neural correlates of resilience to trauma during adolescence: A multi‐modal study

**DOI:** 10.1002/jcv2.70066

**Published:** 2025-11-02

**Authors:** Lu Zhang, Vanessa L. Cropley, Divyangana Rakesh, Sarah Whittle

**Affiliations:** ^1^ Centre for Youth Mental Health University of Melbourne Parkville Victoria Australia; ^2^ Orygen Youth Health Orygen Parkville Victoria Australia; ^3^ Neuroimaging Department Institute of Psychiatry Psychology & Neuroscience King's College London London UK

**Keywords:** adolescence, brain, mental health, resilience, trauma

## Abstract

**Background:**

Understanding resilience mechanisms is important for advancing early intervention strategies, yet research on the neurobiology of resilience in adolescents is limited. The present study examined the brain structural and resting‐state functional connectivity (rsFC) correlates of resilience to internalizing and externalizing symptoms in a large sample of adolescents.

**Methods:**

We analyzed longitudinal data from 8499 adolescents (baseline mean age 9.92 ± 0.62 years) from the Adolescent Brain Cognitive Development Study. Participants were categorized as resilient, maladaptive, vulnerable, or control group based on reports of traumatic events and internalizing/externalizing symptoms 3 years later. We used multinomial regressions to examine associations of brain structure (gray matter, *N* = 7526; white matter, *N* = 6959) and rsFC (*N* = 6485) with resilience. Sex differences were also investigated.

**Results:**

For internalizing symptoms, increased odds of being resilient (relative to controls) were associated with postcentral gyrus thickness. For externalizing symptoms, increased odds of being resilient was associated with putamen volume (relative to being vulnerable), and rsFC between the dorsal and ventral attention networks, and between cortical networks and hippocampus (relative to controls). Sex‐interaction results implicated gray matter structure, however, robustness of these findings are limited.

**Conclusion:**

Resilience in the context of internalizing symptoms in adolescence may be associated with structural alterations in cortical regions involved sensory processing. Neural correlates of resilience in the context of externalizing symptoms remain unclear as findings may reflect resilience, trauma exposure, or psychopathology. This underscores the need for further research into the neurobiological basis of resilience during adolescence.

## Key Points

**What's known:**

- Existing evidence on the neural correlates of resilience to trauma‐related mental health problems during adolescence is limited by a paucity of research investigating white matter and resting‐state connectivity, as well as a lack of investigation of sex differences.

**What's new:**

- Examining resilience in relation to brain structure and connectivity, we identified potential associations of resilience with gray matter structure and resting‐state functional connectivity between cortical networks and between cortical networks and the hippocampus.

**What's relevant:**

- Our preliminary findings suggest that sensory processing regions may contribute to adolescent resilience in the context of internalizing symptoms. However, sex differences remain largely unknown.

## INTRODUCTION

Childhood adversity, which refers to a broad range of negative childhood experiences including neglect, abuse, poverty, and household dysfunction, is a potent risk factor for later mental health problems (Kessler et al., 2010; McLaughlin et al., 2012; McLaughlin & Lambert, 2017). In particular, childhood trauma, which refers to a subset of adverse experiences that are associated with intense negative emotions such as extreme fear and hopelessness (e.g., sudden loss of a loved one, natural disaster, or near‐death experience; American Psychiatric Association, 2013), is particularly concerning given its high prevalence amongst adolescents. Population‐level studies indicate that as high as two thirds of adolescents report experiencing at least one form of trauma by the age of 16 (Copeland et al., 2007; Saunders & Adams, 2014). Neurobiological alterations have been postulated to underpin the association between childhood trauma and later mental health outcomes (Berens et al., 2017). Indeed, reviews and studies have documented the influence of childhood trauma on brain development (McLaughlin et al., 2019; Teicher et al., 2016; Whittle et al., 2022), and risk for psychopathology (Hanson et al., 2015; McLaughlin & Lambert, 2017). However, while research investigating risk pathways is important (McLaughlin, 2016), this traditional “risk‐focused” approach is limited in its ability to identify factors and mechanisms that may foster resilience. Research on resilience, which is defined as positive adaptions post experiences of threats or challenges to survival (Luthar et al., 2000), is needed. Investigating the neurobiological correlates of resilience during adolescence, a period characterized by marked brain development (Fuhrmann et al., 2015; Rakesh et al., 2024) as well as the onset of several psychiatric disorders (Kessler et al., 2005; Solmi et al., 2022), is pivotal for characterizing the neurobiological factors that may promote resilience.

Neural correlates of resilience typically refer to brain changes associated with positive outcomes or the absence of negative outcomes in the context of stress or adversity (Ioannidis et al., 2020). Common methodologies to examine neural correlates of resilience include testing moderating or mediation pathways of brain markers between adversity and positive outcomes (e.g., Callaghan et al., 2019), or identifying differences in brain markers between individuals that share similar risks but differ in outcomes: resilient individuals, those with high adversity without psychopathology, and “maladaptive” individuals, those with high adversity and psychopathology (e.g., De Bellis et al., 2015; Masten et al., 1999). However, this two‐group approach, although widely used in the literature, fails to account for the possibility that the observed effect may reflect mere absence of psychopathology or general positive functioning regardless of adversity. To interpret differences as true indicators of resilience, others suggest the need to establish: (i) a difference between resilient individuals and controls (or healthy controls or competent individuals as referred to in some past work; absence of adversity and psychopathology) and/or (ii) absence of differences between controls and “vulnerable” (absence of adversity and presence of psychopathology) individuals (Masten et al., 1999; Miller‐Lewis et al., 2013). Despite the limited use of the four‐group approach (potentially due to its stringent criteria for an effect to be interpreted as resilience), given its ability to parse resilience effects from general effects of adversity or psychopathology, the present study leveraged this approach to identify neurobiological correlates specific to resilience.

While several studies have investigated the neural markers of resilience during childhood and adolescence (Eaton et al., 2022; Feder et al., 2019; Masten et al., 2021), our recent systematic review of this literature showed mostly mixed findings (Zhang et al., 2023). Nevertheless, there was some preliminary support that resilience to internalizing/post‐traumatic Stress Disorder (PTSD) symptoms was associated with greater subcortical (i.e., amygdala and hippocampus) volumes and reduced connectivity between prefrontal and subcortical regions. Notably, most consistent findings were from studies that leveraged the group approach. For example, adolescents resilient to PTSD symptoms, compared to those with PTSD symptoms, showed reduced connectivity between inferior frontal gyrus (IFG) and hippocampus in two studies (Li et al., 2021; Sheynin et al., 2020).

On the contrary, limited research has examined the neural correlates of resilience to externalizing symptoms. Prior work from our group suggests that increased resting‐state functional connectivity (rsFC) within the salience network may confer resilience against problematic substance use among those with maltreatment experiences (Rakesh, Allen, et al., 2021). Given prior evidence of distinct neural correlates for internalizing and externalizing symptoms during adolescence (e.g., Whittle et al., 2020), it is plausible that resilience in the context of these symptom domains is similarly associated with distinct neural mechanisms. Therefore, further research is warranted to investigate the neurobiological correlates of resilience in the context of externalizing symptoms.

Additionally, our review noted various limitations in the literature, including a dearth of research examining sex differences in the neural correlates of resilience. Only one study was identified, which observed resilience to be associated with an opposite pattern of low‐frequency fluctuations in the orbitofrontal cortex in males and females (Wang et al., 2019). Given evidence for sex differences in neurodevelopmental trajectories (Lenroot & Giedd, 2010), and in the influence of adversity on the developing brain (Bath, 2020; De Bellis & Keshavan, 2003; Rakesh, Kelly, et al., 2021; Whittle et al., 2017), further research is needed to understand potential sex differences in neural correlates of resilience.

Given the widespread alterations in brain structure, function, and connectivity associated with adversity (McLaughlin et al., 2019; Rakesh & Whittle, 2021), which have been suggested to contribute to mental health outcomes (Rakesh, Kelly, et al., 2021; Whittle et al., 2024), it is possible that resilience may show similarly widespread patterns. However, few studies have adopted whole‐brain approaches, with most research focusing on the structure or function of specific regions (Zhang et al., 2023). Despite evidence linking both adversity and mental health outcomes with rsFC and white matter microstructure (McLaughlin et al., 2019; Whittle et al., 2024), few studies have examined the neurobiology of resilience using resting‐state functional magnetic imaging (rsfMRI) or diffusion tensor imaging (DTI). As such, to better understand the likely complex neural processes associated with resilience, holistic examination of the neural correlates of resilience across different imaging modalities using a whole‐brain approach is needed.

To address these gaps in the literature, the present study aimed to examine the neural correlates of resilience in the context of internalizing and externalizing symptoms among adolescents with childhood trauma (a severe form of adversity) by employing a multimodal approach to examine structure and connectivity across the brain. Using data from the Adolescent Brain Cognitive Development (ABCD) study and the conventional group approach, we examined associations between resilience and (i) cortical thickness, surface area, and subcortical volume, (ii) white matter microstructure as measured by fractional anisotropy (FA), and (iii) within and between network rsFC. We also examined sex differences in these associations.

Based on existing literature, we formulated the following broad predictions: (i) resilience to internalizing symptoms will be associated with greater amygdala and hippocampal volume, as well as reduced connectivity between cortical and subcortical networks (Zhang et al., 2023); (ii) resilience to externalizing symptoms will be associated with greater within‐salience network connectivity (Rakesh, Allen, et al., 2021); and (iii) resilience to internalizing and externalizing symptoms will be associated with greater FA, particularly in tracts involving frontal regions (Galinowski et al., 2015). Given the dearth of studies examining sex differences, no specific hypotheses regarding sex differences were made.

## METHODS

The present study was preregistered on the Open Science Framework (https://osf.io/bauc7). Deviations from the preregistration have been fully described.

### Participants

Participants were sampled from the ongoing ABCD Study (https://abcdstudy.org/; release 5.0). The study recruited ∼11,800 9–10‐year‐old children across 21 study sites in the U.S., intending to comprehensively characterize mental health and cognitive development across adolescence. Details of the study protocol and recruitment processes have been previously documented (Barch et al., 2018; Garavan et al., 2018). Ethics approval was obtained from the Institutional Review Board of each study site. Written informed consent was obtained from parents/caregivers and all participants provided assent. All participants with available data for variables of interest (i.e., trauma and imaging data at baseline, and mental health data at 3‐year follow‐up) were included in the present study, leading to a final sample of 8499 participants.

### Behavioral measures

#### Trauma

The parent‐report Kiddie Schedule for Affective Disorders and Schizophrenia (K‐SADS) PTSD module was used to assess childhood trauma exposure (Kaufman et al., 1997). Trauma was operationalized as a binary variable of exposed versus non‐exposed; adolescents with any traumatic event endorsed on the K‐SADS PTSD module were coded as trauma‐exposed. Binarization of trauma experiences was done due to a heavy right‐skewed distribution (see Supporting Information S1: Appendix S1).

#### Psychopathology

Adolescent internalizing and externalizing symptoms were assessed using the Brief Problem Monitor (BPM) scale, a short youth‐report version of the Child Behavior Checklist (Achenbach, 1999, 2009). Given that psychopathology symptoms typically emerge later in adolescence (Solmi et al., 2022), and that third‐year follow‐up data was the last wave of complete data available, symptom data from this wave was used to assess mental health outcomes.

### Neuroimaging

Neuroimaging data was acquired across sites using harmonized protocols (see Casey et al., 2018). MRI scanners used included 3T Siemens, Phillips, and General Electric with a 32‐channel head coil. Preprocessing of images was performed using a standard pipeline by the ABCD Data Analysis and Informatics Core (see Hagler et al., 2019). Quality control recommendations provided by the ABCD Study were used, and only those that met quality control for the relevant imaging modalities were included in analyses. For further details on imaging acquisition, preprocessing, and quality control see Casey et al. (2018) and Hagler et al. (2019).

Gray matter thickness and surface area of 34 cortical regions and volumes of seven subcortical regions were obtained using the Desikan‐Killiany and ASEG parcellation atlases, respectively, in FreeSurfer version 7.1.1 (Desikan et al., 2006; Fischl et al., 2002). FA of 19 white matter tracts was derived using Atlas Track (Hagler et al., 2009). To allow for comparison with previous work, we examined FA specifically given it is the most commonly investigated metric for white matter microstructure in the literature (Zhang et al., 2023). rsFC (Fisher transformed Pearson correlation) values of 12 predefined networks based on the Gordon parcellation scheme (Gordon et al., 2016) were extracted. This includes 12 within‐network and 66 between‐network connectivity metrics. Connectivity between the 12 networks and the amygdala and hippocampus were also extracted as cortical connectivity with these subcortical regions have been implicated in previous work (Zhang et al., 2023). In total, we examined 196 imaging variables (75 gray matter, 19 white matter, and 102 rsFC variables).

### Statistical analyses

#### Outcome group categorization

As preregistered, we first attempted to classify groups based on trauma exposure at baseline and symptoms at the third‐year follow‐up using Latent Profile Analysis, a data‐driven approach to observe characteristically distinct profiles within a sample (Weller et al., 2020). However, groups were unidentifiable due to model convergence issues. Efforts to transform the data to similar scales and reduce skewness did not mitigate these issues. We then followed our preregistered plan and defined our outcome groups using established symptom cut‐offs provided for the BPM (i.e., high symptoms = T‐score >65, Achenbach et al., 2011) and trauma exposure (exposed vs. non‐exposed). Based on previous work that has also adopted the group approach (e.g., Burt et al., 2016), we classified adolescents into profiles of *resilient* (trauma‐exposed, low symptoms), *maladaptive* (trauma‐exposed, high symptoms), *vulnerable* (trauma non‐exposed, high symptoms), and *controls* (trauma non‐exposed, low symptoms).

However, the utility of distinguishing low versus high symptoms based on a small difference around the clinical threshold (i.e., <65) may not reflect meaningful clinical differences (Dekker et al., 2024). This is particularly relevant given the skewness of mental health symptoms toward low or no symptoms (median T‐score ∼ 52, see Supporting Information S1: Appendix S2 Figures S6 and 7) in the present dataset. As such, instead of using T‐score <65 as the cut‐off for low symptom groups (i.e., resilient and controls) as per the preregistration, we used <52 as the cut‐off for these groups to better capture meaningful variation in mental health. T‐score ≥65 remained as the cut‐off for high symptom groups (i.e., maladaptive and vulnerable). For transparency, results as per pre‐registered approach are separately reported in an online supplemental document accessible at https://osf.io/5fdre/. Groups were created separately for internalizing and externalizing symptoms (see Table 1 for group Ns).

**TABLE 1:** Demographic information.

Characteristic	*N* (%); mean (SD)
*N* (female)	8499 (52%)
Age at baseline (years)	9.92 (0.62)
Age at T3 follow‐up	12.92 (0.64)
Average parental education (years)	15.32 (2.56)
Internalizing groups (*N*); internalizing symptoms (mean)	
Maladaptive	305 (4%); 68.09 (2.61)
Vulnerable	453 (5%); 68.17 (2.82)
Controls	3462 (41%); 50.28 (0.45)
Resilient	1785 (21%); 50.29 (0.46)
N/A	2494 (30%);
Externalizing groups (*N*); externalizing symptoms (mean)	
Maladaptive	134 (2%); 67.57 (1.62)
Vulnerable	144 (2%); 67.31 (1.70)
Controls	4440 (52%); 50.16 (0.37)
Resilient	2283 (27%); 50.19 (0.39)
N/A	1498 (18%);
Race (*N*)	
African American	1081 (13%)
Asian	186 (2%)
Hispanic	1639 (19%)
White	4713 (55%)
Other	880 (10%)
Trauma total	0.49 (0.90)
Binary trauma exposure (*N*)	
Non‐exposed	5498 (65%)
Exposed	3001 (35%)
Internalizing symptoms (T score)	53.89 (5.75)
Externalizing symptoms (T score)	52.0 3 (4.19)

*Note*: Symptom T score has a minimum of 50 which reflects a raw score of 0.

We acknowledge that certain group labels may carry connotations of deficiency; however, our choice of terminology is intended solely to maintain consistency with the existing literature (e.g., Burt et al., 2016; Masten et al., 1999). These terms are used to describe participants' experience of adversity and mental health within this specific research context and are not meant to reflect their inherent abilities or functioning.

#### Main analyses

Multinomial logistic regression models were conducted in R version 4.4.0 (R Core Team, 2024) using the VGAM package version 1.1–8 (Yee, 2010). Given that the VGAM package cannot model random effects, one child per family was randomly selected to account for family structure. In addition, scanner was included as a fixed effect in models. Brain variables were standardized and included as the predictor in separate models and the outcome groups (as a categorical variable) were modeled as the response variable. The resilient group was set as the reference group in all models, allowing comparison between the resilient group and every other group (i.e., maladaptive, vulnerable, and controls). We corrected for multiple comparisons using the false discovery rate (FDR). FDR correction was applied to account for multiple ROIs within each imaging modality (i.e., structural MRI [sMRI], DTI, and rsfMRI) and multiple group comparisons (resilient vs. controls, resilient vs. vulnerable, resilient vs. maladaptive) (*p*FDR < .05; 75 comparisons for gray matter, 19 comparisons for white matter, and 102 comparisons for rsFC; Benjamini & Hochberg, 1995). See Supporting Information S1: Appendix S3 for model equations, Appendix S2 Figure S2–7 for data distributions, and Appendix S4 for manipulation check results pertaining to group classification.

To examine whether the observed significant results truly reflect resilience‐specific effects, additional post‐hoc logistic models were conducted to examine differences between all groups. Six post‐hoc models were conducted for each significant finding, which examined the difference between maladaptive, vulnerable, and control groups (in addition to differences between these groups and the resilient group). No multiple comparison correction was applied for post‐hoc analyses.

Based on suggestions from prior work (Masten et al., 1999; Miller‐Lewis et al., 2013), we interpret an effect as reflecting resilience if: (i) a difference is observed between the resilient group and all other groups (i.e., maladaptive, vulnerable, and controls); or we see (ii) differences in the resilient vs. maladaptive and resilient vs. control group comparisons, or (iii) differences in the resilient versus maladaptive comparison and no differences in the control versus vulnerable comparison.

To examine whether neurobiological correlates of resilience differed by sex, analyses were repeated with an additional interaction term between sex and each brain variable with the same FDR correction applied. However, the robustness of the sex‐interaction models is limited given the small sample size of sex‐stratified groups. Nevertheless, the sex‐interaction results are reported in the results section though we did not discuss the possible implications of these results.

Our preregistration specified the use of within‐sample split‐half replication to test the replicability of our results given the power afforded by the ABCD sample (Rakesh et al., 2022; Rakesh, Zalesky, et al., 2021; Saragosa‐Harris et al., 2022). This was not possible due to the small sample sizes of individual groups (e.g., maladaptive group size varied between 47 and 129 for discovery and replication samples). As such, we primarily interpreted the results from analyses using the full sample. We explain the issue in detail in the Supporting Information S1: Appendix S5 with the split‐half replication findings presented in our online supporting information document for transparency purposes.

#### Covariates

We covaried for age, sex, socioeconomic status (SES), and scanner type in all models. Age, sex, and SES were taken from baseline demographics data. Household SES was operationalized as average parental education attainment for both caregivers. We chose parental education over other SES indicators (e.g., household income) based on prior research showing that it is associated with resilience in young people (Reiss et al., 2019). Data for one caregiver was used when data for both was unavailable. In addition, framewise displacement was covaried for in models that included rsFC variables, and total brain volume was covaried for in models that analyzed subcortical volume and cortical surface area (Mills et al., 2016; Parkes et al., 2018).

#### Sensitivity analyses

Preregistered sensitivity analyses with site and race added as additional covariates were not conducted due to the following reasons. First, prior work has shown that race‐associated brain differences in the ABCD study may be accounted for by childhood adversity, including trauma exposure (Dumornay et al., 2023). Given the deeply confounded nature of race and childhood adversity in the ABCD sample, including race in models makes it challenging to disentangle the effects of resilience from race. Second, studies show a high overlap between site and scanner type in the ABCD study, and covarying for site explained minimal additional variance when scanner type has been accounted for (Rakesh et al., 2022; Taylor et al., 2020). As such, analyses were conducted with scanner type (an important covariate for imaging analyses), but not site, as a covariate. Following prior recommendations to report adjusted and unadjusted models, we conducted analyses without the inclusion of SES as a covariate (Hyatt et al., 2020).

## RESULTS

### Demographic information

8499 unique participants were included in analyses, with sample sizes varying between modalities: gray matter (*n* = 7526), white matter (*n* = 6959), and rsFC (*n* = 6485). See Table 1 for sample demographic information and Figure S8 (Supporting Information S1: Appendix S6) for a flow chart depicting the reasons for exclusion. Notably, there was considerable overlap between the internalizing and externalizing groups, with 87% of those resilient to internalizing symptoms also showing resilience to externalizing symptoms, see Table 2 for distribution of groups by imaging modality.

**TABLE 2:** Distribution of groups by imaging modality.

Groups	sMRI	DTI	rsFC
Internalizing groups			
Maladaptive	171	158	139
Resilient	1402	1317	1210
Vulnerable	264	248	228
Controls	2721	2514	2354
Externalizing groups			
Maladaptive	78	72	61
Resilient	1495	1403	1288
Vulnerable	81	70	64
Controls	2904	2692	2518

Abbreviations: DTI, diffusion tensor imaging; rsFC, resting‐state functional connectivity; sMRI, structural magnetic resonance imaging.

### Main analyses

White matter structure did not significantly predict resilient group membership. For internalizing symptoms, increased postcentral thickness was significantly associated with decreased predicted odds of being resilient relative to controls. The same direction of effect was observed for the resilient vs. vulnerable and resilient vs. maladaptive comparisons, although these did not reach statistical significance. See Table 3 for relevant statistics.

**TABLE 3:** Results for significant main effects.

Brain variables	Group comparison	Odds (95% CI)	*pFDR*
Internalizing findings			
Postcentral thickness	MA: RES	1.14, [0.99 1.31]	0.597
	VU:RES	1.15, [1.02 1.30]	0.217
	**HC:RES**	**1.14, [1.06 1.24]**	**0.022**
Externalizing findings			
Putamen volume	MA: RES	0.96, [0.76 1.21]	0.981
	**VU:RES**	**1.36, [1.09 1.70]**	**0.042**
	HC:RES	1.04, [0.97 1.12]	0.960
DAN—VAN connectivity	MA: RES	0.99, [0.79 1.24]	0.981
	VU:RES	0.97, [0.79 1.20]	0.965
	**HC:RES**	**0.88, [0.83 0.94]**	**0.019**
SMN (M)—Hippocampus connectivity	MA:RES	0.90, [0.73 1.11]	0.772
	VU:RES	1.06, [0.87 1.31]	0.965
	**HC:RES**	**1.13, [1.06 1.20]**	**0.019**

*Note*: Bolded text indicates significant main effects. Results for other group comparisons are shown for reference. Values >1 indicate increased odds and <1 indicate decreased odds of being maladaptive/vulnerable/control versus resilient, with increases in brain structure/connectivity.

Abbreviations: CI, confidence interval; Con, control; DAN, dorsal attention network; HC, healthy control; MA, maladaptive; *p*FDR, false‐discovery rate corrected *p‐*values; RES , resilient; sig, significant; SMN [M], sensorimotor mouth network; VAN, ventral attention network; VU, vulnerable.

For externalizing symptoms, increased putamen volume was significantly associated with decreased predicted odds of being resilient relative to being vulnerable. Although not statistically significant, the same trend of effect was observed for the resilient vs. control comparison. rsFC findings were also observed for externalizing symptoms, specifically, increased predicted odds of being resilient relative to controls were associated with (i) increased connectivity between the dorsal attention network (DAN) and ventral attention network (VAN), and (ii) decreased connectivity between the sensorimotor mouth network (SMN [M]) and hippocampus (see Table 3).

**FIGURE 1:**
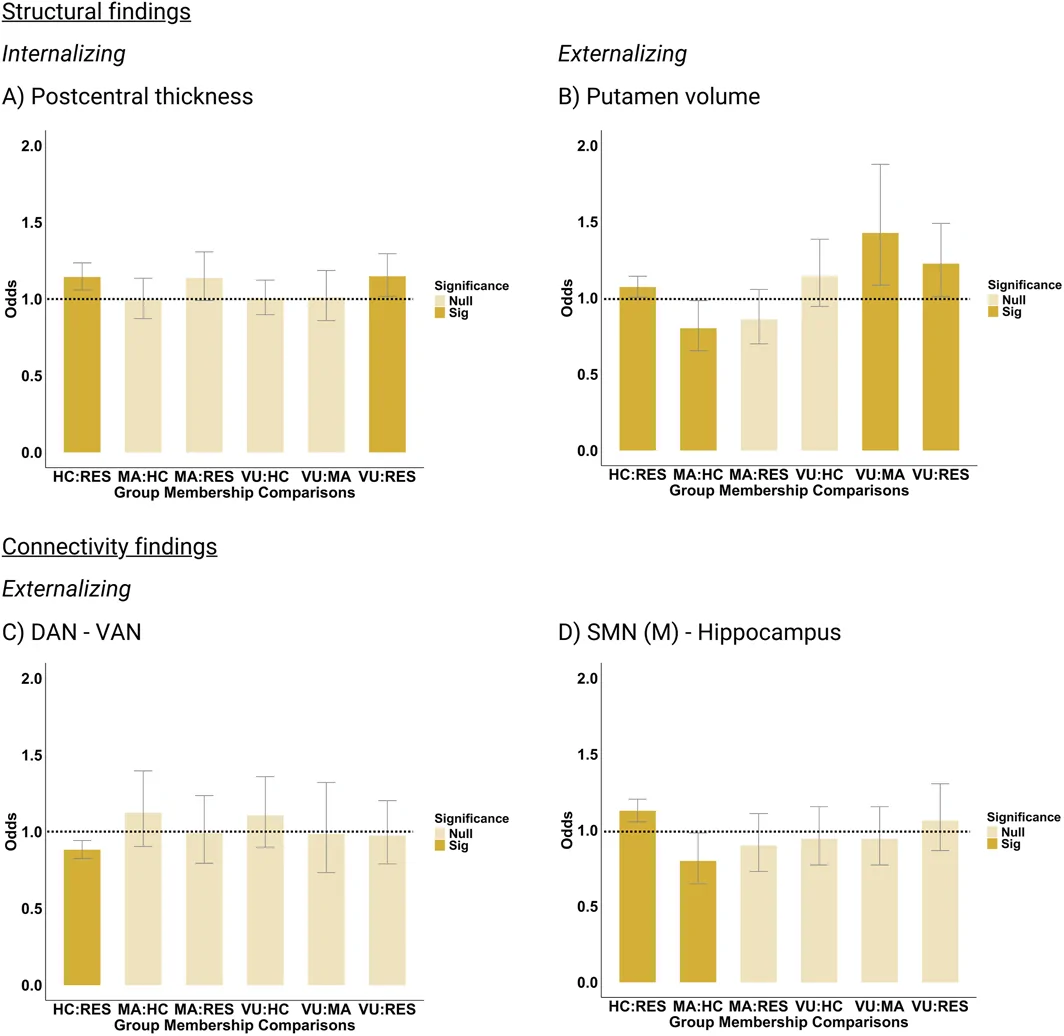
Plots for post‐hoc results of significant main effects. Bars depict predicted odds of group membership for the relevant structural/connectivity finding. Dotted line reflects where odds = 1. Odds values > 1 indicate increased odds and <1 indicate decreased odds of being in the group left of the colon relative to the group right of the colon, with increases in brain structure/connectivity. For example, the leftmost bar in panel A depicts that increased postcentral thickness is associated with increased odds of being healthy relative to being resilient to internalizing symptoms. Significance is determined false discovery rate corrected *p* < 0.05. Error bars indicate 95% confidence intervals. DAN, dorsal attention network; HC, healthy control; MA, maladaptive; RES, resilient; sig, significant; SMN [M], sensorimotor mouth network; VAN, ventral attention network; VU, vulnerable.

Post‐hoc model results (uncorrected) showed few additional group differences (i.e., controls vs. maladaptive, controls vs. vulnerable, maladaptive vs. vulnerable) for putamen volume and SMN (M)—hippocampus connectivity in association with externalizing symptoms (see Figure 1). Specifically, increased putamen volume was additionally associated with decreased predicted odds of being maladaptive relative to controls and being vulnerable. The trending effects of increased putamen volume in association with decreased odds of being resilient relative to being controls or vulnerable reached statistical significance at an uncorrected level. As for SMN (M)—hippocampus connectivity, increased connectivity was further associated with decreased odds of being maladaptive relative to controls. Of note, in these uncorrected analyses, increased postcentral thickness was significantly associated with decreased predicted odds of being resilient relative to being vulnerable to internalizing symptoms.

Sex moderated the association between several brain variables and resilient group membership for internalizing but not externalizing symptoms (see Table 4). Further, we observed significant interaction effects only between sex and gray matter structure. Post‐hoc models were run in males and females separately to interpret the sex difference (see Table 4). Notably, the direction of the effect was opposite in males and females for most results. For example, increased fusiform surface area was associated with lower odds of being resilient relative to being maladaptive in males, but higher odds in females. However, as previously discussed, caution needs to be exercised when considering these results given the small sample size of female and male subsamples.

**TABLE 4:** Results for post‐hoc internalizing group membership comparisons for the significant sex interaction effects separately in males and females.

Brain variables	Group comparison	Males		Females	
		Odds (95% CI)	*p*	Odds (95% CI)	*p*
Cortical surface area					
Banks of superior Temporal sulcus	MA: RES	1.11 [0.88, 1.39]	0.375	0.86 [0.66, 1.12]	0.261
	VU:RES	0.88 [0.72, 1.07]	0.189	0.83 [0.66, 1.05]	0.127
	HC:RES	0.93 [0.82, 1.05]	0.224	**0.86 [0.75, 1.00]**	**0.045**
Frontal pole	MA: RES	1.03 [0.81, 1.31]	0.819	0.86 [0.65, 1.13]	0.284
	VU:RES	0.94 [0.77, 1.16]	0.582	0.88 [0.69, 1.12]	0.305
	HC:RES	0.92 [0.81, 1.05]	0.242	0.90 [0.77, 1.05]	0.174
Fusiform area	MA: RES	1.32 [1.00, 1.75]	0.051	0.84 [0.61, 1.14]	0.263
	VU:RES	1.11 [0.87, 1.41]	0.397	0.89 [0.68, 1.17]	0.421
	HC:RES	1.07 [0.92, 1.25]	0.403	0.88 [0.74, 1.04]	0.131
Inferior parietal	MA: RES	1.08 [0.84, 1.39]	0.551	0.85 [0.64, 1.12]	0.249
	VU:RES	1.02 [0.82, 1.26]	0.871	0.94 [0.74, 1.19]	0.601
	HC:RES	1.07 [0.93, 1.23]	0.323	0.87 [0.75, 1.02]	0.080
Medial orbitofrontal	MA: RES	1.13 [0.85, 1.51]	0.403	0.96 [0.69, 1.32]	0.792
	VU:RES	0.85 [0.67, 1.09]	0.205	0.90 [0.68, 1.19]	0.472
	HC:RES	1.02 [0.87, 1.2]	0.792	0.93 [0.78, 1.11]	0.412
Paracentral	MA: RES	0.94 [0.75, 1.18]	0.579	**0.74 [0.56, 0.97]**	**0.030**
	VU:RES	0.96 [0.79, 1.17]	0.704	0.92 [0.73, 1.16]	0.471
	HC:RES	1.00 [0.88, 1.13]	0.960	0.93 [0.80, 1.08]	0.329
Pars orbitalis	MA: RES	1.17 [0.91, 1.51]	0.227	0.95 [0.71, 1.26]	0.701
	VU:RES	1.19 [0.96, 1.49]	0.111	1.17 [0.92, 1.49]	0.211
	HC:RES	1.02 [0.88, 1.17]	0.820	1.12 [0.96, 1.31]	0.134
Precentral	MA: RES	1.02 [0.78, 1.34]	0.876	0.85 [0.62, 1.16]	0.294
	VU:RES	0.95 [0.75, 1.20]	0.664	1.01 [0.77, 1.33]	0.917
	HC:RES	1.01 [0.87, 1.18]	0.847	1.13 [0.96, 1.34]	0.145
Posterior cingulate	MA: RES	0.97 [0.76, 1.24]	0.799	0.83 [0.62, 1.10]	0.197
	VU:RES	0.96 [0.78, 1.18]	0.689	0.95 [0.74, 1.21]	0.665
	HC:RES	1.11 [0.97, 1.27]	0.139	0.92 [0.79, 1.08]	0.300
Rostral anterior cingulate	MA: RES	1.28 [0.99, 1.66]	0.060	1.07 [0.81, 1.41]	0.647
	VU:RES	0.92 [0.73, 1.15]	0.449	1.06 [0.83, 1.36]	0.620
	HC:RES	1.10 [0.96, 1.27]	0.181	1.03 [0.88, 1.20]	0.729
Supramarginal	MA: RES	0.89 [0.70, 1.13]	0.341	**0.74 [0.54, 1.00]**	**0.046**
	VU:RES	0.92 [0.75, 1.13]	0.454	0.97 [0.75, 1.26]	0.845
	HC:RES	**0.86 [0.76, 0.99]**	**0.030**	0.93 [0.79, 1.09]	0.378
Superior frontal	MA: RES	0.80 [0.60, 1.08]	0.153	0.72 [0.52, 1.01]	0.058
	VU:RES	0.89 [0.69, 1.15]	0.370	**0.66 [0.49, 0.88]**	**0.005**
	HC:RES	0.96 [0.82, 1.12]	0.587	0.84 [0.70, 1.00]	0.053
Subcortical volume					
Accumbens	MA: RES	1.24 [0.98, 1.57]	0.076	1.00 [0.78, 1.29]	0.987
	VU:RES	1.01 [0.83, 1.24]	0.889	0.87 [0.70, 1.08]	0.212
	HC:RES	1.12 [0.98, 1.27]	0.091	1.04 [0.91, 1.19]	0.566
Amygdala	MA: RES	1.11 [0.87, 1.43]	0.397	1.19 [0.91, 1.55]	0.213
	VU:RES	**1.41 [1.14, 1.74]**	**0.001**	0.84 [0.66, 1.08]	0.179
	HC:RES	1.12 [0.97, 1.28]	0.112	0.9 [0.77, 1.04]	0.156
Hippocampus	MA: RES	0.97 [0.75, 1.25]	0.805	0.9 [0.68, 1.19]	0.459
	VU:RES	1.22 [0.99, 1.51]	0.066	0.96 [0.76, 1.22]	0.763
	HC:RES	**1.18 [1.03, 1.35]**	**0.020**	0.88 [0.76, 1.02]	0.098

*Note*: All results pertain to internalizing group comparisons. Bolded text indicates significant results within subsamples of males and females (uncorrected *p* <. 05). Values >1 indicate increased odds and <1 indicate decreased odds of being maladaptive/vulnerable/control versus resilient to internalizing symptoms group, with increases in brain structure/connectivity.

Abbreviations: CI, confidence interval; Con, control; MA, maladaptive; RES, resilient; VU, vulnerable.

For detailed results of sex interaction and non‐significant findings, see Supporting Information S1: Appendices S7–S9 Tables S1–13.

### Sensitivity analyses

Significant results from the main analysis remained stable without SES as a covariate. Moreover, we observed additional statistically significant findings for the prediction of resilient group membership (for both internalizing and externalizing groups) when SES was not included (see online supporting information file). Note that when main and sensitivity analyses were conducted across discovery and replication samples, no findings were replicated, potentially due to the small sample size in some outcome groups as discussed.

## DISCUSSION

This preregistered study leveraged a large sample and multimodal neuroimaging to examine the neural correlates of resilience to trauma‐related internalizing and externalizing symptoms among adolescents. Additionally, we explored potential sex differences in these associations. Hypotheses were partially supported. We found resilience to be associated with gray mater structure and rsFC but not white matter structure. Specifically, for internalizing symptoms, we found higher predicted odds of being resilient (relative to controls) to be associated with decreased postcentral gyrus thickness. For externalizing symptoms, higher predicted odds of being resilient (relative to being vulnerable) were associated with decreased putamen volume, and that higher predicted odds of being resilient (relative to controls) were associated with increased DAN—VAN connectivity and decreased SMN (M)—hippocampus connectivity. However, post‐hoc analyses suggest that these associations may not reflect resilience‐specific markers given these results do not satisfy the conditions required to interpret a finding as a resilience‐specific effect. That is, either (i) a difference is observed between the resilient group and all other groups (i.e., maladaptive, vulnerable, and controls); or (ii) differences in the resilient vs. maladaptive *and* resilient vs. control group comparisons, or (iii) differences in the resilient versus maladaptive comparison and no differences in the control versus vulnerable comparison. Given the preliminary nature of the results, we emphasize the need for caution when considering our interpretations.

Contrary to hypotheses, we did not find evidence for associations of resilience with amygdala and hippocampal volume, nor with white matter microstructure. Of note, previous studies reporting associations between resilience and larger amygdala and hippocampal volume (Li et al., 2021; Morey et al., 2016; Ross et al., 2021) examined resilience specifically to PTSD, which may explain our discrepant findings and suggest that increased amygdala and hippocampal volume is a marker for resilience to PTSD symptoms specifically. Evidence on white matter structure correlates of resilience remain limited with mostly mixed findings (see review Zhang et al., 2023). For example, prior work has similarly reported null results for white matter structure in adolescents (Kim et al., 2019). The mixed findings and limited research on white matter structural correlates of resilience underscore the need for studies to further investigate resilience in association with brain structure.

Despite the predominantly null structural findings, we found that for internalizing symptoms, increased odds of being resilient (relative to controls) were associated with decreased postcentral gyrus thickness. A similar trend (i.e., increased resilience being associated with decreased thickness), though not statistically significant, (see Figure 1), was observed in the comparison between the resilient group and maladaptive and vulnerable groups. It is possible that the lack of statistical significance may be due to the small sample of maladaptive (*N* = 171) and vulnerable (*N* = 264) groups in the present study. This finding suggests that reduced postcentral gyrus thickness may play a role in resilience during adolescence, specifically in the context of internalizing symptoms. This region, also known as the primary somatosensory cortex, is primarily involved in sensory processing and is suggested to play a key role in emotion detection (Kragel & LaBar, 2016). Prior work has demonstrated stress‐related decreases in postcentral gyrus thickness in adolescents (Bartlett et al., 2019). Given this, and the normative decrease in thickness of this region during adolescence (Tamnes et al., 2017), reduced postcentral thickness may represent a trauma adaptation resulting in more efficient emotion recognition process that prevent the development of internalizing symptoms. More broadly, this finding highlights the need to extend research beyond the traditionally emphasized frontal and limbic regions to better understand the neural circuitry underlying resilience.

Findings related to externalizing symptoms showed a less clear pattern. For example, we found that increased odds of being resilient (relative to being vulnerable) were associated with decreased putamen volume demonstrating putamen volume as a possible neural correlate of resilience. Notably, post‐hoc analyses revealed no differences between trauma exposed groups (i.e., maladaptive and resilient) and between non‐exposed groups (i.e., vulnerable and controls), while additional differences were observed between exposed and non‐exposed groups (e.g., vulnerable vs. maladaptive groups and control vs. resilient groups), which may suggest a possible effect of trauma exposure on putamen volume as opposed to resilience. This finding may be in alignment with prior work that showed associations between childhood adversity exposure and structural alterations in the putamen (Bick & Nelson, 2016). Future studies should compare trauma‐exposed and non‐exposed groups to further clarify this relationship. In the present work, such comparisons were not feasible due to highly unequal group sizes (i.e., small maladaptive and vulnerable groups). Specifically, collapsing participants into trauma‐exposed versus non‐exposed groups would have resulted in findings largely driven by data from controls and resilient individuals, hence introducing potential bias.

As for the connectivity findings for externalizing symptoms, we observed mostly differences in the predicted odds of being resilient versus being controls. The lack of significant findings required for interpreting an effect as resilience‐specific and clear trends in post‐hoc analyses results make it unclear if these connectivity results reflect a resilience‐specific effect or a more general effect of adversity exposure or mental health symptoms (or lack thereof). As such, no further interpretations were made to avoid overspeculation. It should be noted that our inability to detect differences between the resilient and maladaptive groups (a key requirement for interpreting an effect as resilience‐specific) may be due to the relatively small sample size of the maladaptive group (i.e., 94–116 individuals for externalizing outcomes and 228–258 for internalizing outcomes) in the present sample. Future research should aim to recruit larger maladaptive groups to investigate these associations further.

The present study also investigated sex differences in the neural correlates of resilience, with the results revealing possible differences in males and females. However, as previously mentioned, the robustness of these findings is limited by the small sample sizes of the sex‐stratified maladaptive and vulnerable groups. To avoid making unwarranted speculations, we refrain from drawing further conclusions. Nonetheless, given the scarcity of research on sex differences in resilience, our preliminary results underscore the important need for future work to consider the role of sex when investigating the neurobiology of resilience during adolescence.

Although we observed additional neural features that may be associated with resilience in sensitivity analyses not adjusting for SES, we do not interpret these findings for the following reasons. SES is a confounder (Wysocki et al., 2022) as it influences both resilience (Qiu et al., 2021; Reiss et al., 2019) and brain structure and function (Buthmann et al., 2024; Michael et al., 2023; Rakesh et al., 2022; Rakesh & Whittle, 2021; Rakesh, Zalesky, et al., 2021; Taylor et al., 2020). Models that account for SES as a confounder can therefore provide more accurate estimates of the association of brain structure and connectivity with resilience.

### Limitations and future directions

While this study has several strengths, including a large sample size and multimodal neuroimaging, several limitations should be noted. First, we used a group‐based approach to operationalize resilience. Although common in the literature, and allows for parsing of resilience from trauma/psychopathology effects, this method does not capture individual differences, or the dynamic, multifaceted nature of resilience as conceptualized in recent frameworks (Kalisch et al., 2021; Masten et al., 2021; Miller‐Lewis et al., 2013). Future studies should consider analytical approaches that better reflect the complexity of resilience across individuals and over time.

Second, we narrowly focused on resilience in the context of mental health outcomes and did not assess positive outcomes such as wellbeing or flourishing. This overlooks emerging perspectives that resilience is not just the absence of symptoms but also the presence of positive adaptations (Masten et al., 2021).

Relatedly, we were unable to directly examine neurobiological adaptations or compensatory processes underlying resilience, as we lacked data prior to trauma exposure. This also limits our ability to determine the directionality of the relationship between the brain and mental health symptoms. Although we assessed baseline brain structure and function, it is unclear if these neural features were a result of differences in mental health at baseline or even prior, or vice versa, or that bidirectional relationships exist. Recent work in developmental neuroscience (DeJoseph et al., 2024) has emphasized the need to adopt “strength‐based” approaches to identify adaptive neural responses to adversity. Longitudinal investigation of neurobiological changes prior and post trauma exposure is therefore needed to explore this further.

Third, we did not examine other outcome domains, including social and cognitive functioning. It remains unclear whether resilience reflects a general capacity across domains or distinct processes specific to each domain. Divergent findings in the neural correlates of resilience may reflect this distinction, with some studies examining cross‐domain resilience (e.g., Burt et al., 2016) and others focusing on domain‐specific resilience (e.g., studies reviewed in Zhang et al., 2023 and the present study). Future work should further explore the domain specificity of resilience to better understand how neurobiological processes may differentially support adaptations across various domains of functioning.

Fourth, our use of dichotomous trauma and symptoms variables undermined our ability to examine nuanced relationships between adversity, brain, and mental health outcomes. For example, it limited our ability to examine the association of type, intensity, and number of traumatic experiences with resilience, which have been suggested as important factors to consider for adversity research (see McLaughlin et al., 2019). Further, the low incidence of several of the trauma types (see Supporting Information S1: Figure S1) and clinically high symptoms in the ABCD sample reduced sample sizes in key groups. This constrained our ability to ascertain whether the observed effects truly reflect resilience. Future research may benefit from more targeted recruitment of “maladaptive” and “vulnerable” individuals.

Fifth, as we focused on childhood trauma prior to baseline and symptoms at third‐year follow‐up, we could not account for pre‐existing mental health or stressors that occurred in between time points. Therefore, we cannot be certain whether the mental health problems observed in some of the trauma‐exposed participants resulted from the trauma or were pre‐existing. Further, some individuals classified as resilient may have experienced fewer subsequent traumatic events compared to peers classified as maladaptive, who may have experienced traumatic events in the interim period, which may have contributed to the observed differences in symptoms. This may have resulted in some individuals being misclassified. Future research should aim to capture and account for pre‐trauma mental health as well as traumatic experiences in between assessments.

Finally, we examined brain features independently, which overlooks potential interactions and interdependencies among different brain features. Advance multivariate approaches may elucidate how complex brain patterns support resilience.

### Conclusions

In sum, the present study comprehensively examined the neural correlates of resilience to both internalizing and externalizing symptoms, across ∼200 brain features and three imaging modalities. We found preliminary support for postcentral thickness being implicated in resilience in the context of internalizing symptoms, and putamen volume, VAN—DAN connectivity, and SMN (M)—hippocampus connectivity being implicated in resilience in the context of externalizing symptoms. However, given the lack of differences between the resilient and maladaptive groups, it is unclear whether the neural findings truly reflect resilience versus markers of trauma exposure or psychopathology. We also observed preliminary sex differences in possible structural and rsFC neural markers of resilience, however, the reliability of these results is limited. Future work is still needed to uncover the relationship between neural properties and resilience during adolescence, as well as related sex differences.

## AUTHOR CONTRIBUTIONS

**Lu Zhang**: Conceptualization; data curation; formal analysis; methodology; visualization; writing—original draft; writing—review and editing. **Vanessa L. Cropley**: Supervision; writing—review and editing. **Divyangana Rakesh**: Conceptualization; methodology; supervision; writing—original draft; writing—review and editing. **Sarah Whittle**: Conceptualization; methodology; supervision; writing—original draft; writing—review and editing.

## CONFLICT OF INTEREST STATEMENT

The authors declare no conflicts of interest.

## ETHICAL CONSIDERATIONS

Ethics approval was obtained from the Institutional Review Board of each the ABCD study site, with central review conducted by the University of California, San Diego Institutional Review Board (IRB #160091, approved September 13, 2016). Written informed consent was obtained from parents/caregivers and all child participants provided assent.

## Supporting information

Supporting Information S1

## Data Availability

The data that support the findings of this study are available from the ABCD Study. Restrictions apply to the availability of these data, which were used under license for this study. Data are available for request from the National Institute of Mental Health Data Archive at https://nda.nih.gov/abcd/request‐access with the permission of the ABCD Consortium.
