# Supplementary material for: Neural correlates of resilience to trauma during adolescence: A multi‐modal study
Source: JCPP Adv. 2025 Nov 2:e70066. Online ahead of print. doi: 10.1002/jcv2.70066 (PMC13339669; doi:10.1002/jcv2.70066)
Supplement: Supplementary file 1 — Supporting Information S1 [file JCV2-9999-e70066-s001.docx]

**Neural Correlates of Resilience to Trauma During Adolescence: A Multi-Modal Study**

**Supporting Information**

*Please note that this document includes only results directly relevant to the main manuscript, additional results that were part of the research but not essential to the manuscript (e.g., split-half replication results) are available in our online supplemental document available in our OSF project folder (https://osf.io/5fdre/).*

# Appendix S1. Types of Traumatic Events Endorsed by Adolescents

**Figure S1.**

*Frequency plot of type of traumatic events endorsed by participants in the sample*


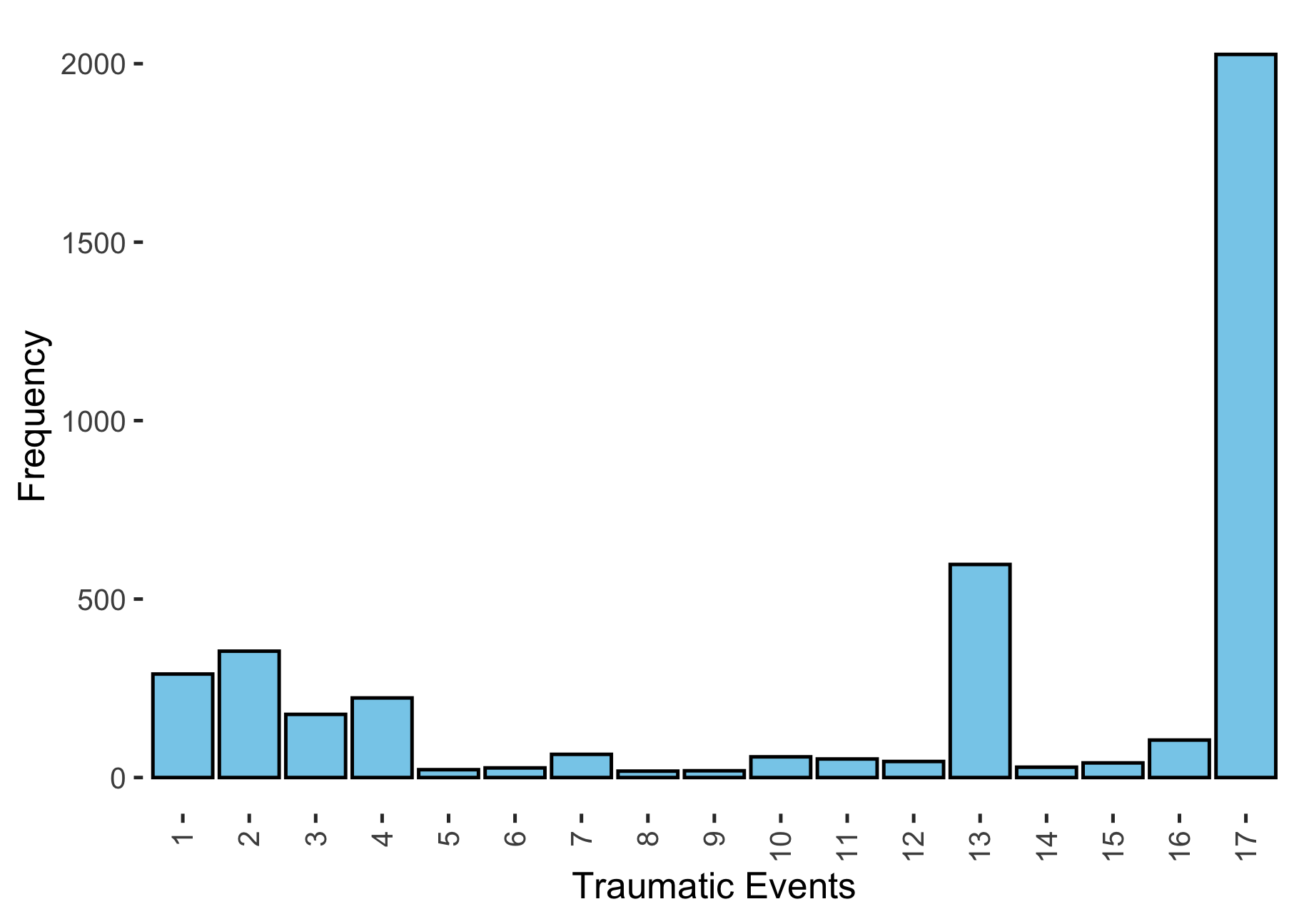


*Note.* 1 = Car accident, 2 = other accident, 3 = fire incident, 4 = other natural disaster, 5 = terror attack, 6 = war, 7 = witnessed significant injury of another person, 8 = physical attack by non-family member, 9 = physical attack by family member, 10 = physical abuse by family member, 11 = death threat from non-family member, 12 = death threat from family member, 13 = physical conflicts between family members, 14 = sexual abuse by family member, 15 = sexual abuse by non-family member, 16 = sexual abuse by peer, 17 = death of someone close.

# Appendix S2. Distribution of Variables

## Figure S2.

## Total Trauma Variable

**
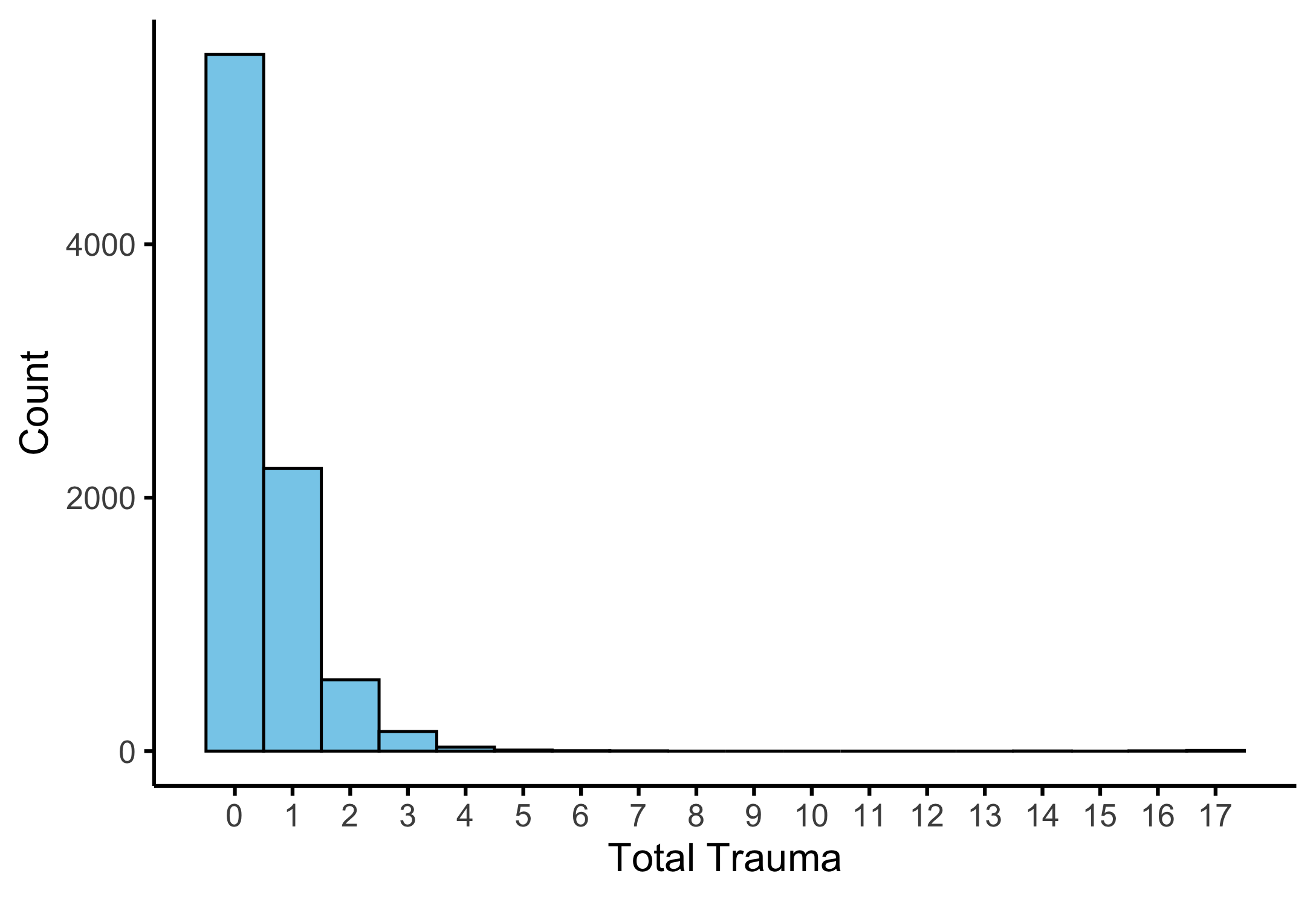
**

- 1. **Brain Variables**

### Figure S3. White Matter Variables


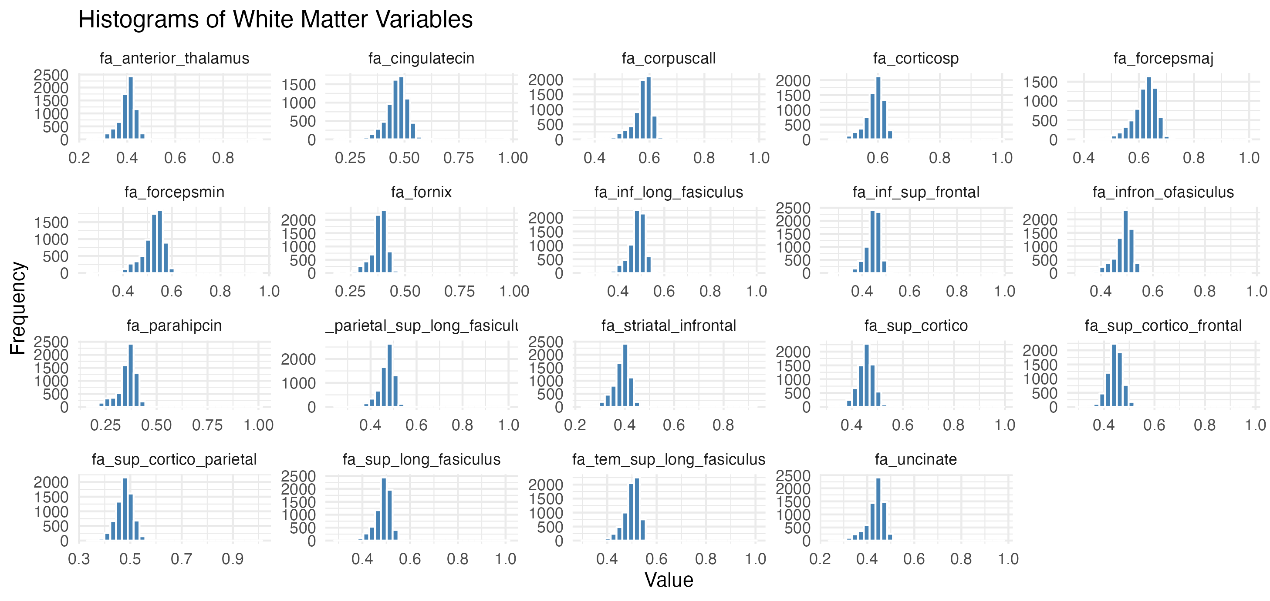


### Figure S4. Gray Matter Variables


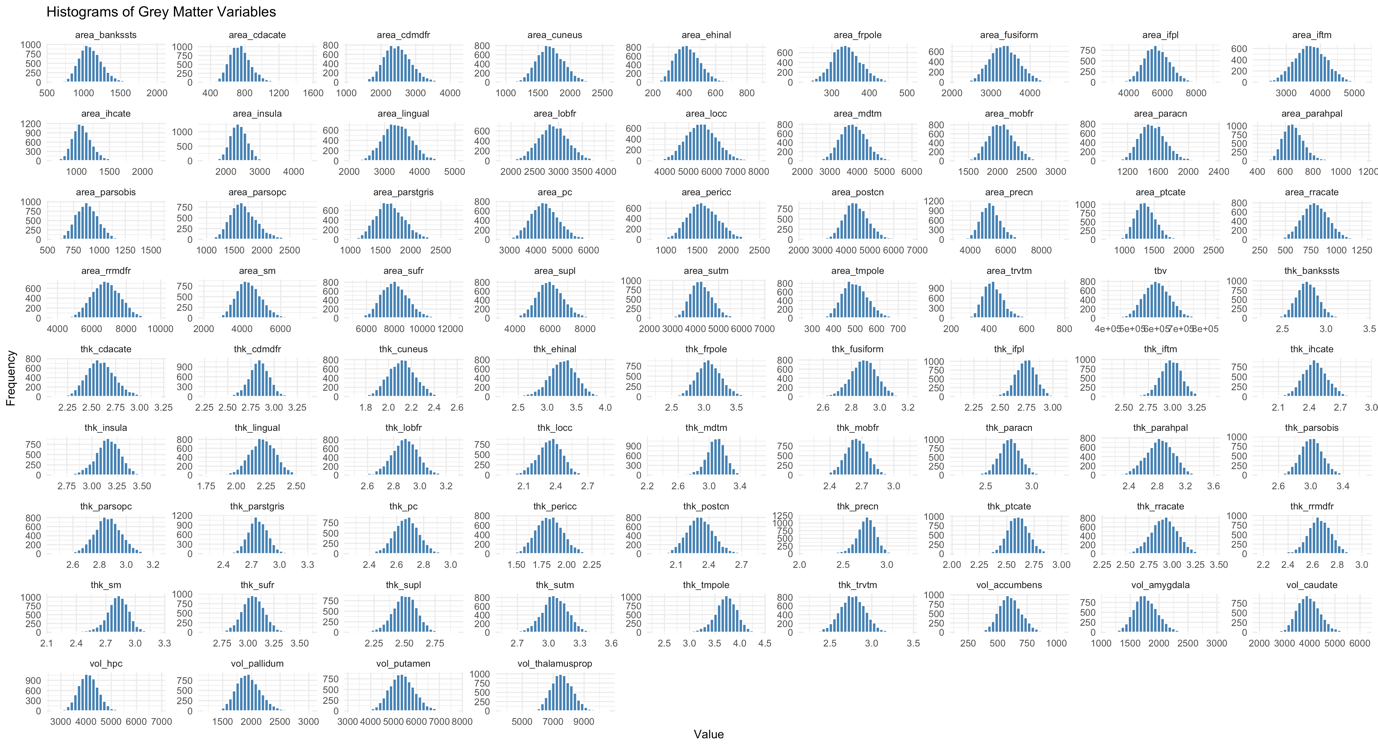


### Figure S5. Resting-state Functional Connectivity Variables


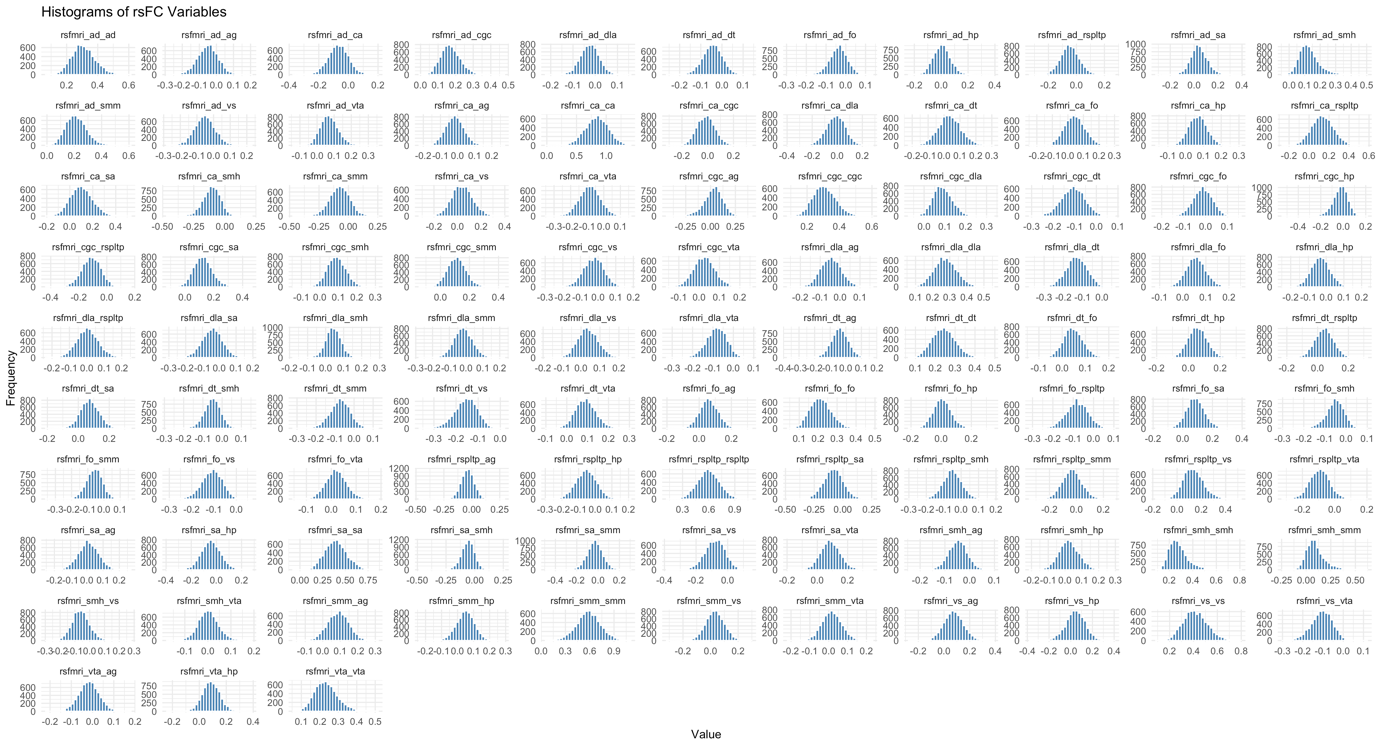


## Mental Health Symptom Variables

### Figure S6. Internalizing Symptoms by Group

**
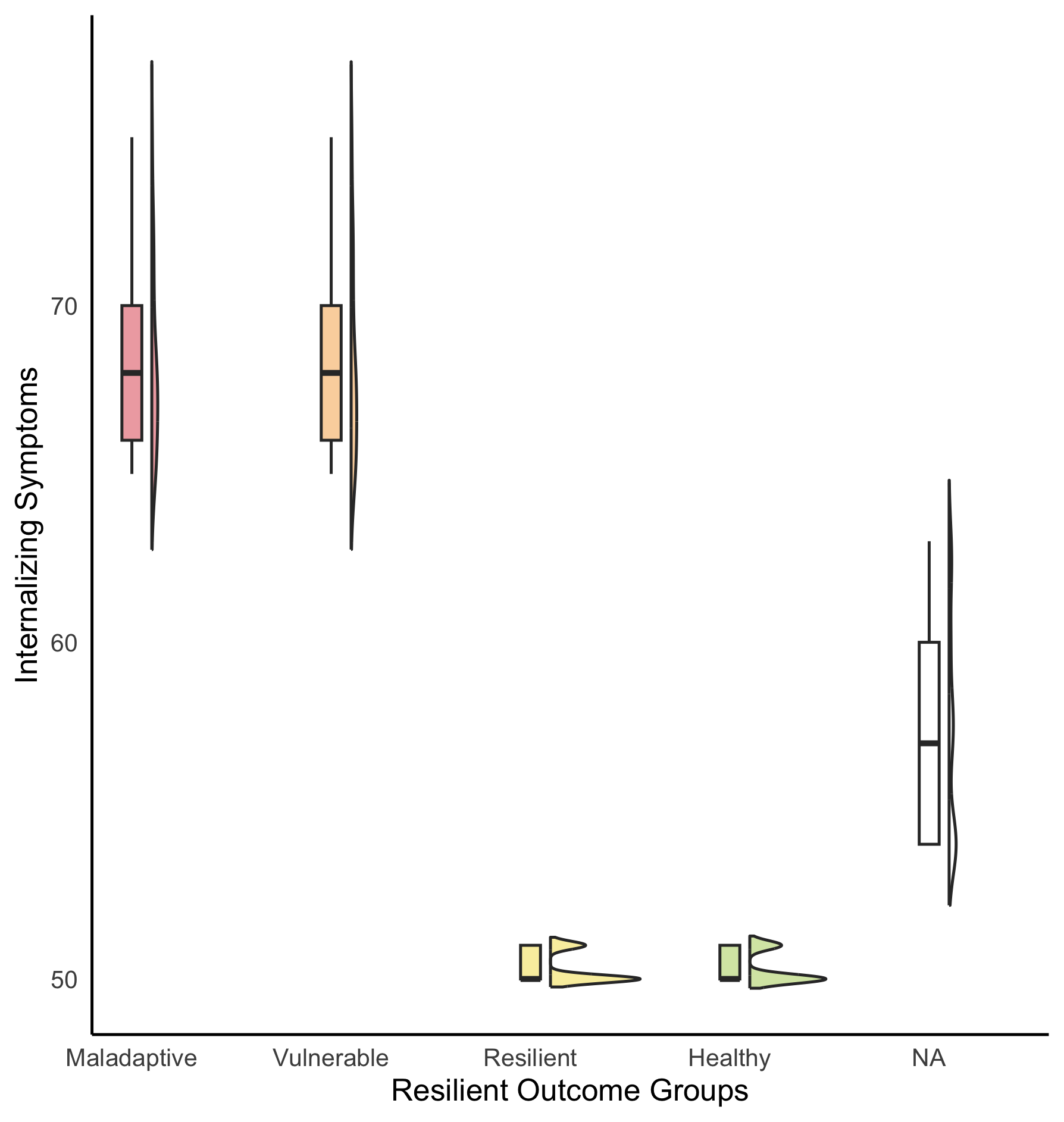
**

### Figure S7. Externalizing Symptoms by Group

**
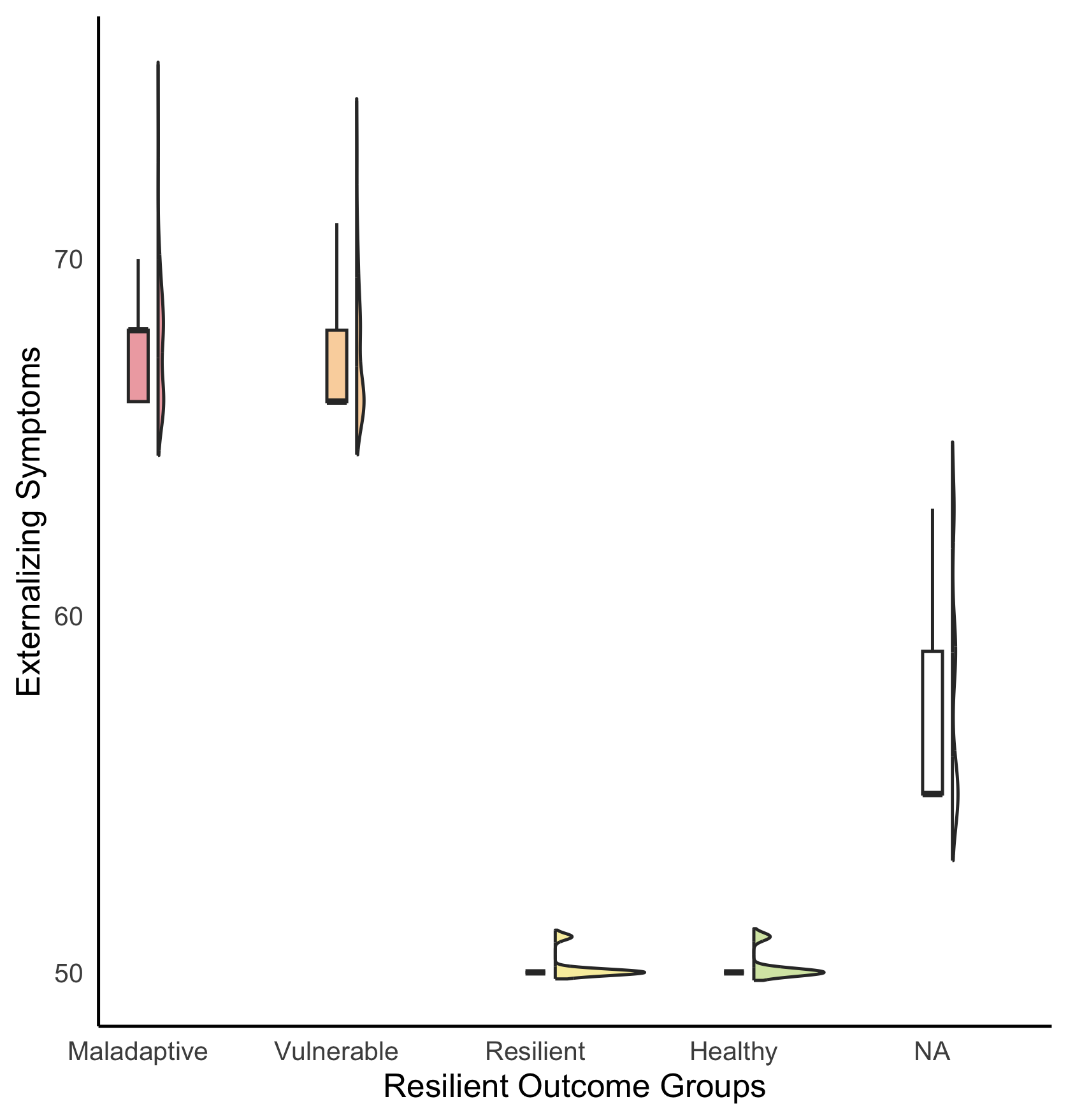
**

# Appendix S3. Model Equations:

## Aim 1 – 3

*Outcome group ~ Brain measure + amygdalae + sex + scanner type + SES^ + FD/TBV^^*

Note. ^ Indicates covariates that were examined in sensitivity analyses. ^^ Indicates covariates that were accounted for only in models examining specific imamygdalaing modality variables.

## Aim 4

*Outcome group ~ Brain measure * sex + amygdalae + scanner type + SES^ + FD/TBV^^*

Note. Lower-order main effects were also included in models. ^ Indicates covariates that were examined in sensitivity analyses. ^^ Indicates covariates that were accounted for only in models examining specific imaging modality variables.

# Appendix S4. Manipulation Checks

ANOVA results showed significant group differences in internalizing (*F*[3, 6001] = 62564, *p* < .001) and externalizing (*F*[3, 6997] = 108810, *p* < .001) symptoms, as well as differences in brain variables across all modalities. Group differences in brain variables was further supported by multinomial logistic regression results showing brain variables across all three modalities predicted maladaptive versus healthy group membership. Together these results indicate support for validation of group categorization.

# Ap.1 dix ons ipendix S5. Split-Half Replication Methods

Preregistered split-half replication method involved the random division of the data into two halves, discovery and replication sets. Analyses were then conducted in both datasets. Only findings that survived FDR correction in both datasets were considered ‘significant’ in the split-half replication analyses.

## Issue with Within-sample split-half replication in present study

Recent work suggests that multinomial logistic regression models with a small number of events per variable (EPV; i.e., < 50), defined as the ratio of the number of observations within the smallest outcome category divided by the number of regression coefficient estimates, can lead to overfitting issues and reduce model predictive performance (1). Due to the small size of the maladaptive group (N_externalizing_ = 47 to 58, N_internalizing_ = 114 to 129 for discovery and replication samples), our split-half models had small EPVs, ranging from three to nine. As such, we focused on interpreting the results from analyses using the full sample in the main manuscript. Split-half replication findings are presented in our online supplementary document accessible at https://osf.io/5fdre/. It should be noted that the EPVs pertaining to the full sample analyses only ranged from six to 18; though an improvement from split-half replication analyses, the accuracy of estimates may be limited nevertheless.

# Appendix S6. Sample inclusion flow chart

**Figure S8.**

*Sample inclusion flow chart*


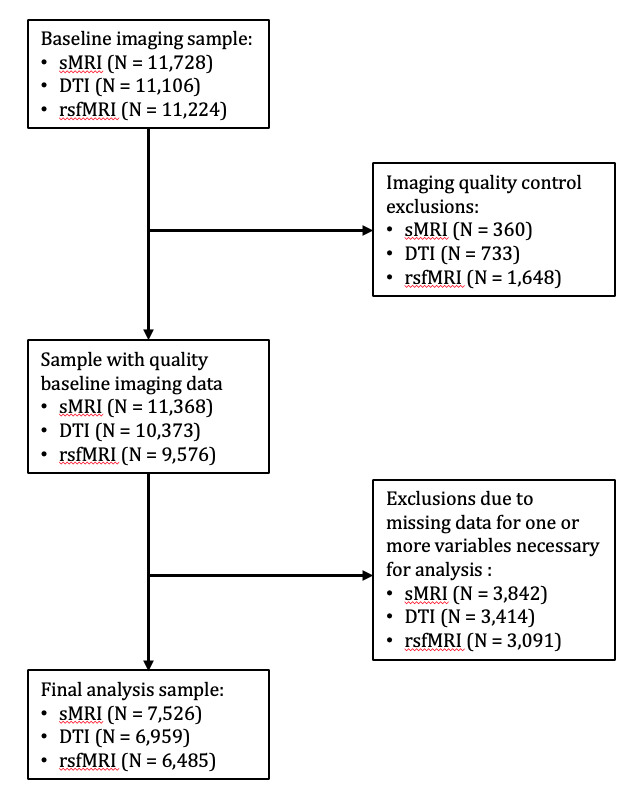


*Note*. Abbreviations explained, structural magnetic resonance imaging (sMRI), diffusion tensor imaging (DTI), resting-state functional MRI (rsfMRI).

# Appendix S7. Full sample results – Gray matter structure

## Main effects

### Table S1. Internalizing group comparisons

| **Variables** | **Group comparisons** | **B** | **95% CI** | **Odds** | **95% CI** | **FDRp** |
| --- | --- | --- | --- | --- | --- | --- |
| *Surface Area* |  |  |  |  |  |  |
| Banks of Superior Temporal Sulcus | MA : RES | -0.04 | [-0.14, 0.06] | 0.96 | [0.87, 1.06] | 0.867 |
|  | VU : RES | -0.05 | [-0.13, 0.04] | 0.96 | [0.88, 1.04] | 0.918 |
|  | HC : RES | -0.07 | [-0.15, 0.01] | 0.93 | [0.86, 1.01] | 0.834 |
| Caudal Anterior Cingulate Cortex | MA : RES | -0.02 | [-0.11, 0.08] | 0.98 | [0.89, 1.09] | 0.876 |
|  | VU : RES | 0.04 | [-0.05, 0.12] | 1.04 | [0.95, 1.13] | 0.955 |
|  | HC : RES | -0.01 | [-0.08, 0.07] | 0.99 | [0.92, 1.07] | 0.979 |
| Caudal Middle Frontal Gryus | MA : RES | 0.02 | [-0.09, 0.12] | 1.02 | [0.92, 1.13] | 0.876 |
|  | VU : RES | 0.02 | [-0.07, 0.11] | 1.02 | [0.93, 1.11] | 0.965 |
|  | HC : RES | 0.03 | [-0.06, 0.11] | 1.03 | [0.95, 1.11] | 0.979 |
| Cuneus | MA : RES | 0.12 | [0.03, 0.21] | 1.13 | [1.03, 1.23] | 0.350 |
|  | VU : RES | 0.05 | [-0.03, 0.13] | 1.06 | [0.97, 1.14] | 0.918 |
|  | HC : RES | 0.07 | [0, 0.14] | 1.07 | [1, 1.15] | 0.834 |
| Entorhinal Cortex | MA : RES | 0.04 | [-0.05, 0.13] | 1.04 | [0.95, 1.14] | 0.820 |
|  | VU : RES | 0.07 | [-0.01, 0.14] | 1.07 | [0.99, 1.15] | 0.697 |
|  | HC : RES | -0.02 | [-0.08, 0.05] | 0.98 | [0.92, 1.05] | 0.979 |
| Frontal Pole | MA : RES | -0.05 | [-0.16, 0.06] | 0.95 | [0.86, 1.06] | 0.820 |
|  | VU : RES | -0.02 | [-0.11, 0.08] | 0.98 | [0.9, 1.08] | 0.965 |
|  | HC : RES | -0.06 | [-0.14, 0.02] | 0.94 | [0.87, 1.02] | 0.834 |
| Fusiform Area | MA : RES | 0.07 | [-0.06, 0.19] | 1.07 | [0.95, 1.21] | 0.807 |
|  | VU : RES | 0.00 | [-0.1, 0.11] | 1.00 | [0.9, 1.12] | 0.965 |
|  | HC : RES | 0.00 | [-0.1, 0.09] | 1.00 | [0.91, 1.1] | 0.979 |
| Inferior Parietal Lobule | MA : RES | -0.02 | [-0.13, 0.08] | 0.98 | [0.88, 1.09] | 0.867 |
|  | VU : RES | 0.00 | [-0.09, 0.1] | 1.00 | [0.91, 1.1] | 0.965 |
|  | HC : RES | -0.02 | [-0.11, 0.06] | 0.98 | [0.9, 1.06] | 0.979 |
| Inferior Temporal Cortex | MA : RES | 0.04 | [-0.08, 0.15] | 1.04 | [0.92, 1.17] | 0.867 |
|  | VU : RES | 0.09 | [-0.01, 0.2] | 1.10 | [0.99, 1.22] | 0.697 |
|  | HC : RES | 0.04 | [-0.06, 0.13] | 1.04 | [0.94, 1.14] | 0.979 |
| Isthmus Cingulate Gyrus | MA : RES | -0.07 | [-0.17, 0.04] | 0.93 | [0.84, 1.04] | 0.756 |
|  | VU : RES | 0.02 | [-0.07, 0.1] | 1.02 | [0.93, 1.11] | 0.965 |
|  | HC : RES | -0.02 | [-0.1, 0.06] | 0.98 | [0.9, 1.06] | 0.979 |
| Insula | MA : RES | -0.07 | [-0.18, 0.04] | 0.93 | [0.83, 1.04] | 0.756 |
|  | VU : RES | 0.04 | [-0.06, 0.13] | 1.04 | [0.94, 1.14] | 0.965 |
|  | HC : RES | 0.00 | [-0.09, 0.09] | 1.00 | [0.92, 1.09] | 0.979 |
| Lingual Gyrus | MA : RES | 0.09 | [0, 0.18] | 1.09 | [1, 1.2] | 0.598 |
|  | VU : RES | 0.01 | [-0.07, 0.09] | 1.01 | [0.93, 1.09] | 0.965 |
|  | HC : RES | 0.00 | [-0.07, 0.07] | 1.00 | [0.93, 1.08] | 0.979 |
| Lateral Orbitofrontal Cortex | MA : RES | 0.03 | [-0.09, 0.15] | 1.03 | [0.91, 1.16] | 0.867 |
|  | VU : RES | 0.06 | [-0.04, 0.16] | 1.06 | [0.96, 1.18] | 0.918 |
|  | HC : RES | 0.03 | [-0.06, 0.13] | 1.03 | [0.94, 1.13] | 0.979 |
| Lateral Occipital Cortex | MA : RES | 0.06 | [-0.04, 0.17] | 1.06 | [0.96, 1.18] | 0.756 |
|  | VU : RES | 0.04 | [-0.05, 0.14] | 1.04 | [0.95, 1.15] | 0.944 |
|  | HC : RES | 0.04 | [-0.04, 0.12] | 1.04 | [0.96, 1.13] | 0.979 |
| Middle Temporal Gyrus | MA : RES | 0.03 | [-0.1, 0.15] | 1.03 | [0.91, 1.16] | 0.867 |
|  | VU : RES | 0.06 | [-0.05, 0.16] | 1.06 | [0.95, 1.18] | 0.918 |
|  | HC : RES | 0.01 | [-0.08, 0.11] | 1.01 | [0.92, 1.12] | 0.979 |
| Medial Orbitofrontal Cortex | MA : RES | -0.10 | [-0.23, 0.02] | 0.90 | [0.8, 1.02] | 0.756 |
|  | VU : RES | -0.01 | [-0.12, 0.1] | 0.99 | [0.89, 1.11] | 0.965 |
|  | HC : RES | -0.04 | [-0.14, 0.06] | 0.96 | [0.87, 1.06] | 0.979 |
| Paracentral Lobule | MA : RES | 0.02 | [-0.08, 0.12] | 1.02 | [0.92, 1.13] | 0.867 |
|  | VU : RES | 0.00 | [-0.08, 0.09] | 1.00 | [0.92, 1.1] | 0.965 |
|  | HC : RES | -0.01 | [-0.09, 0.07] | 0.99 | [0.91, 1.07] | 0.979 |
| Parahippocampal Gyrus | MA : RES | 0.03 | [-0.06, 0.12] | 1.03 | [0.94, 1.13] | 0.867 |
|  | VU : RES | 0.03 | [-0.06, 0.11] | 1.03 | [0.95, 1.11] | 0.965 |
|  | HC : RES | -0.02 | [-0.1, 0.05] | 0.98 | [0.91, 1.05] | 0.979 |
| Pars Orbitalis | MA : RES | 0.01 | [-0.1, 0.12] | 1.01 | [0.91, 1.12] | 0.920 |
|  | VU : RES | 0.04 | [-0.06, 0.13] | 1.04 | [0.94, 1.14] | 0.965 |
|  | HC : RES | -0.01 | [-0.1, 0.07] | 0.99 | [0.91, 1.08] | 0.979 |
| Pars Opercularis | MA : RES | 0.04 | [-0.06, 0.13] | 1.04 | [0.94, 1.14] | 0.867 |
|  | VU : RES | 0.00 | [-0.08, 0.09] | 1.00 | [0.92, 1.09] | 0.965 |
|  | HC : RES | -0.02 | [-0.09, 0.06] | 0.98 | [0.91, 1.06] | 0.979 |
| Pars Triangularis | MA : RES | 0.01 | [-0.09, 0.1] | 1.01 | [0.91, 1.11] | 0.920 |
|  | VU : RES | 0.01 | [-0.07, 0.1] | 1.02 | [0.93, 1.1] | 0.965 |
|  | HC : RES | -0.02 | [-0.09, 0.05] | 0.98 | [0.91, 1.06] | 0.979 |
| Precuneus | MA : RES | -0.02 | [-0.14, 0.1] | 0.98 | [0.87, 1.11] | 0.876 |
|  | VU : RES | 0.01 | [-0.09, 0.12] | 1.01 | [0.91, 1.12] | 0.965 |
|  | HC : RES | -0.05 | [-0.14, 0.05] | 0.95 | [0.87, 1.05] | 0.979 |
| Pericalcarine Cortex | MA : RES | 0.07 | [-0.02, 0.15] | 1.07 | [0.98, 1.16] | 0.756 |
|  | VU : RES | 0.01 | [-0.06, 0.08] | 1.01 | [0.94, 1.09] | 0.965 |
|  | HC : RES | 0.02 | [-0.04, 0.09] | 1.02 | [0.96, 1.09] | 0.979 |
| Postcentral Gyrus | MA : RES | -0.08 | [-0.2, 0.04] | 0.92 | [0.81, 1.04] | 0.756 |
|  | VU : RES | 0.03 | [-0.08, 0.14] | 1.03 | [0.93, 1.15] | 0.965 |
|  | HC : RES | -0.02 | [-0.12, 0.08] | 0.98 | [0.89, 1.08] | 0.979 |
| Precentral Gyrus | MA : RES | -0.01 | [-0.13, 0.11] | 0.99 | [0.88, 1.12] | 0.920 |
|  | VU : RES | 0.06 | [-0.04, 0.17] | 1.07 | [0.96, 1.18] | 0.918 |
|  | HC : RES | 0.08 | [-0.02, 0.17] | 1.08 | [0.98, 1.18] | 0.834 |
| Posterior Cingulate Cortex | MA : RES | -0.07 | [-0.18, 0.04] | 0.94 | [0.84, 1.04] | 0.756 |
|  | VU : RES | 0.09 | [-0.01, 0.18] | 1.09 | [0.99, 1.2] | 0.697 |
|  | HC : RES | 0.00 | [-0.09, 0.08] | 1.00 | [0.92, 1.08] | 0.979 |
| Rostral Anterior Cingulate Cortex | MA : RES | 0.03 | [-0.08, 0.14] | 1.03 | [0.92, 1.15] | 0.867 |
|  | VU : RES | 0.05 | [-0.04, 0.15] | 1.05 | [0.96, 1.16] | 0.918 |
|  | HC : RES | -0.01 | [-0.1, 0.08] | 0.99 | [0.91, 1.08] | 0.979 |
| Rostral Middle Frontal Gyrus | MA : RES | -0.04 | [-0.16, 0.07] | 0.96 | [0.86, 1.08] | 0.867 |
|  | VU : RES | -0.05 | [-0.15, 0.05] | 0.95 | [0.86, 1.05] | 0.944 |
|  | HC : RES | -0.06 | [-0.16, 0.03] | 0.94 | [0.86, 1.03] | 0.834 |
| Supramarginal Gyrus | MA : RES | -0.05 | [-0.16, 0.06] | 0.95 | [0.85, 1.06] | 0.820 |
|  | VU : RES | 0.00 | [-0.09, 0.1] | 1.00 | [0.91, 1.1] | 0.965 |
|  | HC : RES | -0.06 | [-0.14, 0.03] | 0.94 | [0.87, 1.03] | 0.834 |
| Superior Frontal Gyrus | MA : RES | -0.13 | [-0.25, 0] | 0.88 | [0.78, 1] | 0.598 |
|  | VU : RES | -0.09 | [-0.2, 0.02] | 0.92 | [0.82, 1.02] | 0.861 |
|  | HC : RES | -0.07 | [-0.17, 0.03] | 0.94 | [0.85, 1.03] | 0.834 |
| Superior Parietal Lobule | MA : RES | -0.01 | [-0.12, 0.1] | 0.99 | [0.89, 1.1] | 0.898 |
|  | VU : RES | -0.01 | [-0.11, 0.08] | 0.99 | [0.9, 1.08] | 0.965 |
|  | HC : RES | -0.05 | [-0.13, 0.03] | 0.95 | [0.87, 1.04] | 0.937 |
| Superior Temporal Gyrus | MA : RES | -0.08 | [-0.2, 0.04] | 0.93 | [0.82, 1.05] | 0.756 |
|  | VU : RES | -0.02 | [-0.13, 0.08] | 0.98 | [0.88, 1.09] | 0.965 |
|  | HC : RES | -0.07 | [-0.16, 0.03] | 0.94 | [0.85, 1.03] | 0.834 |
| Temporal Pole | MA : RES | 0.03 | [-0.07, 0.13] | 1.03 | [0.93, 1.14] | 0.867 |
|  | VU : RES | 0.09 | [0.01, 0.18] | 1.10 | [1.01, 1.19] | 0.697 |
|  | HC : RES | -0.01 | [-0.08, 0.07] | 0.99 | [0.92, 1.07] | 0.979 |
| Transverse Temporal Gyrus | MA : RES | 0.02 | [-0.08, 0.11] | 1.02 | [0.93, 1.12] | 0.867 |
|  | VU : RES | 0.03 | [-0.06, 0.11] | 1.03 | [0.95, 1.11] | 0.965 |
|  | HC : RES | 0.01 | [-0.06, 0.09] | 1.01 | [0.94, 1.09] | 0.979 |
| *Cortical Thickness* |  |  |  |  |  |  |
| Banks of Superior Temporal Sulcus | MA : RES | 0.07 | [-0.01, 0.15] | 1.07 | [0.99, 1.16] | 0.585 |
|  | VU : RES | 0.05 | [-0.02, 0.12] | 1.05 | [0.98, 1.13] | 0.548 |
|  | HC : RES | 0.06 | [0, 0.13] | 1.07 | [1, 1.13] | 0.287 |
| Caudal Anterior Cingulate Cortex | MA : RES | -0.07 | [-0.15, 0.01] | 0.93 | [0.86, 1.01] | 0.585 |
|  | VU : RES | -0.02 | [-0.09, 0.05] | 0.98 | [0.92, 1.05] | 0.826 |
|  | HC : RES | 0.03 | [-0.03, 0.09] | 1.03 | [0.97, 1.09] | 0.676 |
| Caudal Middle Frontal Gryus | MA : RES | 0.01 | [-0.07, 0.09] | 1.01 | [0.93, 1.09] | 0.971 |
|  | VU : RES | 0.04 | [-0.03, 0.11] | 1.04 | [0.97, 1.11] | 0.662 |
|  | HC : RES | 0.03 | [-0.04, 0.09] | 1.03 | [0.96, 1.09] | 0.676 |
| Cuneus | MA : RES | 0.00 | [-0.08, 0.08] | 1.00 | [0.92, 1.08] | 0.989 |
|  | VU : RES | 0.00 | [-0.07, 0.07] | 1.00 | [0.93, 1.07] | 0.991 |
|  | HC : RES | 0.05 | [-0.01, 0.11] | 1.05 | [0.99, 1.12] | 0.287 |
| Entorhinal Cortex | MA : RES | 0.02 | [-0.06, 0.1] | 1.02 | [0.94, 1.11] | 0.899 |
|  | VU : RES | -0.01 | [-0.08, 0.06] | 0.99 | [0.92, 1.06] | 0.852 |
|  | HC : RES | 0.02 | [-0.05, 0.08] | 1.02 | [0.96, 1.08] | 0.768 |
| Frontal Pole | MA : RES | 0.00 | [-0.08, 0.08] | 1.00 | [0.92, 1.08] | 0.998 |
|  | VU : RES | -0.02 | [-0.09, 0.05] | 0.98 | [0.91, 1.05] | 0.805 |
|  | HC : RES | 0.01 | [-0.05, 0.07] | 1.01 | [0.95, 1.07] | 0.813 |
| Fusiform Area | MA : RES | 0.05 | [-0.03, 0.13] | 1.05 | [0.97, 1.13] | 0.717 |
|  | VU : RES | 0.02 | [-0.05, 0.09] | 1.02 | [0.95, 1.1] | 0.805 |
|  | HC : RES | 0.04 | [-0.02, 0.1] | 1.04 | [0.98, 1.11] | 0.424 |
| Inferior Parietal Lobule | MA : RES | 0.05 | [-0.03, 0.13] | 1.05 | [0.97, 1.14] | 0.737 |
|  | VU : RES | 0.01 | [-0.06, 0.09] | 1.01 | [0.94, 1.09] | 0.852 |
|  | HC : RES | 0.06 | [0, 0.13] | 1.06 | [1, 1.13] | 0.287 |
| Inferior Temporal Cortex | MA : RES | 0.01 | [-0.07, 0.09] | 1.01 | [0.94, 1.1] | 0.899 |
|  | VU : RES | 0.01 | [-0.06, 0.08] | 1.01 | [0.94, 1.08] | 0.862 |
|  | HC : RES | 0.03 | [-0.03, 0.09] | 1.03 | [0.97, 1.09] | 0.676 |
| Isthmus Cingulate Gyrus | MA : RES | 0.06 | [-0.02, 0.14] | 1.06 | [0.98, 1.15] | 0.585 |
|  | VU : RES | -0.04 | [-0.1, 0.03] | 0.97 | [0.9, 1.03] | 0.662 |
|  | HC : RES | -0.01 | [-0.07, 0.05] | 0.99 | [0.93, 1.05] | 0.778 |
| Insula | MA : RES | -0.01 | [-0.09, 0.07] | 0.99 | [0.91, 1.07] | 0.899 |
|  | VU : RES | -0.08 | [-0.15, -0.01] | 0.93 | [0.86, 0.99] | 0.540 |
|  | HC : RES | 0.00 | [-0.06, 0.06] | 1.00 | [0.94, 1.06] | 0.995 |
| Lingual Gyrus | MA : RES | 0.02 | [-0.06, 0.1] | 1.02 | [0.94, 1.11] | 0.899 |
|  | VU : RES | 0.06 | [-0.01, 0.13] | 1.06 | [0.99, 1.14] | 0.548 |
|  | HC : RES | 0.07 | [0.01, 0.13] | 1.07 | [1.01, 1.14] | 0.287 |
| Lateral Orbitofrontal Cortex | MA : RES | -0.07 | [-0.14, 0.01] | 0.94 | [0.87, 1.01] | 0.585 |
|  | VU : RES | -0.05 | [-0.12, 0.02] | 0.95 | [0.89, 1.02] | 0.548 |
|  | HC : RES | 0.02 | [-0.04, 0.08] | 1.02 | [0.96, 1.08] | 0.768 |
| Lateral Occipital Cortex | MA : RES | 0.05 | [-0.04, 0.14] | 1.05 | [0.96, 1.15] | 0.751 |
|  | VU : RES | 0.07 | [0, 0.15] | 1.08 | [1, 1.16] | 0.548 |
|  | HC : RES | 0.11 | [0.04, 0.18] | 1.11 | [1.04, 1.19] | **0.044** |
| Middle Temporal Gyrus | MA : RES | 0.05 | [-0.03, 0.14] | 1.06 | [0.97, 1.15] | 0.717 |
|  | VU : RES | 0.00 | [-0.07, 0.08] | 1.00 | [0.93, 1.08] | 0.991 |
|  | HC : RES | 0.02 | [-0.05, 0.08] | 1.02 | [0.95, 1.09] | 0.768 |
| Medial Orbitofrontal Cortex | MA : RES | -0.04 | [-0.12, 0.04] | 0.96 | [0.89, 1.04] | 0.751 |
|  | VU : RES | -0.04 | [-0.11, 0.03] | 0.96 | [0.89, 1.03] | 0.662 |
|  | HC : RES | 0.02 | [-0.05, 0.08] | 1.02 | [0.95, 1.08] | 0.768 |
| Paracentral Lobule | MA : RES | -0.03 | [-0.11, 0.05] | 0.97 | [0.9, 1.05] | 0.866 |
|  | VU : RES | 0.03 | [-0.04, 0.1] | 1.03 | [0.96, 1.1] | 0.714 |
|  | HC : RES | 0.06 | [0, 0.12] | 1.06 | [1, 1.13] | 0.287 |
| Parahippocampal Gyrus | MA : RES | 0.06 | [-0.02, 0.14] | 1.06 | [0.98, 1.15] | 0.659 |
|  | VU : RES | 0.04 | [-0.03, 0.11] | 1.04 | [0.97, 1.11] | 0.662 |
|  | HC : RES | 0.02 | [-0.04, 0.08] | 1.02 | [0.96, 1.08] | 0.768 |
| Pars Orbitalis | MA : RES | 0.02 | [-0.06, 0.09] | 1.02 | [0.94, 1.1] | 0.899 |
|  | VU : RES | -0.03 | [-0.1, 0.04] | 0.97 | [0.91, 1.04] | 0.745 |
|  | HC : RES | -0.01 | [-0.07, 0.05] | 0.99 | [0.93, 1.05] | 0.813 |
| Pars Opercularis | MA : RES | 0.00 | [-0.08, 0.08] | 1.00 | [0.92, 1.08] | 0.998 |
|  | VU : RES | -0.04 | [-0.11, 0.03] | 0.96 | [0.9, 1.03] | 0.662 |
|  | HC : RES | -0.05 | [-0.12, 0.01] | 0.95 | [0.89, 1.01] | 0.287 |
| Pars Triangularis | MA : RES | 0.05 | [-0.03, 0.13] | 1.05 | [0.97, 1.13] | 0.717 |
|  | VU : RES | -0.02 | [-0.08, 0.05] | 0.98 | [0.92, 1.05] | 0.852 |
|  | HC : RES | 0.02 | [-0.05, 0.08] | 1.02 | [0.96, 1.08] | 0.768 |
| Precuneus | MA : RES | 0.02 | [-0.06, 0.1] | 1.02 | [0.94, 1.1] | 0.899 |
|  | VU : RES | 0.01 | [-0.06, 0.08] | 1.01 | [0.94, 1.08] | 0.862 |
|  | HC : RES | 0.05 | [-0.01, 0.11] | 1.05 | [0.99, 1.12] | 0.287 |
| Pericalcarine Cortex | MA : RES | -0.03 | [-0.11, 0.05] | 0.97 | [0.9, 1.05] | 0.889 |
|  | VU : RES | 0.00 | [-0.07, 0.07] | 1.00 | [0.93, 1.07] | 0.991 |
|  | HC : RES | 0.04 | [-0.03, 0.1] | 1.04 | [0.98, 1.11] | 0.509 |
| Postcentral Gyrus | MA : RES | 0.09 | [0.01, 0.17] | 1.10 | [1.01, 1.19] | 0.585 |
|  | VU : RES | 0.08 | [0.01, 0.15] | 1.09 | [1.01, 1.17] | 0.540 |
|  | HC : RES | 0.11 | [0.05, 0.17] | 1.12 | [1.05, 1.19] | **0.030** |
| Precentral Gyrus | MA : RES | -0.02 | [-0.09, 0.06] | 0.98 | [0.91, 1.07] | 0.899 |
|  | VU : RES | 0.01 | [-0.06, 0.08] | 1.01 | [0.95, 1.09] | 0.852 |
|  | HC : RES | 0.01 | [-0.05, 0.08] | 1.01 | [0.95, 1.08] | 0.768 |
| Posterior Cingulate Cortex | MA : RES | 0.03 | [-0.05, 0.11] | 1.03 | [0.96, 1.12] | 0.866 |
|  | VU : RES | 0.06 | [-0.01, 0.13] | 1.06 | [0.99, 1.14] | 0.548 |
|  | HC : RES | 0.06 | [0, 0.12] | 1.06 | [1, 1.13] | 0.287 |
| Rostral Anterior Cingulate Cortex | MA : RES | 0.01 | [-0.07, 0.09] | 1.01 | [0.94, 1.1] | 0.899 |
|  | VU : RES | -0.05 | [-0.12, 0.02] | 0.95 | [0.88, 1.02] | 0.548 |
|  | HC : RES | 0.01 | [-0.05, 0.08] | 1.01 | [0.95, 1.08] | 0.768 |
| Rostral Middle Frontal Gyrus | MA : RES | -0.02 | [-0.09, 0.06] | 0.98 | [0.91, 1.07] | 0.899 |
|  | VU : RES | 0.00 | [-0.07, 0.07] | 1.00 | [0.93, 1.07] | 0.991 |
|  | HC : RES | 0.03 | [-0.04, 0.09] | 1.03 | [0.96, 1.09] | 0.676 |
| Supramarginal Gyrus | MA : RES | 0.07 | [-0.02, 0.15] | 1.07 | [0.98, 1.17] | 0.585 |
|  | VU : RES | 0.04 | [-0.04, 0.11] | 1.04 | [0.96, 1.12] | 0.662 |
|  | HC : RES | 0.05 | [-0.02, 0.11] | 1.05 | [0.98, 1.12] | 0.418 |
| Superior Frontal Gyrus | MA : RES | -0.02 | [-0.1, 0.06] | 0.98 | [0.91, 1.06] | 0.899 |
|  | VU : RES | 0.03 | [-0.04, 0.1] | 1.03 | [0.96, 1.1] | 0.745 |
|  | HC : RES | 0.05 | [-0.01, 0.11] | 1.05 | [0.99, 1.12] | 0.287 |
| Superior Parietal Lobule | MA : RES | 0.00 | [-0.08, 0.08] | 1.00 | [0.92, 1.08] | 0.989 |
|  | VU : RES | 0.02 | [-0.05, 0.09] | 1.02 | [0.95, 1.09] | 0.826 |
|  | HC : RES | 0.06 | [-0.01, 0.12] | 1.06 | [0.99, 1.13] | 0.287 |
| Superior Temporal Gyrus | MA : RES | 0.08 | [0, 0.16] | 1.09 | [1, 1.18] | 0.585 |
|  | VU : RES | 0.03 | [-0.04, 0.1] | 1.04 | [0.97, 1.11] | 0.662 |
|  | HC : RES | 0.05 | [-0.01, 0.12] | 1.06 | [0.99, 1.12] | 0.287 |
| Temporal Pole | MA : RES | 0.02 | [-0.06, 0.1] | 1.02 | [0.94, 1.1] | 0.899 |
|  | VU : RES | -0.05 | [-0.12, 0.02] | 0.95 | [0.89, 1.02] | 0.548 |
|  | HC : RES | -0.01 | [-0.07, 0.05] | 0.99 | [0.93, 1.05] | 0.813 |
| Transverse Temporal Gyrus | MA : RES | 0.04 | [-0.04, 0.12] | 1.04 | [0.96, 1.13] | 0.751 |
|  | VU : RES | -0.04 | [-0.11, 0.03] | 0.96 | [0.9, 1.03] | 0.662 |
|  | HC : RES | 0.03 | [-0.03, 0.09] | 1.03 | [0.97, 1.09] | 0.676 |
| *Subcortical Volume* |  |  |  |  |  |  |
| Accumbens | MA : RES | -0.08 | [-0.18, 0.02] | 0.92 | [0.83, 1.02] | 0.371 |
|  | VU : RES | -0.03 | [-0.11, 0.06] | 0.97 | [0.89, 1.06] | 0.880 |
|  | HC : RES | -0.01 | [-0.09, 0.06] | 0.99 | [0.91, 1.07] | 0.756 |
| Amygdala | MA : RES | 0.01 | [-0.1, 0.12] | 1.01 | [0.91, 1.13] | 0.869 |
|  | VU : RES | 0.04 | [-0.05, 0.14] | 1.05 | [0.95, 1.15] | 0.838 |
|  | HC : RES | -0.02 | [-0.1, 0.07] | 0.98 | [0.9, 1.07] | 0.756 |
| Caudate | MA : RES | 0.01 | [-0.08, 0.1] | 1.01 | [0.92, 1.11] | 0.869 |
|  | VU : RES | -0.06 | [-0.14, 0.02] | 0.94 | [0.87, 1.02] | 0.536 |
|  | HC : RES | -0.03 | [-0.11, 0.04] | 0.97 | [0.9, 1.04] | 0.756 |
| Hippocampus | MA : RES | 0.06 | [-0.05, 0.16] | 1.06 | [0.95, 1.18] | 0.557 |
|  | VU : RES | 0.12 | [0.03, 0.22] | 1.13 | [1.03, 1.24] | 0.065 |
|  | HC : RES | 0.05 | [-0.03, 0.14] | 1.06 | [0.97, 1.15] | 0.756 |
| Pallidum | MA : RES | -0.07 | [-0.17, 0.03] | 0.93 | [0.85, 1.03] | 0.371 |
|  | VU : RES | -0.01 | [-0.1, 0.07] | 0.99 | [0.91, 1.07] | 0.880 |
|  | HC : RES | -0.02 | [-0.09, 0.06] | 0.98 | [0.91, 1.06] | 0.756 |
| Putamen | MA : RES | -0.02 | [-0.11, 0.08] | 0.98 | [0.89, 1.08] | 0.869 |
|  | VU : RES | 0.01 | [-0.07, 0.1] | 1.01 | [0.93, 1.1] | 0.880 |
|  | HC : RES | 0.01 | [-0.06, 0.09] | 1.01 | [0.94, 1.09] | 0.756 |
| Thalamus | MA : RES | -0.09 | [-0.2, 0.03] | 0.92 | [0.82, 1.03] | 0.371 |
|  | VU : RES | 0.00 | [-0.1, 0.1] | 1.00 | [0.91, 1.11] | 0.962 |
|  | HC : RES | -0.03 | [-0.12, 0.06] | 0.97 | [0.89, 1.06] | 0.756 |

### Table S2. Externalizing group comparisons

| **Variables** | **Group comparisons** | **B** | **95% CI** | **Odds** | **95% CI** | ***p*FDR** |
| --- | --- | --- | --- | --- | --- | --- |
| *Surface area* |  |  |  |  |  |  |
| Banks of Superior Temporal Sulcus | MA : RES | -0.14 | [-0.39, 0.11] | 0.87 | [0.67, 1.12] | 0.782 |
|  | VU : RES | -0.10 | [-0.34, 0.14] | 0.91 | [0.71, 1.15] | 0.905 |
|  | HC : RES | -0.04 | [-0.12, 0.03] | 0.96 | [0.89, 1.03] | 0.776 |
| Caudal Anterior Cingulate Cortex | MA : RES | -0.05 | [-0.29, 0.2] | 0.95 | [0.75, 1.22] | 0.848 |
|  | VU : RES | -0.16 | [-0.39, 0.08] | 0.86 | [0.68, 1.09] | 0.905 |
|  | HC : RES | -0.01 | [-0.08, 0.07] | 0.99 | [0.92, 1.07] | 0.982 |
| Caudal Middle Frontal Gyrus | MA : RES | -0.06 | [-0.32, 0.21] | 0.94 | [0.73, 1.23] | 0.847 |
|  | VU : RES | -0.15 | [-0.4, 0.1] | 0.86 | [0.67, 1.1] | 0.905 |
|  | HC : RES | 0.00 | [-0.08, 0.08] | 1.00 | [0.93, 1.09] | 0.982 |
| Cuneus | MA : RES | 0.09 | [-0.13, 0.31] | 1.09 | [0.88, 1.36] | 0.816 |
|  | VU : RES | 0.11 | [-0.1, 0.32] | 1.12 | [0.91, 1.38] | 0.905 |
|  | HC : RES | -0.01 | [-0.08, 0.06] | 0.99 | [0.93, 1.07] | 0.982 |
| Entorhinal Cortex | MA : RES | 0.04 | [-0.18, 0.25] | 1.04 | [0.84, 1.29] | 0.848 |
|  | VU : RES | 0.06 | [-0.15, 0.26] | 1.06 | [0.86, 1.3] | 0.938 |
|  | HC : RES | -0.01 | [-0.07, 0.06] | 0.99 | [0.93, 1.06] | 0.982 |
| Frontal Pole | MA : RES | 0.09 | [-0.17, 0.36] | 1.10 | [0.85, 1.43] | 0.816 |
|  | VU : RES | -0.14 | [-0.39, 0.12] | 0.87 | [0.68, 1.12] | 0.905 |
|  | HC : RES | -0.05 | [-0.13, 0.04] | 0.96 | [0.88, 1.04] | 0.776 |
| Fusiform Area | MA : RES | 0.15 | [-0.15, 0.45] | 1.16 | [0.86, 1.57] | 0.782 |
|  | VU : RES | -0.12 | [-0.41, 0.17] | 0.89 | [0.67, 1.19] | 0.905 |
|  | HC : RES | -0.03 | [-0.13, 0.06] | 0.97 | [0.88, 1.06] | 0.788 |
| Inferior Parietal Lobule | MA : RES | -0.21 | [-0.48, 0.06] | 0.81 | [0.62, 1.06] | 0.453 |
|  | VU : RES | -0.29 | [-0.55, -0.04] | 0.75 | [0.58, 0.97] | 0.876 |
|  | HC : RES | 0.00 | [-0.08, 0.08] | 1.00 | [0.92, 1.09] | 0.983 |
| Inferior Temporal Cortex | MA : RES | -0.34 | [-0.63, -0.05] | 0.71 | [0.53, 0.95] | 0.383 |
|  | VU : RES | -0.02 | [-0.3, 0.25] | 0.98 | [0.74, 1.29] | 0.977 |
|  | HC : RES | 0.09 | [0, 0.18] | 1.09 | [1, 1.19] | 0.776 |
| Isthmus Cingulate Gyrus | MA : RES | -0.03 | [-0.29, 0.22] | 0.97 | [0.75, 1.25] | 0.848 |
|  | VU : RES | 0.02 | [-0.22, 0.27] | 1.02 | [0.81, 1.3] | 0.977 |
|  | HC : RES | 0.00 | [-0.08, 0.07] | 1.00 | [0.92, 1.08] | 0.982 |
| Insula | MA : RES | 0.23 | [-0.05, 0.5] | 1.26 | [0.96, 1.65] | 0.433 |
|  | VU : RES | -0.05 | [-0.32, 0.21] | 0.95 | [0.73, 1.23] | 0.977 |
|  | HC : RES | 0.03 | [-0.06, 0.11] | 1.03 | [0.95, 1.12] | 0.827 |
| Lingual Gyrus | MA : RES | 0.25 | [0.04, 0.47] | 1.29 | [1.04, 1.6] | 0.383 |
|  | VU : RES | -0.01 | [-0.23, 0.2] | 0.99 | [0.8, 1.22] | 0.977 |
|  | HC : RES | -0.05 | [-0.12, 0.02] | 0.95 | [0.89, 1.02] | 0.776 |
| Lateral Orbitofrontal Cortex | MA : RES | 0.10 | [-0.2, 0.4] | 1.10 | [0.82, 1.49] | 0.826 |
|  | VU : RES | -0.10 | [-0.38, 0.18] | 0.90 | [0.68, 1.2] | 0.917 |
|  | HC : RES | 0.04 | [-0.05, 0.14] | 1.05 | [0.95, 1.15] | 0.776 |
| Lateral Occipital Cortex | MA : RES | 0.08 | [-0.18, 0.33] | 1.08 | [0.83, 1.4] | 0.847 |
|  | VU : RES | -0.02 | [-0.27, 0.23] | 0.98 | [0.77, 1.26] | 0.977 |
|  | HC : RES | -0.02 | [-0.1, 0.06] | 0.98 | [0.91, 1.07] | 0.969 |
| Middle Temporal Gyrus | MA : RES | -0.31 | [-0.62, -0.01] | 0.73 | [0.54, 0.99] | 0.383 |
|  | VU : RES | -0.01 | [-0.3, 0.27] | 0.99 | [0.74, 1.31] | 0.977 |
|  | HC : RES | 0.07 | [-0.02, 0.16] | 1.07 | [0.98, 1.18] | 0.776 |
| Medial Orbitofrontal Cortex | MA : RES | 0.14 | [-0.17, 0.46] | 1.16 | [0.85, 1.58] | 0.782 |
|  | VU : RES | -0.09 | [-0.39, 0.21] | 0.91 | [0.68, 1.23] | 0.935 |
|  | HC : RES | -0.01 | [-0.1, 0.09] | 0.99 | [0.9, 1.09] | 0.982 |
| Paracentral Lobule | MA : RES | -0.04 | [-0.3, 0.21] | 0.96 | [0.74, 1.24] | 0.848 |
|  | VU : RES | -0.14 | [-0.38, 0.1] | 0.87 | [0.68, 1.11] | 0.905 |
|  | HC : RES | 0.00 | [-0.07, 0.08] | 1.00 | [0.93, 1.08] | 0.982 |
| Parahippocampal Gyrus | MA : RES | 0.23 | [0.01, 0.46] | 1.26 | [1.01, 1.58] | 0.383 |
|  | VU : RES | 0.02 | [-0.2, 0.24] | 1.02 | [0.82, 1.27] | 0.977 |
|  | HC : RES | -0.03 | [-0.1, 0.04] | 0.97 | [0.9, 1.04] | 0.776 |
| Pars Orbitalis | MA : RES | 0.06 | [-0.21, 0.33] | 1.07 | [0.81, 1.4] | 0.847 |
|  | VU : RES | -0.01 | [-0.27, 0.24] | 0.99 | [0.76, 1.28] | 0.977 |
|  | HC : RES | 0.02 | [-0.07, 0.1] | 1.02 | [0.94, 1.11] | 0.969 |
| Pars Opercularis | MA : RES | -0.16 | [-0.4, 0.09] | 0.86 | [0.67, 1.1] | 0.739 |
|  | VU : RES | -0.13 | [-0.37, 0.1] | 0.87 | [0.69, 1.1] | 0.905 |
|  | HC : RES | -0.04 | [-0.11, 0.03] | 0.96 | [0.89, 1.03] | 0.776 |
| Pars Triangularis | MA : RES | -0.02 | [-0.25, 0.22] | 0.98 | [0.78, 1.25] | 0.913 |
|  | VU : RES | 0.04 | [-0.18, 0.26] | 1.04 | [0.83, 1.3] | 0.977 |
|  | HC : RES | -0.02 | [-0.09, 0.05] | 0.98 | [0.91, 1.05] | 0.898 |
| Precuneus | MA : RES | 0.07 | [-0.24, 0.37] | 1.07 | [0.79, 1.45] | 0.847 |
|  | VU : RES | -0.15 | [-0.44, 0.14] | 0.86 | [0.65, 1.16] | 0.905 |
|  | HC : RES | -0.04 | [-0.13, 0.06] | 0.96 | [0.88, 1.06] | 0.776 |
| Pericalcarine Cortex | MA : RES | 0.09 | [-0.1, 0.29] | 1.10 | [0.9, 1.34] | 0.782 |
|  | VU : RES | -0.01 | [-0.2, 0.19] | 0.99 | [0.82, 1.2] | 0.977 |
|  | HC : RES | -0.04 | [-0.11, 0.02] | 0.96 | [0.9, 1.02] | 0.776 |
| Postcentral Gyrus | MA : RES | -0.07 | [-0.38, 0.24] | 0.93 | [0.68, 1.27] | 0.847 |
|  | VU : RES | -0.04 | [-0.33, 0.26] | 0.97 | [0.72, 1.29] | 0.977 |
|  | HC : RES | 0.01 | [-0.09, 0.1] | 1.01 | [0.92, 1.11] | 0.982 |
| Precentral Gyrus | MA : RES | -0.15 | [-0.45, 0.16] | 0.86 | [0.64, 1.17] | 0.782 |
|  | VU : RES | 0.00 | [-0.29, 0.28] | 1.00 | [0.75, 1.32] | 0.977 |
|  | HC : RES | 0.05 | [-0.04, 0.14] | 1.05 | [0.96, 1.15] | 0.776 |
| Posterior Cingulate Cortex | MA : RES | -0.06 | [-0.33, 0.21] | 0.94 | [0.72, 1.24] | 0.847 |
|  | VU : RES | 0.10 | [-0.16, 0.35] | 1.10 | [0.86, 1.42] | 0.905 |
|  | HC : RES | 0.03 | [-0.05, 0.12] | 1.04 | [0.95, 1.12] | 0.776 |
| Rostral Anterior Cingulate Cortex | MA : RES | 0.13 | [-0.15, 0.4] | 1.13 | [0.86, 1.49] | 0.782 |
|  | VU : RES | -0.15 | [-0.42, 0.11] | 0.86 | [0.66, 1.12] | 0.905 |
|  | HC : RES | 0.04 | [-0.04, 0.13] | 1.04 | [0.96, 1.13] | 0.776 |
| Rostral Middle Frontal Gyrus | MA : RES | 0.10 | [-0.18, 0.38] | 1.11 | [0.84, 1.46] | 0.816 |
|  | VU : RES | -0.17 | [-0.44, 0.11] | 0.85 | [0.64, 1.11] | 0.905 |
|  | HC : RES | -0.05 | [-0.14, 0.04] | 0.95 | [0.87, 1.04] | 0.776 |
| Supramarginal Gyrus | MA : RES | -0.26 | [-0.54, 0.02] | 0.77 | [0.58, 1.02] | 0.433 |
|  | VU : RES | -0.18 | [-0.44, 0.09] | 0.84 | [0.65, 1.09] | 0.905 |
|  | HC : RES | -0.04 | [-0.13, 0.04] | 0.96 | [0.88, 1.04] | 0.776 |
| Superior Frontal Gyrus | MA : RES | -0.01 | [-0.33, 0.3] | 0.99 | [0.72, 1.35] | 0.935 |
|  | VU : RES | -0.10 | [-0.4, 0.2] | 0.90 | [0.67, 1.22] | 0.920 |
|  | HC : RES | -0.05 | [-0.15, 0.05] | 0.95 | [0.86, 1.05] | 0.776 |
| Superior Parietal Lobule | MA : RES | -0.23 | [-0.49, 0.04] | 0.80 | [0.61, 1.04] | 0.433 |
|  | VU : RES | -0.18 | [-0.43, 0.07] | 0.84 | [0.65, 1.08] | 0.905 |
|  | HC : RES | -0.04 | [-0.12, 0.04] | 0.96 | [0.89, 1.05] | 0.776 |
| Superior Temporal Gyrus | MA : RES | -0.13 | [-0.44, 0.18] | 0.88 | [0.65, 1.19] | 0.816 |
|  | VU : RES | -0.12 | [-0.41, 0.17] | 0.89 | [0.66, 1.18] | 0.905 |
|  | HC : RES | -0.08 | [-0.17, 0.01] | 0.92 | [0.84, 1.01] | 0.776 |
| Temporal Pole | MA : RES | 0.21 | [-0.03, 0.45] | 1.23 | [0.97, 1.57] | 0.433 |
|  | VU : RES | 0.11 | [-0.12, 0.34] | 1.11 | [0.88, 1.4] | 0.905 |
|  | HC : RES | -0.01 | [-0.09, 0.06] | 0.99 | [0.91, 1.06] | 0.969 |
| Transverse Temporal Gyrus | MA : RES | -0.03 | [-0.27, 0.21] | 0.97 | [0.76, 1.23] | 0.848 |
|  | VU : RES | -0.02 | [-0.24, 0.21] | 0.98 | [0.79, 1.23] | 0.977 |
|  | HC : RES | -0.03 | [-0.1, 0.04] | 0.97 | [0.9, 1.04] | 0.776 |
| *Cortical thickness* |  |  |  |  |  |  |
| Banks of Superior Temporal Sulcus | MA : RES | -0.07 | [-0.27, 0.12] | 0.93 | [0.77, 1.12] | 0.688 |
|  | VU : RES | 0.09 | [-0.1, 0.27] | 1.09 | [0.91, 1.31] | 0.639 |
|  | HC : RES | 0.07 | [0.01, 0.13] | 1.07 | [1.01, 1.13] | 0.208 |
| Caudal Anterior Cingulate Cortex | MA : RES | 0.10 | [-0.08, 0.29] | 1.11 | [0.92, 1.34] | 0.647 |
|  | VU : RES | 0.12 | [-0.06, 0.3] | 1.13 | [0.94, 1.35] | 0.571 |
|  | HC : RES | 0.01 | [-0.05, 0.07] | 1.01 | [0.95, 1.07] | 0.832 |
| Caudal Middle Frontal Gryus | MA : RES | 0.01 | [-0.19, 0.2] | 1.01 | [0.83, 1.22] | 0.971 |
|  | VU : RES | 0.02 | [-0.16, 0.2] | 1.02 | [0.85, 1.23] | 0.889 |
|  | HC : RES | 0.03 | [-0.03, 0.09] | 1.03 | [0.97, 1.1] | 0.515 |
| Cuneus | MA : RES | -0.11 | [-0.3, 0.08] | 0.89 | [0.74, 1.08] | 0.647 |
|  | VU : RES | 0.15 | [-0.03, 0.33] | 1.16 | [0.97, 1.39] | 0.571 |
|  | HC : RES | 0.04 | [-0.02, 0.1] | 1.04 | [0.98, 1.1] | 0.515 |
| Entorhinal Cortex | MA : RES | 0.21 | [0.02, 0.41] | 1.24 | [1.02, 1.5] | 0.489 |
|  | VU : RES | 0.05 | [-0.14, 0.24] | 1.05 | [0.87, 1.27] | 0.784 |
|  | HC : RES | 0.03 | [-0.03, 0.09] | 1.03 | [0.97, 1.1] | 0.520 |
| Frontal Pole | MA : RES | -0.04 | [-0.24, 0.15] | 0.96 | [0.79, 1.16] | 0.827 |
|  | VU : RES | 0.06 | [-0.12, 0.25] | 1.07 | [0.89, 1.28] | 0.709 |
|  | HC : RES | -0.01 | [-0.07, 0.05] | 0.99 | [0.93, 1.05] | 0.758 |
| Fusiform Area | MA : RES | -0.19 | [-0.38, -0.01] | 0.82 | [0.68, 0.99] | 0.489 |
|  | VU : RES | 0.02 | [-0.17, 0.2] | 1.02 | [0.85, 1.22] | 0.889 |
|  | HC : RES | 0.05 | [-0.01, 0.11] | 1.05 | [0.99, 1.11] | 0.382 |
| Inferior Parietal Lobule | MA : RES | -0.02 | [-0.22, 0.18] | 0.98 | [0.8, 1.19] | 0.905 |
|  | VU : RES | 0.10 | [-0.09, 0.29] | 1.11 | [0.91, 1.34] | 0.630 |
|  | HC : RES | 0.06 | [0, 0.12] | 1.06 | [1, 1.13] | 0.331 |
| Inferior Temporal Cortex | MA : RES | -0.03 | [-0.22, 0.16] | 0.97 | [0.8, 1.17] | 0.876 |
|  | VU : RES | 0.12 | [-0.07, 0.3] | 1.12 | [0.94, 1.35] | 0.571 |
|  | HC : RES | 0.02 | [-0.04, 0.08] | 1.02 | [0.96, 1.09] | 0.621 |
| Isthmus Cingulate Gyrus | MA : RES | -0.11 | [-0.3, 0.08] | 0.89 | [0.74, 1.08] | 0.647 |
|  | VU : RES | 0.06 | [-0.12, 0.24] | 1.06 | [0.89, 1.27] | 0.709 |
|  | HC : RES | -0.05 | [-0.11, 0.01] | 0.95 | [0.9, 1.01] | 0.343 |
| Insula | MA : RES | -0.05 | [-0.24, 0.15] | 0.96 | [0.79, 1.16] | 0.827 |
|  | VU : RES | 0.07 | [-0.12, 0.25] | 1.07 | [0.89, 1.29] | 0.709 |
|  | HC : RES | 0.01 | [-0.05, 0.07] | 1.01 | [0.95, 1.07] | 0.832 |
| Lingual Gyrus | MA : RES | -0.14 | [-0.33, 0.05] | 0.87 | [0.72, 1.06] | 0.647 |
|  | VU : RES | 0.17 | [-0.02, 0.36] | 1.18 | [0.98, 1.43] | 0.571 |
|  | HC : RES | 0.05 | [-0.02, 0.11] | 1.05 | [0.98, 1.11] | 0.382 |
| Lateral Orbitofrontal Cortex | MA : RES | -0.17 | [-0.36, 0.02] | 0.85 | [0.7, 1.02] | 0.647 |
|  | VU : RES | 0.10 | [-0.09, 0.28] | 1.10 | [0.92, 1.33] | 0.630 |
|  | HC : RES | 0.03 | [-0.03, 0.09] | 1.03 | [0.97, 1.09] | 0.582 |
| Lateral Occipital Cortex | MA : RES | -0.24 | [-0.45, -0.03] | 0.79 | [0.64, 0.98] | 0.489 |
|  | VU : RES | 0.10 | [-0.11, 0.3] | 1.10 | [0.9, 1.35] | 0.639 |
|  | HC : RES | 0.08 | [0.01, 0.15] | 1.08 | [1.01, 1.16] | 0.208 |
| Middle Temporal Gyrus | MA : RES | 0.08 | [-0.13, 0.28] | 1.08 | [0.88, 1.33] | 0.688 |
|  | VU : RES | 0.15 | [-0.05, 0.34] | 1.16 | [0.95, 1.41] | 0.571 |
|  | HC : RES | 0.06 | [-0.01, 0.12] | 1.06 | [0.99, 1.13] | 0.343 |
| Medial Orbitofrontal Cortex | MA : RES | -0.11 | [-0.3, 0.08] | 0.89 | [0.74, 1.08] | 0.647 |
|  | VU : RES | -0.01 | [-0.19, 0.18] | 0.99 | [0.83, 1.19] | 0.939 |
|  | HC : RES | 0.01 | [-0.05, 0.07] | 1.01 | [0.95, 1.07] | 0.770 |
| Paracentral Lobule | MA : RES | -0.10 | [-0.29, 0.09] | 0.90 | [0.75, 1.09] | 0.647 |
|  | VU : RES | 0.04 | [-0.14, 0.23] | 1.05 | [0.87, 1.26] | 0.787 |
|  | HC : RES | 0.08 | [0.02, 0.14] | 1.08 | [1.02, 1.15] | 0.199 |
| Parahippocampal Gyrus | MA : RES | -0.09 | [-0.28, 0.1] | 0.92 | [0.76, 1.11] | 0.647 |
|  | VU : RES | -0.04 | [-0.23, 0.14] | 0.96 | [0.8, 1.15] | 0.787 |
|  | HC : RES | -0.01 | [-0.07, 0.05] | 0.99 | [0.93, 1.05] | 0.758 |
| Pars Orbitalis | MA : RES | 0.09 | [-0.1, 0.28] | 1.09 | [0.9, 1.32] | 0.647 |
|  | VU : RES | 0.15 | [-0.04, 0.33] | 1.16 | [0.96, 1.39] | 0.571 |
|  | HC : RES | 0.02 | [-0.04, 0.08] | 1.02 | [0.96, 1.08] | 0.725 |
| Pars Opercularis | MA : RES | 0.09 | [-0.1, 0.28] | 1.09 | [0.9, 1.33] | 0.647 |
|  | VU : RES | 0.12 | [-0.07, 0.3] | 1.12 | [0.93, 1.35] | 0.571 |
|  | HC : RES | -0.02 | [-0.08, 0.04] | 0.98 | [0.92, 1.04] | 0.622 |
| Pars Triangularis | MA : RES | 0.09 | [-0.1, 0.28] | 1.09 | [0.9, 1.32] | 0.647 |
|  | VU : RES | 0.03 | [-0.15, 0.22] | 1.03 | [0.86, 1.24] | 0.806 |
|  | HC : RES | 0.04 | [-0.02, 0.1] | 1.04 | [0.98, 1.11] | 0.395 |
| Precuneus | MA : RES | -0.10 | [-0.28, 0.09] | 0.91 | [0.75, 1.1] | 0.647 |
|  | VU : RES | 0.09 | [-0.09, 0.28] | 1.10 | [0.91, 1.32] | 0.639 |
|  | HC : RES | 0.03 | [-0.03, 0.09] | 1.03 | [0.97, 1.09] | 0.580 |
| Pericalcarine Cortex | MA : RES | -0.11 | [-0.31, 0.08] | 0.89 | [0.73, 1.08] | 0.647 |
|  | VU : RES | 0.17 | [-0.02, 0.35] | 1.18 | [0.98, 1.43] | 0.571 |
|  | HC : RES | 0.03 | [-0.03, 0.1] | 1.03 | [0.97, 1.1] | 0.515 |
| Postcentral Gyrus | MA : RES | -0.14 | [-0.34, 0.06] | 0.87 | [0.71, 1.07] | 0.647 |
|  | VU : RES | 0.12 | [-0.06, 0.31] | 1.13 | [0.94, 1.37] | 0.571 |
|  | HC : RES | 0.09 | [0.03, 0.15] | 1.09 | [1.03, 1.17] | 0.158 |
| Precentral Gyrus | MA : RES | -0.02 | [-0.21, 0.17] | 0.98 | [0.81, 1.19] | 0.905 |
|  | VU : RES | -0.11 | [-0.28, 0.07] | 0.90 | [0.75, 1.07] | 0.571 |
|  | HC : RES | 0.03 | [-0.03, 0.09] | 1.03 | [0.97, 1.1] | 0.520 |
| Posterior Cingulate Cortex | MA : RES | 0.12 | [-0.07, 0.31] | 1.13 | [0.93, 1.36] | 0.647 |
|  | VU : RES | 0.24 | [0.06, 0.42] | 1.28 | [1.07, 1.53] | 0.276 |
|  | HC : RES | 0.03 | [-0.03, 0.09] | 1.03 | [0.97, 1.09] | 0.580 |
| Rostral Anterior Cingulate Cortex | MA : RES | 0.04 | [-0.15, 0.24] | 1.05 | [0.86, 1.27] | 0.827 |
|  | VU : RES | -0.07 | [-0.26, 0.11] | 0.93 | [0.77, 1.12] | 0.709 |
|  | HC : RES | -0.02 | [-0.08, 0.04] | 0.98 | [0.92, 1.04] | 0.725 |
| Rostral Middle Frontal Gyrus | MA : RES | 0.06 | [-0.13, 0.25] | 1.07 | [0.88, 1.29] | 0.735 |
|  | VU : RES | 0.07 | [-0.11, 0.25] | 1.07 | [0.89, 1.28] | 0.709 |
|  | HC : RES | 0.04 | [-0.02, 0.1] | 1.04 | [0.98, 1.1] | 0.445 |
| Supramarginal Gyrus | MA : RES | 0.08 | [-0.13, 0.29] | 1.08 | [0.88, 1.33] | 0.688 |
|  | VU : RES | 0.04 | [-0.15, 0.23] | 1.04 | [0.86, 1.26] | 0.806 |
|  | HC : RES | 0.06 | [-0.01, 0.12] | 1.06 | [0.99, 1.13] | 0.343 |
| Superior Frontal Gyrus | MA : RES | 0.12 | [-0.07, 0.31] | 1.12 | [0.93, 1.36] | 0.647 |
|  | VU : RES | 0.18 | [-0.01, 0.36] | 1.19 | [0.99, 1.43] | 0.571 |
|  | HC : RES | 0.07 | [0.01, 0.13] | 1.07 | [1.01, 1.14] | 0.208 |
| Superior Parietal Lobule | MA : RES | -0.09 | [-0.28, 0.11] | 0.92 | [0.76, 1.11] | 0.647 |
|  | VU : RES | 0.11 | [-0.07, 0.3] | 1.12 | [0.93, 1.35] | 0.571 |
|  | HC : RES | 0.05 | [-0.01, 0.11] | 1.05 | [0.99, 1.12] | 0.382 |
| Superior Temporal Gyrus | MA : RES | -0.02 | [-0.21, 0.17] | 0.98 | [0.81, 1.19] | 0.905 |
|  | VU : RES | 0.11 | [-0.07, 0.3] | 1.12 | [0.93, 1.35] | 0.571 |
|  | HC : RES | 0.05 | [-0.01, 0.11] | 1.05 | [0.99, 1.11] | 0.382 |
| Temporal Pole | MA : RES | 0.00 | [-0.19, 0.19] | 1.00 | [0.83, 1.21] | 0.986 |
|  | VU : RES | -0.02 | [-0.2, 0.17] | 0.98 | [0.82, 1.18] | 0.889 |
|  | HC : RES | 0.02 | [-0.04, 0.08] | 1.02 | [0.96, 1.08] | 0.700 |
| Transverse Temporal Gyrus | MA : RES | -0.03 | [-0.22, 0.16] | 0.97 | [0.8, 1.17] | 0.876 |
|  | VU : RES | 0.06 | [-0.12, 0.24] | 1.06 | [0.88, 1.28] | 0.710 |
|  | HC : RES | 0.00 | [-0.06, 0.06] | 1.00 | [0.94, 1.06] | 0.973 |
| *Subcortical volume* |  |  |  |  |  |  |
| Accumbens | MA : RES | 0.06 | [-0.19, 0.31] | 1.06 | [0.83, 1.37] | 0.981 |
|  | VU : RES | 0.19 | [-0.05, 0.42] | 1.20 | [0.95, 1.52] | 0.293 |
|  | HC : RES | 0.05 | [-0.03, 0.12] | 1.05 | [0.97, 1.13] | 0.960 |
| Amygdala | MA : RES | -0.02 | [-0.28, 0.25] | 0.98 | [0.75, 1.29] | 0.981 |
|  | VU : RES | -0.12 | [-0.37, 0.14] | 0.89 | [0.69, 1.16] | 0.483 |
|  | HC : RES | -0.03 | [-0.11, 0.05] | 0.97 | [0.89, 1.05] | 0.985 |
| Caudate | MA : RES | 0.12 | [-0.11, 0.35] | 1.13 | [0.9, 1.42] | 0.981 |
|  | VU : RES | 0.08 | [-0.14, 0.3] | 1.08 | [0.87, 1.35] | 0.483 |
|  | HC : RES | 0.00 | [-0.07, 0.07] | 1.00 | [0.93, 1.07] | 0.985 |
| Hippocampus | MA : RES | -0.25 | [-0.53, 0.02] | 0.78 | [0.59, 1.02] | 0.463 |
|  | VU : RES | 0.10 | [-0.15, 0.35] | 1.11 | [0.86, 1.43] | 0.483 |
|  | HC : RES | 0.01 | [-0.08, 0.09] | 1.01 | [0.93, 1.09] | 0.985 |
| Pallidum | MA : RES | 0.05 | [-0.19, 0.29] | 1.05 | [0.83, 1.33] | 0.981 |
|  | VU : RES | 0.12 | [-0.11, 0.35] | 1.13 | [0.9, 1.41] | 0.483 |
|  | HC : RES | 0.02 | [-0.06, 0.09] | 1.02 | [0.94, 1.1] | 0.985 |
| Putamen | MA : RES | -0.05 | [-0.28, 0.19] | 0.96 | [0.76, 1.21] | 0.981 |
|  | VU : RES | 0.31 | [0.09, 0.53] | 1.36 | [1.09, 1.7] | **0.042** |
|  | HC : RES | 0.04 | [-0.03, 0.11] | 1.04 | [0.97, 1.12] | 0.960 |
| Thalamus | MA : RES | 0.00 | [-0.28, 0.28] | 1.00 | [0.75, 1.32] | 0.981 |
|  | VU : RES | 0.21 | [-0.06, 0.47] | 1.23 | [0.94, 1.6] | 0.293 |
|  | HC : RES | -0.01 | [-0.1, 0.07] | 0.99 | [0.91, 1.08] | 0.985 |

## Sex interactions effects

### Table S3. Internalizing group comparisons

| **Variables** | **Group comparisons** | **B** | **95% CI** | **Odds** | **95% CI** | ***p*FDR** |
| --- | --- | --- | --- | --- | --- | --- |
| *Surface area* |  |  |  |  |  |  |
| Banks of Superior Temporal Sulcus | MA : RES | -0.38 | [-0.66, -0.1] | 0.68 | [0.52, 0.9] | **0.040** |
|  | VU : RES | -0.06 | [-0.31, 0.19] | 0.94 | [0.74, 1.2] | 0.856 |
|  | HC : RES | -0.10 | [-0.25, 0.06] | 0.91 | [0.78, 1.06] | 0.469 |
| Caudal Anterior Cingulate Cortex | MA : RES | -0.25 | [-0.53, 0.03] | 0.78 | [0.59, 1.03] | 0.122 |
|  | VU : RES | 0.03 | [-0.21, 0.27] | 1.03 | [0.81, 1.31] | 0.941 |
|  | HC : RES | -0.07 | [-0.22, 0.08] | 0.93 | [0.8, 1.09] | 0.570 |
| Caudal Middle Frontal Gryus | MA : RES | -0.05 | [-0.33, 0.22] | 0.95 | [0.72, 1.25] | 0.739 |
|  | VU : RES | 0.03 | [-0.21, 0.27] | 1.03 | [0.81, 1.31] | 0.941 |
|  | HC : RES | 0.07 | [-0.08, 0.22] | 1.07 | [0.92, 1.25] | 0.570 |
| Cuneus | MA : RES | -0.15 | [-0.43, 0.13] | 0.86 | [0.65, 1.13] | 0.320 |
|  | VU : RES | 0.10 | [-0.14, 0.34] | 1.11 | [0.87, 1.41] | 0.830 |
|  | HC : RES | 0.06 | [-0.09, 0.22] | 1.07 | [0.91, 1.24] | 0.584 |
| Entorhinal Cortex | MA : RES | -0.09 | [-0.37, 0.19] | 0.91 | [0.69, 1.21] | 0.565 |
|  | VU : RES | -0.10 | [-0.34, 0.14] | 0.91 | [0.72, 1.15] | 0.830 |
|  | HC : RES | -0.08 | [-0.23, 0.07] | 0.92 | [0.79, 1.08] | 0.570 |
| Frontal Pole | MA : RES | -0.38 | [-0.67, -0.09] | 0.68 | [0.51, 0.91] | **0.040** |
|  | VU : RES | -0.12 | [-0.37, 0.13] | 0.89 | [0.69, 1.14] | 0.830 |
|  | HC : RES | -0.10 | [-0.26, 0.06] | 0.90 | [0.77, 1.06] | 0.469 |
| Fusiform Area | MA : RES | -0.52 | [-0.82, -0.23] | 0.59 | [0.44, 0.8] | **0.018** |
|  | VU : RES | -0.18 | [-0.43, 0.08] | 0.84 | [0.65, 1.08] | 0.830 |
|  | HC : RES | -0.17 | [-0.33, -0.01] | 0.84 | [0.72, 0.99] | **0.336** |
| Inferior Parietal Lobule | MA : RES | -0.39 | [-0.67, -0.11] | 0.68 | [0.51, 0.9] | **0.040** |
|  | VU : RES | -0.08 | [-0.33, 0.16] | 0.92 | [0.72, 1.17] | 0.830 |
|  | HC : RES | -0.18 | [-0.34, -0.03] | 0.83 | [0.71, 0.97] | **0.336** |
| Inferior Temporal Cortex | MA : RES | -0.32 | [-0.6, -0.04] | 0.72 | [0.55, 0.96] | **0.059** |
|  | VU : RES | -0.09 | [-0.33, 0.16] | 0.92 | [0.72, 1.17] | 0.830 |
|  | HC : RES | -0.12 | [-0.28, 0.04] | 0.89 | [0.76, 1.04] | 0.407 |
| Isthmus Cingulate Gyrus | MA : RES | -0.17 | [-0.46, 0.11] | 0.84 | [0.63, 1.12] | 0.277 |
|  | VU : RES | -0.21 | [-0.46, 0.04] | 0.81 | [0.63, 1.04] | 0.713 |
|  | HC : RES | -0.13 | [-0.29, 0.02] | 0.87 | [0.75, 1.02] | 0.363 |
| Insula | MA : RES | -0.19 | [-0.48, 0.1] | 0.83 | [0.62, 1.11] | 0.267 |
|  | VU : RES | -0.27 | [-0.52, -0.01] | 0.77 | [0.59, 0.99] | 0.648 |
|  | HC : RES | -0.16 | [-0.32, 0] | 0.86 | [0.73, 1] | 0.360 |
| Lingual Gyrus | MA : RES | -0.02 | [-0.29, 0.25] | 0.98 | [0.74, 1.29] | 0.881 |
|  | VU : RES | -0.04 | [-0.28, 0.2] | 0.96 | [0.76, 1.23] | 0.941 |
|  | HC : RES | -0.01 | [-0.16, 0.14] | 0.99 | [0.85, 1.15] | 0.961 |
| Lateral Orbitofrontal Cortex | MA : RES | -0.19 | [-0.48, 0.09] | 0.82 | [0.62, 1.1] | 0.249 |
|  | VU : RES | 0.14 | [-0.11, 0.39] | 1.15 | [0.9, 1.47] | 0.830 |
|  | HC : RES | -0.03 | [-0.18, 0.13] | 0.97 | [0.83, 1.14] | 0.860 |
| Lateral Occipital Cortex | MA : RES | -0.28 | [-0.57, 0.01] | 0.75 | [0.56, 1.01] | 0.105 |
|  | VU : RES | -0.03 | [-0.28, 0.23] | 0.97 | [0.76, 1.25] | 0.948 |
|  | HC : RES | -0.04 | [-0.21, 0.12] | 0.96 | [0.81, 1.12] | 0.800 |
| Middle Temporal Gyrus | MA : RES | -0.30 | [-0.58, -0.01] | 0.74 | [0.56, 0.99] | 0.077 |
|  | VU : RES | -0.12 | [-0.37, 0.13] | 0.89 | [0.69, 1.14] | 0.830 |
|  | HC : RES | -0.12 | [-0.28, 0.03] | 0.89 | [0.76, 1.04] | 0.407 |
| Medial Orbitofrontal Cortex | MA : RES | -0.38 | [-0.67, -0.09] | 0.68 | [0.51, 0.91] | **0.040** |
|  | VU : RES | -0.01 | [-0.26, 0.24] | 0.99 | [0.77, 1.27] | 0.989 |
|  | HC : RES | -0.14 | [-0.3, 0.02] | 0.87 | [0.74, 1.02] | 0.363 |
| Paracentral Lobule | MA : RES | -0.40 | [-0.68, -0.11] | 0.67 | [0.51, 0.9] | **0.040** |
|  | VU : RES | -0.08 | [-0.33, 0.16] | 0.92 | [0.72, 1.17] | 0.830 |
|  | HC : RES | -0.11 | [-0.26, 0.05] | 0.90 | [0.77, 1.05] | 0.438 |
| Parahippocampal Gyrus | MA : RES | -0.26 | [-0.54, 0.02] | 0.77 | [0.58, 1.02] | 0.118 |
|  | VU : RES | -0.23 | [-0.48, 0.01] | 0.79 | [0.62, 1.01] | 0.648 |
|  | HC : RES | -0.15 | [-0.31, 0.01] | 0.86 | [0.74, 1.01] | 0.360 |
| Pars Orbitalis | MA : RES | -0.38 | [-0.68, -0.08] | 0.68 | [0.51, 0.92] | **0.040** |
|  | VU : RES | -0.08 | [-0.34, 0.17] | 0.92 | [0.71, 1.19] | 0.830 |
|  | HC : RES | 0.00 | [-0.16, 0.17] | 1.00 | [0.85, 1.18] | 0.971 |
| Pars Opercularis | MA : RES | -0.15 | [-0.42, 0.12] | 0.86 | [0.66, 1.13] | 0.320 |
|  | VU : RES | -0.11 | [-0.35, 0.13] | 0.90 | [0.71, 1.14] | 0.830 |
|  | HC : RES | -0.07 | [-0.22, 0.08] | 0.93 | [0.8, 1.09] | 0.570 |
| Pars Triangularis | MA : RES | -0.26 | [-0.54, 0.03] | 0.77 | [0.58, 1.03] | 0.122 |
|  | VU : RES | -0.29 | [-0.54, -0.04] | 0.75 | [0.58, 0.96] | 0.648 |
|  | HC : RES | -0.08 | [-0.24, 0.08] | 0.92 | [0.79, 1.08] | 0.570 |
| Precuneus | MA : RES | -0.31 | [-0.6, -0.02] | 0.73 | [0.55, 0.98] | 0.071 |
|  | VU : RES | -0.10 | [-0.35, 0.14] | 0.90 | [0.71, 1.15] | 0.830 |
|  | HC : RES | -0.12 | [-0.27, 0.04] | 0.89 | [0.76, 1.04] | 0.425 |
| Pericalcarine Cortex | MA : RES | -0.08 | [-0.35, 0.18] | 0.92 | [0.7, 1.2] | 0.570 |
|  | VU : RES | 0.08 | [-0.15, 0.32] | 1.09 | [0.86, 1.38] | 0.830 |
|  | HC : RES | -0.01 | [-0.16, 0.13] | 0.99 | [0.85, 1.14] | 0.936 |
| Postcentral Gyrus | MA : RES | -0.34 | [-0.62, -0.05] | 0.71 | [0.54, 0.95] | 0.056 |
|  | VU : RES | -0.03 | [-0.29, 0.22] | 0.97 | [0.75, 1.24] | 0.941 |
|  | HC : RES | -0.04 | [-0.2, 0.12] | 0.96 | [0.82, 1.13] | 0.809 |
| Precentral Gyrus | MA : RES | -0.38 | [-0.67, -0.09] | 0.68 | [0.51, 0.92] | **0.040** |
|  | VU : RES | 0.01 | [-0.25, 0.26] | 1.01 | [0.78, 1.3] | 0.989 |
|  | HC : RES | -0.02 | [-0.18, 0.14] | 0.98 | [0.84, 1.15] | 0.936 |
| Posterior Cingulate Cortex | MA : RES | -0.35 | [-0.64, -0.06] | 0.70 | [0.53, 0.94] | **0.050** |
|  | VU : RES | -0.08 | [-0.33, 0.17] | 0.92 | [0.72, 1.18] | 0.830 |
|  | HC : RES | -0.17 | [-0.33, -0.02] | 0.84 | [0.72, 0.98] | 0.336 |
| Rostral Anterior Cingulate Cortex | MA : RES | -0.36 | [-0.63, -0.08] | 0.70 | [0.53, 0.92] | **0.040** |
|  | VU : RES | 0.02 | [-0.23, 0.26] | 1.02 | [0.8, 1.3] | 0.982 |
|  | HC : RES | -0.10 | [-0.25, 0.05] | 0.90 | [0.78, 1.06] | 0.469 |
| Rostral Middle Frontal Gyrus | MA : RES | -0.33 | [-0.62, -0.04] | 0.72 | [0.54, 0.96] | 0.059 |
|  | VU : RES | -0.07 | [-0.32, 0.18] | 0.93 | [0.72, 1.19] | 0.834 |
|  | HC : RES | -0.08 | [-0.24, 0.08] | 0.93 | [0.79, 1.08] | 0.570 |
| Supramarginal Gyrus | MA : RES | -0.38 | [-0.67, -0.08] | 0.69 | [0.51, 0.92] | **0.040** |
|  | VU : RES | 0.00 | [-0.25, 0.25] | 1.00 | [0.78, 1.28] | 0.991 |
|  | HC : RES | -0.03 | [-0.19, 0.13] | 0.97 | [0.83, 1.13] | 0.860 |
| Superior Frontal Gyrus | MA : RES | -0.36 | [-0.65, -0.07] | 0.70 | [0.52, 0.94] | **0.050** |
|  | VU : RES | -0.20 | [-0.45, 0.06] | 0.82 | [0.64, 1.06] | 0.715 |
|  | HC : RES | -0.13 | [-0.29, 0.02] | 0.87 | [0.75, 1.02] | 0.363 |
| Superior Parietal Lobule | MA : RES | -0.17 | [-0.45, 0.1] | 0.84 | [0.64, 1.11] | 0.277 |
|  | VU : RES | 0.07 | [-0.17, 0.3] | 1.07 | [0.84, 1.35] | 0.834 |
|  | HC : RES | 0.00 | [-0.15, 0.15] | 1.00 | [0.86, 1.16] | 0.971 |
| Superior Temporal Gyrus | MA : RES | -0.30 | [-0.59, -0.01] | 0.74 | [0.55, 0.99] | 0.077 |
|  | VU : RES | -0.11 | [-0.36, 0.14] | 0.90 | [0.7, 1.15] | 0.830 |
|  | HC : RES | -0.07 | [-0.23, 0.09] | 0.93 | [0.8, 1.09] | 0.570 |
| Temporal Pole | MA : RES | -0.23 | [-0.53, 0.06] | 0.79 | [0.59, 1.06] | 0.170 |
|  | VU : RES | -0.23 | [-0.48, 0.02] | 0.80 | [0.62, 1.02] | 0.648 |
|  | HC : RES | -0.18 | [-0.34, -0.02] | 0.84 | [0.71, 0.98] | 0.336 |
| Transverse Temporal Gyrus | MA : RES | -0.22 | [-0.5, 0.06] | 0.80 | [0.61, 1.06] | 0.173 |
|  | VU : RES | -0.08 | [-0.32, 0.17] | 0.93 | [0.73, 1.18] | 0.830 |
|  | HC : RES | -0.03 | [-0.18, 0.13] | 0.97 | [0.83, 1.13] | 0.860 |
| *Cortical thickness* |  |  |  |  |  |  |
| Banks of Superior Temporal Sulcus | MA : RES | -0.09 | [-0.35, 0.17] | 0.92 | [0.71, 1.19] | 0.852 |
|  | VU : RES | 0.12 | [-0.11, 0.35] | 1.13 | [0.9, 1.42] | 0.905 |
|  | HC : RES | -0.04 | [-0.18, 0.11] | 0.97 | [0.84, 1.12] | 0.983 |
| Caudal Anterior Cingulate Cortex | MA : RES | -0.10 | [-0.37, 0.17] | 0.90 | [0.69, 1.18] | 0.852 |
|  | VU : RES | -0.04 | [-0.27, 0.19] | 0.96 | [0.76, 1.2] | 0.930 |
|  | HC : RES | 0.08 | [-0.07, 0.22] | 1.08 | [0.93, 1.25] | 0.983 |
| Caudal Middle Frontal Gryus | MA : RES | 0.00 | [-0.27, 0.26] | 1.00 | [0.76, 1.3] | 0.999 |
|  | VU : RES | 0.06 | [-0.18, 0.29] | 1.06 | [0.84, 1.34] | 0.930 |
|  | HC : RES | 0.08 | [-0.07, 0.22] | 1.08 | [0.93, 1.25] | 0.983 |
| Cuneus | MA : RES | -0.17 | [-0.43, 0.09] | 0.85 | [0.65, 1.1] | 0.852 |
|  | VU : RES | -0.02 | [-0.25, 0.21] | 0.98 | [0.78, 1.23] | 0.985 |
|  | HC : RES | -0.04 | [-0.18, 0.1] | 0.96 | [0.83, 1.11] | 0.983 |
| Entorhinal Cortex | MA : RES | -0.10 | [-0.37, 0.17] | 0.90 | [0.69, 1.18] | 0.852 |
|  | VU : RES | 0.04 | [-0.19, 0.28] | 1.04 | [0.83, 1.32] | 0.930 |
|  | HC : RES | 0.00 | [-0.15, 0.15] | 1.00 | [0.86, 1.16] | 0.998 |
| Frontal Pole | MA : RES | -0.08 | [-0.35, 0.18] | 0.92 | [0.71, 1.2] | 0.852 |
|  | VU : RES | -0.12 | [-0.35, 0.11] | 0.89 | [0.7, 1.12] | 0.905 |
|  | HC : RES | -0.09 | [-0.23, 0.06] | 0.91 | [0.79, 1.06] | 0.983 |
| Fusiform Area | MA : RES | -0.18 | [-0.44, 0.09] | 0.84 | [0.65, 1.09] | 0.852 |
|  | VU : RES | 0.04 | [-0.19, 0.27] | 1.04 | [0.83, 1.31] | 0.930 |
|  | HC : RES | -0.10 | [-0.24, 0.04] | 0.90 | [0.78, 1.05] | 0.983 |
| Inferior Parietal Lobule | MA : RES | 0.01 | [-0.26, 0.28] | 1.01 | [0.77, 1.32] | 0.999 |
|  | VU : RES | 0.10 | [-0.13, 0.34] | 1.11 | [0.88, 1.4] | 0.905 |
|  | HC : RES | 0.02 | [-0.13, 0.17] | 1.02 | [0.88, 1.18] | 0.983 |
| Inferior Temporal Cortex | MA : RES | -0.17 | [-0.44, 0.09] | 0.84 | [0.65, 1.1] | 0.852 |
|  | VU : RES | -0.08 | [-0.31, 0.16] | 0.93 | [0.73, 1.17] | 0.905 |
|  | HC : RES | -0.17 | [-0.31, -0.02] | 0.85 | [0.73, 0.98] | 0.930 |
| Isthmus Cingulate Gyrus | MA : RES | -0.09 | [-0.35, 0.17] | 0.91 | [0.7, 1.19] | 0.852 |
|  | VU : RES | -0.13 | [-0.36, 0.1] | 0.88 | [0.7, 1.1] | 0.905 |
|  | HC : RES | -0.04 | [-0.19, 0.1] | 0.96 | [0.83, 1.11] | 0.983 |
| Insula | MA : RES | -0.23 | [-0.5, 0.04] | 0.79 | [0.61, 1.04] | 0.852 |
|  | VU : RES | -0.14 | [-0.37, 0.1] | 0.87 | [0.69, 1.1] | 0.905 |
|  | HC : RES | -0.02 | [-0.16, 0.13] | 0.98 | [0.85, 1.14] | 0.983 |
| Lingual Gyrus | MA : RES | -0.22 | [-0.48, 0.05] | 0.81 | [0.62, 1.05] | 0.852 |
|  | VU : RES | -0.09 | [-0.32, 0.14] | 0.91 | [0.73, 1.15] | 0.905 |
|  | HC : RES | -0.08 | [-0.22, 0.07] | 0.93 | [0.8, 1.07] | 0.983 |
| Lateral Orbitofrontal Cortex | MA : RES | -0.21 | [-0.48, 0.06] | 0.81 | [0.62, 1.06] | 0.852 |
|  | VU : RES | 0.06 | [-0.17, 0.29] | 1.06 | [0.84, 1.34] | 0.930 |
|  | HC : RES | 0.00 | [-0.15, 0.14] | 1.00 | [0.86, 1.15] | 0.998 |
| Lateral Occipital Cortex | MA : RES | -0.09 | [-0.36, 0.17] | 0.91 | [0.7, 1.19] | 0.852 |
|  | VU : RES | -0.10 | [-0.33, 0.13] | 0.90 | [0.72, 1.14] | 0.905 |
|  | HC : RES | -0.03 | [-0.17, 0.12] | 0.97 | [0.84, 1.13] | 0.983 |
| Middle Temporal Gyrus | MA : RES | 0.03 | [-0.24, 0.29] | 1.03 | [0.79, 1.34] | 0.999 |
|  | VU : RES | 0.07 | [-0.16, 0.31] | 1.08 | [0.85, 1.36] | 0.905 |
|  | HC : RES | -0.02 | [-0.16, 0.13] | 0.98 | [0.85, 1.14] | 0.983 |
| Medial Orbitofrontal Cortex | MA : RES | -0.08 | [-0.35, 0.19] | 0.92 | [0.71, 1.2] | 0.852 |
|  | VU : RES | -0.23 | [-0.46, 0.01] | 0.80 | [0.63, 1.01] | 0.905 |
|  | HC : RES | -0.10 | [-0.25, 0.04] | 0.90 | [0.78, 1.04] | 0.983 |
| Paracentral Lobule | MA : RES | 0.10 | [-0.16, 0.37] | 1.11 | [0.85, 1.45] | 0.852 |
|  | VU : RES | 0.07 | [-0.16, 0.3] | 1.08 | [0.85, 1.35] | 0.905 |
|  | HC : RES | 0.05 | [-0.1, 0.19] | 1.05 | [0.91, 1.21] | 0.983 |
| Parahippocampal Gyrus | MA : RES | -0.15 | [-0.41, 0.12] | 0.86 | [0.66, 1.13] | 0.852 |
|  | VU : RES | 0.14 | [-0.1, 0.37] | 1.15 | [0.91, 1.45] | 0.905 |
|  | HC : RES | 0.01 | [-0.14, 0.15] | 1.01 | [0.87, 1.16] | 0.998 |
| Pars Orbitalis | MA : RES | -0.04 | [-0.3, 0.23] | 0.96 | [0.74, 1.26] | 0.999 |
|  | VU : RES | 0.11 | [-0.12, 0.35] | 1.12 | [0.89, 1.41] | 0.905 |
|  | HC : RES | -0.08 | [-0.23, 0.07] | 0.92 | [0.8, 1.07] | 0.983 |
| Pars Opercularis | MA : RES | -0.09 | [-0.35, 0.18] | 0.92 | [0.7, 1.2] | 0.852 |
|  | VU : RES | -0.10 | [-0.33, 0.13] | 0.90 | [0.72, 1.14] | 0.905 |
|  | HC : RES | -0.07 | [-0.21, 0.08] | 0.93 | [0.81, 1.08] | 0.983 |
| Pars Triangularis | MA : RES | -0.02 | [-0.29, 0.24] | 0.98 | [0.75, 1.28] | 0.999 |
|  | VU : RES | -0.01 | [-0.25, 0.22] | 0.99 | [0.78, 1.24] | 0.985 |
|  | HC : RES | -0.08 | [-0.23, 0.07] | 0.92 | [0.8, 1.07] | 0.983 |
| Precuneus | MA : RES | 0.13 | [-0.14, 0.4] | 1.14 | [0.87, 1.49] | 0.852 |
|  | VU : RES | 0.11 | [-0.12, 0.35] | 1.12 | [0.88, 1.41] | 0.905 |
|  | HC : RES | 0.06 | [-0.09, 0.21] | 1.06 | [0.92, 1.23] | 0.983 |
| Pericalcarine Cortex | MA : RES | -0.18 | [-0.44, 0.09] | 0.84 | [0.65, 1.09] | 0.852 |
|  | VU : RES | -0.08 | [-0.31, 0.15] | 0.92 | [0.73, 1.16] | 0.905 |
|  | HC : RES | -0.05 | [-0.19, 0.1] | 0.95 | [0.82, 1.1] | 0.983 |
| Postcentral Gyrus | MA : RES | 0.03 | [-0.24, 0.29] | 1.03 | [0.79, 1.34] | 0.999 |
|  | VU : RES | -0.18 | [-0.41, 0.05] | 0.83 | [0.66, 1.05] | 0.905 |
|  | HC : RES | -0.04 | [-0.19, 0.11] | 0.96 | [0.83, 1.11] | 0.983 |
| Precentral Gyrus | MA : RES | -0.03 | [-0.29, 0.23] | 0.97 | [0.75, 1.25] | 0.999 |
|  | VU : RES | 0.00 | [-0.22, 0.23] | 1.00 | [0.8, 1.26] | 0.985 |
|  | HC : RES | -0.01 | [-0.15, 0.13] | 0.99 | [0.86, 1.14] | 0.998 |
| Posterior Cingulate Cortex | MA : RES | 0.00 | [-0.27, 0.26] | 1.00 | [0.76, 1.3] | 0.999 |
|  | VU : RES | -0.01 | [-0.24, 0.22] | 0.99 | [0.79, 1.24] | 0.985 |
|  | HC : RES | 0.00 | [-0.15, 0.14] | 1.00 | [0.86, 1.15] | 0.998 |
| Rostral Anterior Cingulate Cortex | MA : RES | 0.01 | [-0.25, 0.28] | 1.01 | [0.78, 1.32] | 0.999 |
|  | VU : RES | -0.12 | [-0.35, 0.11] | 0.89 | [0.71, 1.12] | 0.905 |
|  | HC : RES | -0.02 | [-0.16, 0.13] | 0.98 | [0.85, 1.14] | 0.983 |
| Rostral Middle Frontal Gyrus | MA : RES | -0.06 | [-0.32, 0.21] | 0.94 | [0.73, 1.23] | 0.946 |
|  | VU : RES | -0.01 | [-0.24, 0.22] | 0.99 | [0.79, 1.25] | 0.985 |
|  | HC : RES | -0.06 | [-0.21, 0.08] | 0.94 | [0.81, 1.08] | 0.983 |
| Supramarginal Gyrus | MA : RES | 0.11 | [-0.16, 0.38] | 1.12 | [0.85, 1.46] | 0.852 |
|  | VU : RES | 0.05 | [-0.19, 0.28] | 1.05 | [0.83, 1.32] | 0.930 |
|  | HC : RES | 0.02 | [-0.13, 0.17] | 1.02 | [0.88, 1.18] | 0.983 |
| Superior Frontal Gyrus | MA : RES | 0.06 | [-0.21, 0.33] | 1.06 | [0.81, 1.39] | 0.946 |
|  | VU : RES | 0.14 | [-0.1, 0.37] | 1.15 | [0.91, 1.45] | 0.905 |
|  | HC : RES | 0.04 | [-0.1, 0.19] | 1.05 | [0.9, 1.21] | 0.983 |
| Superior Parietal Lobule | MA : RES | 0.21 | [-0.06, 0.48] | 1.23 | [0.95, 1.61] | 0.852 |
|  | VU : RES | 0.04 | [-0.19, 0.28] | 1.04 | [0.83, 1.32] | 0.930 |
|  | HC : RES | 0.03 | [-0.12, 0.18] | 1.03 | [0.89, 1.19] | 0.983 |
| Superior Temporal Gyrus | MA : RES | 0.12 | [-0.15, 0.38] | 1.12 | [0.86, 1.47] | 0.852 |
|  | VU : RES | 0.00 | [-0.23, 0.24] | 1.00 | [0.79, 1.27] | 0.985 |
|  | HC : RES | -0.03 | [-0.18, 0.11] | 0.97 | [0.84, 1.12] | 0.983 |
| Temporal Pole | MA : RES | 0.00 | [-0.27, 0.27] | 1.00 | [0.76, 1.31] | 0.999 |
|  | VU : RES | 0.10 | [-0.13, 0.33] | 1.10 | [0.88, 1.39] | 0.905 |
|  | HC : RES | 0.08 | [-0.06, 0.23] | 1.09 | [0.94, 1.26] | 0.983 |
| Transverse Temporal Gyrus | MA : RES | 0.11 | [-0.15, 0.38] | 1.12 | [0.86, 1.46] | 0.852 |
|  | VU : RES | 0.02 | [-0.21, 0.25] | 1.02 | [0.81, 1.29] | 0.985 |
|  | HC : RES | -0.05 | [-0.19, 0.1] | 0.96 | [0.82, 1.11] | 0.983 |
| *Subcortical volume* |  |  |  |  |  |  |
| Accumbens | MA : RES | -0.39 | [-0.68, -0.11] | 0.67 | [0.51, 0.89] | **0.044** |
|  | VU : RES | -0.16 | [-0.41, 0.09] | 0.85 | [0.67, 1.09] | 0.235 |
|  | HC : RES | -0.13 | [-0.28, 0.03] | 0.88 | [0.75, 1.03] | 0.192 |
| Amygdala | MA : RES | -0.17 | [-0.45, 0.12] | 0.85 | [0.64, 1.13] | 0.354 |
|  | VU : RES | -0.41 | [-0.66, -0.16] | 0.66 | [0.52, 0.85] | **0.009** |
|  | HC : RES | -0.17 | [-0.33, -0.01] | 0.84 | [0.72, 0.99] | 0.088 |
| Caudate | MA : RES | -0.18 | [-0.45, 0.09] | 0.84 | [0.64, 1.1] | 0.349 |
|  | VU : RES | -0.18 | [-0.42, 0.06] | 0.83 | [0.66, 1.06] | 0.191 |
|  | HC : RES | -0.11 | [-0.26, 0.04] | 0.90 | [0.77, 1.04] | 0.218 |
| Hippocampus | MA : RES | -0.29 | [-0.57, 0] | 0.75 | [0.56, 1] | 0.166 |
|  | VU : RES | -0.21 | [-0.46, 0.03] | 0.81 | [0.63, 1.03] | 0.158 |
|  | HC : RES | -0.23 | [-0.38, -0.07] | 0.80 | [0.68, 0.93] | **0.033** |
| Pallidum | MA : RES | -0.09 | [-0.37, 0.2] | 0.92 | [0.69, 1.22] | 0.546 |
|  | VU : RES | -0.23 | [-0.48, 0.02] | 0.80 | [0.62, 1.02] | 0.158 |
|  | HC : RES | -0.08 | [-0.24, 0.07] | 0.92 | [0.79, 1.08] | 0.300 |
| Putamen | MA : RES | -0.21 | [-0.5, 0.08] | 0.81 | [0.61, 1.08] | 0.349 |
|  | VU : RES | -0.25 | [-0.5, 0.01] | 0.78 | [0.61, 1.01] | 0.158 |
|  | HC : RES | -0.17 | [-0.33, -0.01] | 0.84 | [0.72, 0.99] | 0.088 |
| Thalamus | MA : RES | -0.13 | [-0.43, 0.16] | 0.88 | [0.65, 1.18] | 0.443 |
|  | VU : RES | -0.07 | [-0.32, 0.19] | 0.94 | [0.72, 1.21] | 0.609 |
|  | HC : RES | -0.10 | [-0.26, 0.06] | 0.90 | [0.77, 1.06] | 0.251 |

### Table S4. Externalizing group comparisons

| **Variables** | **Group comparisons** | **B** | **95% CI** | **Odds** | **95% CI** | ***p*FDR** |
| --- | --- | --- | --- | --- | --- | --- |
| *Surface area* |  |  |  |  |  |  |
| Banks of Superior Temporal Sulcus | MA : RES | 0.03 | [-0.35, 0.4] | 1.03 | [0.7, 1.5] | 0.964 |
|  | VU : RES | -0.02 | [-0.4, 0.35] | 0.98 | [0.67, 1.42] | 0.964 |
|  | HC : RES | -0.06 | [-0.45, 0.32] | 0.94 | [0.64, 1.38] | 0.964 |
| Caudal Anterior Cingulate Cortex | MA : RES | -0.14 | [-0.51, 0.23] | 0.87 | [0.6, 1.26] | 0.964 |
|  | VU : RES | 0.07 | [-0.32, 0.46] | 1.07 | [0.73, 1.58] | 0.964 |
|  | HC : RES | -0.14 | [-0.5, 0.23] | 0.87 | [0.6, 1.26] | 0.964 |
| Caudal Middle Frontal Gryus | MA : RES | -0.04 | [-0.42, 0.33] | 0.96 | [0.66, 1.39] | 0.964 |
|  | VU : RES | -0.23 | [-0.6, 0.14] | 0.79 | [0.55, 1.15] | 0.964 |
|  | HC : RES | -0.01 | [-0.39, 0.37] | 0.99 | [0.68, 1.45] | 0.974 |
| Cuneus | MA : RES | -0.13 | [-0.5, 0.25] | 0.88 | [0.61, 1.28] | 0.964 |
|  | VU : RES | 0.15 | [-0.23, 0.53] | 1.16 | [0.8, 1.7] | 0.964 |
|  | HC : RES | -0.21 | [-0.58, 0.16] | 0.81 | [0.56, 1.17] | 0.964 |
| Entorhinal Cortex | MA : RES | -0.07 | [-0.44, 0.31] | 0.94 | [0.64, 1.36] | 0.964 |
|  | VU : RES | 0.06 | [-0.32, 0.44] | 1.06 | [0.73, 1.56] | 0.964 |
|  | HC : RES | -0.16 | [-0.53, 0.22] | 0.85 | [0.59, 1.25] | 0.964 |
| Frontal Pole | MA : RES | 0.08 | [-0.29, 0.46] | 1.09 | [0.75, 1.58] | 0.964 |
|  | VU : RES | -0.04 | [-0.41, 0.34] | 0.96 | [0.66, 1.4] | 0.964 |
|  | HC : RES | -0.21 | [-0.59, 0.17] | 0.81 | [0.56, 1.19] | 0.964 |
| Fusiform Area | MA : RES | -0.08 | [-0.46, 0.29] | 0.92 | [0.63, 1.34] | 0.964 |
|  | VU : RES | 0.09 | [-0.29, 0.46] | 1.09 | [0.75, 1.59] | 0.964 |
|  | HC : RES | 0.15 | [-0.24, 0.54] | 1.16 | [0.79, 1.71] | 0.964 |
| Inferior Parietal Lobule | MA : RES | -0.20 | [-0.58, 0.17] | 0.82 | [0.56, 1.19] | 0.964 |
|  | VU : RES | 0.07 | [-0.3, 0.44] | 1.07 | [0.74, 1.55] | 0.964 |
|  | HC : RES | -0.04 | [-0.41, 0.33] | 0.96 | [0.66, 1.39] | 0.964 |
| Inferior Temporal Cortex | MA : RES | 0.10 | [-0.28, 0.48] | 1.10 | [0.75, 1.62] | 0.964 |
|  | VU : RES | -0.14 | [-0.51, 0.24] | 0.87 | [0.6, 1.27] | 0.964 |
|  | HC : RES | -0.12 | [-0.5, 0.26] | 0.88 | [0.6, 1.3] | 0.964 |
| Isthmus Cingulate Gyrus | MA : RES | 0.05 | [-0.33, 0.42] | 1.05 | [0.72, 1.53] | 0.964 |
|  | VU : RES | 0.02 | [-0.35, 0.39] | 1.02 | [0.7, 1.48] | 0.964 |
|  | HC : RES | 0.24 | [-0.14, 0.63] | 1.27 | [0.87, 1.87] | 0.964 |
| Insula | MA : RES | -0.07 | [-0.44, 0.31] | 0.94 | [0.64, 1.37] | 0.964 |
|  | VU : RES | 0.01 | [-0.37, 0.39] | 1.01 | [0.69, 1.47] | 0.974 |
|  | HC : RES | 0.28 | [-0.1, 0.67] | 1.33 | [0.91, 1.94] | 0.964 |
| Lingual Gyrus | MA : RES | -0.20 | [-0.57, 0.18] | 0.82 | [0.56, 1.2] | 0.964 |
|  | VU : RES | 0.17 | [-0.2, 0.53] | 1.18 | [0.82, 1.7] | 0.692 |
|  | HC : RES | 0.14 | [-0.22, 0.5] | 1.15 | [0.8, 1.65] | 0.692 |
| Lateral Orbitofrontal Cortex | MA : RES | 0.12 | [-0.25, 0.49] | 1.13 | [0.78, 1.63] | 0.692 |
|  | VU : RES | 0.11 | [-0.25, 0.46] | 1.11 | [0.78, 1.59] | 0.696 |
|  | HC : RES | 0.44 | [0.07, 0.81] | 1.55 | [1.07, 2.24] | 0.342 |
| Lateral Occipital Cortex | MA : RES | 0.26 | [-0.1, 0.63] | 1.30 | [0.9, 1.88] | 0.692 |
|  | VU : RES | 0.15 | [-0.22, 0.51] | 1.16 | [0.81, 1.67] | 0.692 |
|  | HC : RES | 0.12 | [-0.24, 0.48] | 1.12 | [0.78, 1.61] | 0.692 |
| Middle Temporal Gyrus | MA : RES | -0.26 | [-0.62, 0.1] | 0.77 | [0.54, 1.1] | 0.692 |
|  | VU : RES | 0.10 | [-0.26, 0.46] | 1.10 | [0.77, 1.58] | 0.696 |
|  | HC : RES | 0.38 | [0.01, 0.74] | 1.46 | [1.01, 2.1] | 0.492 |
| Medial Orbitofrontal Cortex | MA : RES | 0.12 | [-0.24, 0.49] | 1.13 | [0.78, 1.63] | 0.692 |
|  | VU : RES | 0.15 | [-0.2, 0.51] | 1.17 | [0.82, 1.67] | 0.692 |
|  | HC : RES | 0.15 | [-0.21, 0.52] | 1.16 | [0.81, 1.68] | 0.692 |
| Paracentral Lobule | MA : RES | -0.13 | [-0.5, 0.23] | 0.88 | [0.61, 1.26] | 0.692 |
|  | VU : RES | 0.03 | [-0.33, 0.4] | 1.03 | [0.72, 1.49] | 0.884 |
|  | HC : RES | -0.18 | [-0.54, 0.19] | 0.84 | [0.58, 1.2] | 0.692 |
| Parahippocampal Gyrus | MA : RES | -0.05 | [-0.42, 0.32] | 0.95 | [0.66, 1.38] | 0.873 |
|  | VU : RES | -0.22 | [-0.58, 0.14] | 0.80 | [0.56, 1.15] | 0.692 |
|  | HC : RES | 0.26 | [-0.1, 0.62] | 1.30 | [0.9, 1.86] | 0.692 |
| Pars Orbitalis | MA : RES | 0.09 | [-0.27, 0.45] | 1.10 | [0.77, 1.57] | 0.696 |
|  | VU : RES | -0.15 | [-0.51, 0.2] | 0.86 | [0.6, 1.23] | 0.692 |
|  | HC : RES | 0.03 | [-0.31, 0.37] | 1.03 | [0.73, 1.45] | 0.884 |
| Pars Opercularis | MA : RES | 0.24 | [-0.12, 0.6] | 1.27 | [0.89, 1.83] | 0.692 |
|  | VU : RES | 0.10 | [-0.27, 0.46] | 1.10 | [0.76, 1.59] | 0.696 |
|  | HC : RES | 0.03 | [-0.33, 0.39] | 1.03 | [0.72, 1.47] | 0.884 |
| Pars Triangularis | MA : RES | 0.18 | [-0.18, 0.55] | 1.20 | [0.83, 1.73] | 0.692 |
|  | VU : RES | 0.19 | [-0.18, 0.55] | 1.21 | [0.84, 1.74] | 0.692 |
|  | HC : RES | 0.19 | [-0.17, 0.55] | 1.21 | [0.85, 1.74] | 0.692 |
| Precuneus | MA : RES | 0.33 | [-0.03, 0.68] | 1.38 | [0.97, 1.98] | 0.619 |
|  | VU : RES | -0.21 | [-0.58, 0.15] | 0.81 | [0.56, 1.16] | 0.692 |
|  | HC : RES | 0.51 | [0.14, 0.87] | 1.66 | [1.15, 2.39] | 0.217 |
| Pericalcarine Cortex | MA : RES | -0.14 | [-0.51, 0.22] | 0.87 | [0.6, 1.25] | 0.692 |
|  | VU : RES | 0.18 | [-0.19, 0.55] | 1.20 | [0.83, 1.73] | 0.692 |
|  | HC : RES | 0.05 | [-0.07, 0.17] | 1.05 | [0.94, 1.19] | 0.763 |
| Postcentral Gyrus | MA : RES | 0.10 | [-0.02, 0.22] | 1.11 | [0.98, 1.24] | 0.763 |
|  | VU : RES | 0.09 | [-0.03, 0.21] | 1.09 | [0.97, 1.23] | 0.763 |
|  | HC : RES | 0.06 | [-0.06, 0.18] | 1.06 | [0.94, 1.2] | 0.763 |
| Precentral Gyrus | MA : RES | 0.00 | [-0.12, 0.12] | 1.00 | [0.88, 1.12] | 0.976 |
|  | VU : RES | 0.04 | [-0.08, 0.16] | 1.04 | [0.92, 1.17] | 0.763 |
|  | HC : RES | 0.05 | [-0.07, 0.17] | 1.05 | [0.93, 1.18] | 0.763 |
| Posterior Cingulate Cortex | MA : RES | -0.06 | [-0.17, 0.06] | 0.95 | [0.84, 1.06] | 0.763 |
|  | VU : RES | 0.01 | [-0.11, 0.13] | 1.01 | [0.9, 1.13] | 0.959 |
|  | HC : RES | 0.05 | [-0.07, 0.16] | 1.05 | [0.93, 1.18] | 0.763 |
| Rostral Anterior Cingulate Cortex | MA : RES | 0.06 | [-0.06, 0.18] | 1.07 | [0.94, 1.2] | 0.763 |
|  | VU : RES | 0.04 | [-0.08, 0.15] | 1.04 | [0.92, 1.17] | 0.786 |
|  | HC : RES | -0.01 | [-0.13, 0.11] | 0.99 | [0.88, 1.11] | 0.935 |
| Rostral Middle Frontal Gyrus | MA : RES | 0.02 | [-0.1, 0.13] | 1.02 | [0.9, 1.14] | 0.935 |
|  | VU : RES | 0.06 | [-0.06, 0.18] | 1.06 | [0.94, 1.2] | 0.763 |
|  | HC : RES | 0.04 | [-0.08, 0.16] | 1.04 | [0.92, 1.17] | 0.763 |
| Supramarginal Gyrus | MA : RES | 0.05 | [-0.07, 0.17] | 1.05 | [0.93, 1.18] | 0.763 |
|  | VU : RES | 0.00 | [-0.12, 0.12] | 1.00 | [0.88, 1.13] | 0.978 |
|  | HC : RES | 0.04 | [-0.08, 0.16] | 1.04 | [0.92, 1.17] | 0.763 |
| Superior Frontal Gyrus | MA : RES | 0.09 | [-0.03, 0.2] | 1.09 | [0.97, 1.23] | 0.763 |
|  | VU : RES | 0.01 | [-0.1, 0.13] | 1.01 | [0.9, 1.14] | 0.935 |
|  | HC : RES | 0.03 | [-0.09, 0.15] | 1.03 | [0.91, 1.16] | 0.823 |
| Superior Parietal Lobule | MA : RES | -0.01 | [-0.13, 0.11] | 0.99 | [0.88, 1.11] | 0.935 |
|  | VU : RES | 0.04 | [-0.07, 0.16] | 1.05 | [0.93, 1.18] | 0.763 |
|  | HC : RES | 0.09 | [-0.03, 0.21] | 1.10 | [0.97, 1.24] | 0.763 |
| Superior Temporal Gyrus | MA : RES | 0.05 | [-0.07, 0.16] | 1.05 | [0.93, 1.18] | 0.763 |
|  | VU : RES | 0.10 | [-0.02, 0.22] | 1.11 | [0.98, 1.25] | 0.763 |
|  | HC : RES | 0.06 | [-0.06, 0.18] | 1.07 | [0.95, 1.2] | 0.763 |
| Temporal Pole | MA : RES | 0.07 | [-0.05, 0.19] | 1.08 | [0.95, 1.21] | 0.763 |
|  | VU : RES | 0.05 | [-0.07, 0.17] | 1.05 | [0.93, 1.18] | 0.763 |
|  | HC : RES | -0.03 | [-0.15, 0.09] | 0.97 | [0.86, 1.09] | 0.823 |
| Transverse Temporal Gyrus | MA : RES | 0.13 | [0.01, 0.25] | 1.14 | [1.01, 1.29] | 0.763 |
|  | VU : RES | 0.03 | [-0.09, 0.15] | 1.03 | [0.91, 1.16] | 0.823 |
|  | HC : RES | -0.04 | [-0.16, 0.08] | 0.96 | [0.85, 1.08] | 0.763 |
| *Cortical thickness* |  |  |  |  |  |  |
| Banks of Superior Temporal Sulcus | MA : RES | -0.04 | [-0.46, 0.37] | 0.96 | [0.63, 1.45] | 0.894 |
|  | VU : RES | -0.19 | [-0.59, 0.22] | 0.83 | [0.55, 1.24] | 0.523 |
|  | HC : RES | -0.35 | [-0.76, 0.06] | 0.71 | [0.47, 1.07] | 0.258 |
| Caudal Anterior Cingulate Cortex | MA : RES | -0.25 | [-0.64, 0.14] | 0.78 | [0.53, 1.15] | 0.385 |
|  | VU : RES | -0.37 | [-0.79, 0.04] | 0.69 | [0.45, 1.04] | 0.241 |
|  | HC : RES | -0.40 | [-0.83, 0.02] | 0.67 | [0.44, 1.02] | 0.241 |
| Caudal Middle Frontal Gryus | MA : RES | -0.28 | [-0.68, 0.13] | 0.76 | [0.5, 1.14] | 0.376 |
|  | VU : RES | -0.26 | [-0.67, 0.15] | 0.77 | [0.51, 1.16] | 0.386 |
|  | HC : RES | 0.08 | [-0.34, 0.5] | 1.08 | [0.71, 1.64] | 0.828 |
| Cuneus | MA : RES | -0.28 | [-0.69, 0.14] | 0.76 | [0.5, 1.15] | 0.376 |
|  | VU : RES | -0.06 | [-0.46, 0.34] | 0.94 | [0.63, 1.41] | 0.853 |
|  | HC : RES | -0.22 | [-0.6, 0.16] | 0.80 | [0.55, 1.18] | 0.431 |
| Entorhinal Cortex | MA : RES | -0.36 | [-0.78, 0.05] | 0.70 | [0.46, 1.05] | 0.241 |
|  | VU : RES | -0.09 | [-0.51, 0.32] | 0.91 | [0.6, 1.38] | 0.810 |
|  | HC : RES | -0.37 | [-0.78, 0.03] | 0.69 | [0.46, 1.03] | 0.241 |
| Frontal Pole | MA : RES | -0.43 | [-0.85, -0.02] | 0.65 | [0.43, 0.98] | 0.241 |
|  | VU : RES | -0.32 | [-0.74, 0.1] | 0.72 | [0.48, 1.1] | 0.322 |
|  | HC : RES | -0.02 | [-0.44, 0.4] | 0.98 | [0.65, 1.49] | 0.948 |
| Fusiform Area | MA : RES | -0.01 | [-0.42, 0.4] | 0.99 | [0.66, 1.49] | 0.964 |
|  | VU : RES | -0.10 | [-0.48, 0.27] | 0.90 | [0.62, 1.31] | 0.759 |
|  | HC : RES | -0.38 | [-0.8, 0.03] | 0.68 | [0.45, 1.03] | 0.241 |
| Inferior Parietal Lobule | MA : RES | -0.37 | [-0.78, 0.05] | 0.69 | [0.46, 1.05] | 0.241 |
|  | VU : RES | -0.40 | [-0.83, 0.03] | 0.67 | [0.43, 1.03] | 0.241 |
|  | HC : RES | -0.20 | [-0.61, 0.21] | 0.82 | [0.54, 1.24] | 0.523 |
| Inferior Temporal Cortex | MA : RES | -0.40 | [-0.81, 0] | 0.67 | [0.45, 1] | 0.241 |
|  | VU : RES | -0.36 | [-0.76, 0.05] | 0.70 | [0.47, 1.05] | 0.241 |
|  | HC : RES | -0.51 | [-0.94, -0.09] | 0.60 | [0.39, 0.92] | 0.241 |
| Isthmus Cingulate Gyrus | MA : RES | -0.23 | [-0.63, 0.17] | 0.80 | [0.53, 1.19] | 0.431 |
|  | VU : RES | -0.11 | [-0.53, 0.31] | 0.89 | [0.59, 1.36] | 0.759 |
|  | HC : RES | -0.14 | [-0.56, 0.29] | 0.87 | [0.57, 1.34] | 0.724 |
| Insula | MA : RES | -0.07 | [-0.48, 0.35] | 0.93 | [0.62, 1.42] | 0.847 |
|  | VU : RES | -0.37 | [-0.79, 0.05] | 0.69 | [0.46, 1.05] | 0.241 |
|  | HC : RES | -0.28 | [-0.69, 0.13] | 0.76 | [0.5, 1.14] | 0.376 |
| Lingual Gyrus | MA : RES | -0.19 | [-0.6, 0.21] | 0.83 | [0.55, 1.24] | 0.523 |
|  | VU : RES | -0.04 | [-0.43, 0.35] | 0.96 | [0.65, 1.42] | 0.960 |
|  | HC : RES | -0.25 | [-0.64, 0.13] | 0.78 | [0.53, 1.14] | 0.623 |
| Lateral Orbitofrontal Cortex | MA : RES | -0.40 | [-0.79, -0.01] | 0.67 | [0.45, 0.99] | 0.573 |
|  | VU : RES | -0.20 | [-0.58, 0.17] | 0.82 | [0.56, 1.19] | 0.677 |
|  | HC : RES | -0.20 | [-0.59, 0.18] | 0.82 | [0.55, 1.2] | 0.677 |
| Lateral Occipital Cortex | MA : RES | -0.35 | [-0.76, 0.06] | 0.70 | [0.47, 1.06] | 0.573 |
|  | VU : RES | -0.12 | [-0.52, 0.27] | 0.88 | [0.6, 1.31] | 0.788 |
|  | HC : RES | -0.14 | [-0.53, 0.25] | 0.87 | [0.59, 1.28] | 0.787 |
| Middle Temporal Gyrus | MA : RES | -0.16 | [-0.55, 0.23] | 0.86 | [0.58, 1.26] | 0.770 |
|  | VU : RES | -0.17 | [-0.57, 0.23] | 0.85 | [0.57, 1.26] | 0.770 |
|  | HC : RES | -0.13 | [-0.51, 0.25] | 0.88 | [0.6, 1.29] | 0.787 |
| Medial Orbitofrontal Cortex | MA : RES | -0.20 | [-0.58, 0.17] | 0.82 | [0.56, 1.18] | 0.677 |
|  | VU : RES | -0.33 | [-0.72, 0.06] | 0.72 | [0.49, 1.07] | 0.573 |
|  | HC : RES | -0.24 | [-0.63, 0.15] | 0.78 | [0.53, 1.16] | 0.623 |
| Paracentral Lobule | MA : RES | -0.16 | [-0.54, 0.23] | 0.85 | [0.58, 1.26] | 0.770 |
|  | VU : RES | -0.38 | [-0.77, 0.02] | 0.69 | [0.46, 1.02] | 0.573 |
|  | HC : RES | -0.18 | [-0.57, 0.21] | 0.83 | [0.57, 1.23] | 0.767 |
| Parahippocampal Gyrus | MA : RES | -0.07 | [-0.47, 0.33] | 0.93 | [0.63, 1.39] | 0.882 |
|  | VU : RES | 0.01 | [-0.37, 0.39] | 1.01 | [0.69, 1.48] | 0.960 |
|  | HC : RES | -0.10 | [-0.46, 0.27] | 0.91 | [0.63, 1.3] | 0.788 |
| Pars Orbitalis | MA : RES | -0.34 | [-0.74, 0.05] | 0.71 | [0.48, 1.05] | 0.573 |
|  | VU : RES | -0.29 | [-0.68, 0.09] | 0.75 | [0.51, 1.1] | 0.623 |
|  | HC : RES | -0.36 | [-0.76, 0.04] | 0.70 | [0.47, 1.04] | 0.573 |
| Pars Opercularis | MA : RES | -0.11 | [-0.51, 0.29] | 0.89 | [0.6, 1.33] | 0.788 |
|  | VU : RES | -0.13 | [-0.52, 0.25] | 0.87 | [0.59, 1.29] | 0.787 |
|  | HC : RES | -0.03 | [-0.42, 0.36] | 0.97 | [0.66, 1.44] | 0.960 |
| Pars Triangularis | MA : RES | -0.27 | [-0.67, 0.13] | 0.76 | [0.51, 1.14] | 0.623 |
|  | VU : RES | -0.10 | [-0.48, 0.28] | 0.90 | [0.62, 1.32] | 0.788 |
|  | HC : RES | -0.25 | [-0.65, 0.14] | 0.78 | [0.52, 1.15] | 0.623 |
| Precuneus | MA : RES | 0.01 | [-0.39, 0.41] | 1.01 | [0.68, 1.51] | 0.960 |
|  | VU : RES | 0.03 | [-0.37, 0.43] | 1.03 | [0.69, 1.53] | 0.960 |
|  | HC : RES | -0.28 | [-0.67, 0.12] | 0.76 | [0.51, 1.13] | 0.623 |
| Pericalcarine Cortex | MA : RES | -0.01 | [-0.39, 0.37] | 0.99 | [0.67, 1.45] | 0.960 |
|  | VU : RES | -0.08 | [-0.48, 0.32] | 0.92 | [0.62, 1.37] | 0.857 |
|  | HC : RES | -0.03 | [-0.15, 0.1] | 0.97 | [0.86, 1.1] | 0.876 |
| Postcentral Gyrus | MA : RES | -0.06 | [-0.19, 0.06] | 0.94 | [0.83, 1.06] | 0.694 |
|  | VU : RES | 0.00 | [-0.13, 0.12] | 1.00 | [0.88, 1.13] | 0.993 |
|  | HC : RES | -0.03 | [-0.15, 0.1] | 0.97 | [0.86, 1.1] | 0.876 |
| Precentral Gyrus | MA : RES | -0.07 | [-0.19, 0.06] | 0.94 | [0.82, 1.06] | 0.694 |
|  | VU : RES | -0.12 | [-0.25, 0.02] | 0.89 | [0.78, 1.02] | 0.354 |
|  | HC : RES | -0.11 | [-0.24, 0.01] | 0.89 | [0.79, 1.01] | 0.354 |
| Posterior Cingulate Cortex | MA : RES | -0.11 | [-0.24, 0.02] | 0.90 | [0.79, 1.02] | 0.359 |
|  | VU : RES | -0.05 | [-0.18, 0.08] | 0.95 | [0.84, 1.08] | 0.695 |
|  | HC : RES | -0.02 | [-0.15, 0.11] | 0.98 | [0.86, 1.12] | 0.934 |
| Rostral Anterior Cingulate Cortex | MA : RES | -0.08 | [-0.2, 0.05] | 0.93 | [0.82, 1.05] | 0.652 |
|  | VU : RES | 0.00 | [-0.12, 0.12] | 1.00 | [0.88, 1.13] | 0.993 |
|  | HC : RES | -0.13 | [-0.26, 0] | 0.88 | [0.77, 1] | 0.354 |
| Rostral Middle Frontal Gyrus | MA : RES | -0.06 | [-0.19, 0.07] | 0.94 | [0.83, 1.07] | 0.694 |
|  | VU : RES | -0.05 | [-0.17, 0.08] | 0.95 | [0.84, 1.08] | 0.695 |
|  | HC : RES | -0.09 | [-0.22, 0.03] | 0.91 | [0.8, 1.03] | 0.515 |
| Supramarginal Gyrus | MA : RES | -0.05 | [-0.18, 0.07] | 0.95 | [0.84, 1.07] | 0.694 |
|  | VU : RES | 0.00 | [-0.13, 0.13] | 1.00 | [0.88, 1.14] | 0.993 |
|  | HC : RES | -0.07 | [-0.2, 0.05] | 0.93 | [0.82, 1.05] | 0.658 |
| Superior Frontal Gyrus | MA : RES | -0.01 | [-0.13, 0.11] | 0.99 | [0.88, 1.12] | 0.979 |
|  | VU : RES | -0.08 | [-0.21, 0.05] | 0.92 | [0.81, 1.05] | 0.646 |
|  | HC : RES | -0.11 | [-0.24, 0.01] | 0.89 | [0.79, 1.01] | 0.354 |
| Superior Parietal Lobule | MA : RES | -0.05 | [-0.18, 0.08] | 0.95 | [0.83, 1.08] | 0.695 |
|  | VU : RES | -0.06 | [-0.19, 0.06] | 0.94 | [0.82, 1.07] | 0.694 |
|  | HC : RES | -0.12 | [-0.25, 0] | 0.89 | [0.78, 1] | 0.354 |
| Superior Temporal Gyrus | MA : RES | -0.05 | [-0.18, 0.07] | 0.95 | [0.83, 1.08] | 0.694 |
|  | VU : RES | -0.13 | [-0.26, -0.01] | 0.87 | [0.77, 0.99] | 0.354 |
|  | HC : RES | -0.01 | [-0.14, 0.11] | 0.99 | [0.87, 1.12] | 0.960 |
| Temporal Pole | MA : RES | -0.05 | [-0.17, 0.08] | 0.96 | [0.84, 1.09] | 0.695 |
|  | VU : RES | 0.02 | [-0.11, 0.15] | 1.02 | [0.9, 1.16] | 0.934 |
|  | HC : RES | 0.00 | [-0.13, 0.13] | 1.00 | [0.88, 1.14] | 0.993 |
| Transverse Temporal Gyrus | MA : RES | -0.19 | [-0.33, -0.06] | 0.82 | [0.72, 0.94] | 0.114 |
|  | VU : RES | -0.06 | [-0.18, 0.07] | 0.94 | [0.83, 1.07] | 0.694 |
|  | HC : RES | -0.15 | [-0.28, -0.02] | 0.86 | [0.76, 0.98] | 0.354 |
| *Subcortical volume* |  |  |  |  |  |  |
| Accumbens | MA : RES | -0.36 | [-0.75, 0.02] | 0.70 | [0.47, 1.02] | 0.231 |
|  | VU : RES | -0.24 | [-0.65, 0.17] | 0.79 | [0.52, 1.19] | 0.446 |
|  | HC : RES | -0.15 | [-0.56, 0.26] | 0.86 | [0.57, 1.3] | 0.484 |
| Amygdala | MA : RES | -0.16 | [-0.57, 0.25] | 0.85 | [0.56, 1.28] | 0.484 |
|  | VU : RES | -0.29 | [-0.71, 0.13] | 0.75 | [0.49, 1.14] | 0.410 |
|  | HC : RES | -0.15 | [-0.56, 0.25] | 0.86 | [0.57, 1.29] | 0.484 |
| Caudate | MA : RES | -0.43 | [-0.85, -0.02] | 0.65 | [0.43, 0.98] | 0.231 |
|  | VU : RES | -0.18 | [-0.56, 0.19] | 0.83 | [0.57, 1.21] | 0.886 |
|  | HC : RES | -0.07 | [-0.46, 0.31] | 0.93 | [0.63, 1.37] | 0.886 |
| Hippocampus | MA : RES | -0.04 | [-0.43, 0.35] | 0.96 | [0.65, 1.41] | 0.886 |
|  | VU : RES | -0.06 | [-0.44, 0.33] | 0.95 | [0.64, 1.39] | 0.886 |
|  | HC : RES | -0.03 | [-0.43, 0.37] | 0.97 | [0.65, 1.45] | 0.886 |
| Pallidum | MA : RES | -0.46 | [-0.84, -0.08] | 0.63 | [0.43, 0.92] | 0.120 |
|  | VU : RES | -0.07 | [-0.46, 0.32] | 0.94 | [0.63, 1.38] | 0.886 |
|  | HC : RES | -0.07 | [-0.19, 0.05] | 0.93 | [0.82, 1.05] | 0.595 |
| Putamen | MA : RES | -0.09 | [-0.22, 0.03] | 0.91 | [0.8, 1.04] | 0.549 |
|  | VU : RES | 0.00 | [-0.13, 0.13] | 1.00 | [0.88, 1.14] | 0.972 |
|  | HC : RES | -0.04 | [-0.17, 0.08] | 0.96 | [0.84, 1.09] | 0.595 |
| Thalamus | MA : RES | -0.09 | [-0.22, 0.04] | 0.91 | [0.8, 1.04] | 0.549 |
|  | VU : RES | -0.04 | [-0.17, 0.08] | 0.96 | [0.85, 1.09] | 0.595 |
|  | HC : RES | -0.04 | [-0.17, 0.09] | 0.96 | [0.84, 1.09] | 0.595 |

## Table S5. Post-hoc analyses for significant sex interaction results separately in males and females

| Brain variables | Group comparisons | Males | | | Females | | | | |  |  |
| --- | --- | --- | --- | --- | --- | --- | --- | --- | --- | --- | --- |
|  |  | Odds (95% CI) | | *p* | Odds (95% CI) | | | *p* | |  |  |
| *Surface area* |  |  | |  |  | | |  | |  |  |
| Banks of Superior Temporal Sulcus | MA : RES | 1.11 [0.88, 1.39] | | .375 | 0.86 [0.66, 1.12] | | | .261 | |  |  |
|  | VU : RES | 0.88 [0.72, 1.07] | | .189 | 0.83 [0.66, 1.05] | | | .127 | |  |  |
|  | HC : RES | 0.93 [0.82, 1.05] | | .224 | **0.86 [0.75, 1.00]** | | | **.045** | |  |  |
| Frontal Pole | MA : RES | 1.03 [0.81, 1.31] | | .819 | 0.86 [0.65, 1.13] | | | .284 | |  |  |
|  | VU : RES | 0.94 [0.77, 1.16] | | .582 | 0.88 [0.69, 1.12] | | | .305 | |  |  |
|  | HC : RES | 0.92 [0.81, 1.05] | | .242 | 0.90 [0.77, 1.05] | | | .174 | |  |  |
| Fusiform Area | MA : RES | 1.32 [1.00, 1.75] | | .051 | 0.84 [0.61, 1.14] | | | .263 | |  |  |
|  | VU : RES | 1.11 [0.87, 1.41] | | .397 | 0.89 [0.68, 1.17] | | | .421 | |  |  |
|  | HC : RES | 1.07 [0.92, 1.25] | | .403 | 0.88 [0.74, 1.04] | | | .131 | |  |  |
| Inferior Parietal | MA : RES | 1.08 [0.84, 1.39] | | .551 | 0.85 [0.64, 1.12] | | | .249 | |  |  |
|  | VU : RES | 1.02 [0.82, 1.26] | | .871 | 0.94 [0.74, 1.19] | | | .601 | |  |  |
|  | HC : RES | 1.07 [0.93, 1.23] | | .323 | 0.87 [0.75, 1.02] | | | .080 | |  |  |
| Medial Orbitofrontal | MA : RES | 1.13 [0.85, 1.51] | | .403 | 0.96 [0.69, 1.32] | | | .792 | |  |  |
|  | VU : RES | 0.85 [0.67, 1.09] | | .205 | 0.90 [0.68, 1.19] | | | .472 | |  |  |
|  | HC : RES | 1.02 [0.87, 1.2] | | .792 | 0.93 [0.78, 1.11] | | | .412 | |  |  |
| Paracentral | MA : RES | 0.94 [0.75, 1.18] | | .579 | **0.74 [0.56, 0.97]** | | | **.030** | |  |  |
|  | VU : RES | 0.96 [0.79, 1.17] | | .704 | 0.92 [0.73, 1.16] | | | .471 | |  |  |
|  | HC : RES | 1.00 [0.88, 1.13] | | .960 | 0.93 [0.80, 1.08] | | | .329 | |  |  |
| Pars Orbitalis | MA : RES | 1.17 [0.91, 1.51] | | .227 | 0.95 [0.71, 1.26] | | | .701 | |  |  |
|  | VU : RES | 1.19 [0.96, 1.49] | | .111 | 1.17 [0.92, 1.49] | | | .211 | |  |  |
|  | HC : RES | 1.02 [0.88, 1.17] | | .820 | 1.12 [0.96, 1.31] | | | .134 | |  |  |
| Precentral | MA : RES | 1.02 [0.78, 1.34] | | .876 | 0.85 [0.62, 1.16] | | | .294 | |  |  |
|  | VU : RES | 0.95 [0.75, 1.20] | | .664 | 1.01 [0.77, 1.33] | | | .917 | |  |  |
|  | HC : RES | 1.01 [0.87, 1.18] | | .847 | 1.13 [0.96, 1.34] | | | .145 | |  |  |
| Posterior Cingulate | MA : RES | 0.97 [0.76, 1.24] | | .799 | 0.83 [0.62, 1.10] | | | .197 | |  |  |
|  | VU : RES | 0.96 [0.78, 1.18] | | .689 | 0.95 [0.74, 1.21] | | | .665 | |  |  |
|  | HC : RES | 1.11 [0.97, 1.27] | | .139 | 0.92 [0.79, 1.08] | | | .300 | |  |  |
| Rostral Anterior Cingulate | MA : RES | 1.28 [0.99, 1.66] | | .060 | 1.07 [0.81, 1.41] | | | .647 | |  |  |
|  | VU : RES | 0.92 [0.73, 1.15] | | .449 | 1.06 [0.83, 1.36] | | | .620 | |  |  |
|  | HC : RES | 1.10 [0.96, 1.27] | | .181 | 1.03 [0.88, 1.20] | | | .729 | |  |  |
| Supramarginal | MA : RES | 0.89 [0.70, 1.13] | | .341 | **0.74 [0.54, 1.00]** | | | **.046** | |  |  |
|  | VU : RES | 0.92 [0.75, 1.13] | | .454 | 0.97 [0.75, 1.26] | | | .845 | |  |  |
|  | HC : RES | **0.86 [0.76, 0.99]** | | **.030** | 0.93 [0.79, 1.09] | | | .378 | |  |  |
| Superior Frontal | MA : RES | 0.80 [0.60, 1.08] | | .153 | 0.72 [0.52, 1.01] | | | .058 | |  |  |
|  | VU : RES | 0.89 [0.69, 1.15] | | .370 | **0.66 [0.49, 0.88]** | | | **.005** | |  |  |
|  | HC : RES | 0.96 [0.82, 1.12] | | .587 | 0.84 [0.70, 1.00] | | | .053 | |  |  |
| *Subcortical volume* | |  | |  | | |  |  | |  | |
| Accumbens | | MA : RES | 1.24 [0.98, 1.57] | | .076 | 1.00 [0.78, 1.29] | | | .987 | |  |
|  |  | VU : RES | 1.01 [0.83, 1.24] | | .889 | 0.87 [0.70, 1.08] | | | .212 | |  |
|  |  | HC : RES | 1.12 [0.98, 1.27] | | .091 | 1.04 [0.91, 1.19] | | | .566 | |  |
| Amygdala | | MA : RES | 1.11 [0.87, 1.43] | | .397 | 1.19 [0.91, 1.55] | | | .213 | |  |
|  |  | VU : RES | **1.41 [1.14, 1.74]** | | **.001** | 0.84 [0.66, 1.08] | | | .179 | |  |
|  |  | HC : RES | 1.12 [0.97, 1.28] | | .112 | 0.9 [0.77, 1.04] | | | .156 | |  |
| Hippocampus | | MA : RES | 0.97 [0.75, 1.25] | | .805 | 0.9 [0.68, 1.19] | | | .459 | |  |
|  |  | VU : RES | 1.22 [0.99, 1.51] | | .066 | 0.96 [0.76, 1.22] | | | .763 | |  |
|  |  | HC : RES | **1.18 [1.03, 1.35]** | | **.020** | 0.88 [0.76, 1.02] | | | .098 | |  |

*Note.* All results pertain to internalising group comparisons. Bolded text indicates significant results within subsamples of males and females (uncorrected *p* <. 05). Values > 1 indicate increased odds and < 1 indicate decreased odds of being maladaptive/vulnerable/healthy vs. resilient to internalizing symptoms group, with increases in brain structure/connectivity. Abbreviations: confidence interval (CI), healthy control (HC), maladaptive (MA), vulnerable (VU), resilient (RES).

# Appendix S8. Full sample results – White matter structure

## Main effects

### Table S6. Internalizing group comparisons

| **Variables** | **Group comparisons** | **B** | **95% CI** | **Odds** | **95% CI** | ***p*FDR** |
| --- | --- | --- | --- | --- | --- | --- |
| Anterior thalamus | MA : RES | -0.08 | [-0.27, 0.11] | 0.92 | [0.77, 1.12] | 0.814 |
|  | VU : RES | -0.02 | [-0.17, 0.14] | 0.98 | [0.84, 1.15] | 0.815 |
|  | HC : RES | -0.02 | [-0.09, 0.06] | 0.98 | [0.91, 1.06] | 0.730 |
| Cingulate cingulum | MA : RES | -0.07 | [-0.24, 0.09] | 0.93 | [0.79, 1.1] | 0.814 |
|  | VU : RES | -0.13 | [-0.27, 0.01] | 0.87 | [0.76, 1.01] | 0.268 |
|  | HC : RES | -0.04 | [-0.11, 0.02] | 0.96 | [0.9, 1.02] | 0.577 |
| Corpus callosum | MA : RES | -0.09 | [-0.28, 0.1] | 0.91 | [0.75, 1.11] | 0.814 |
|  | VU : RES | -0.13 | [-0.29, 0.03] | 0.88 | [0.75, 1.03] | 0.268 |
|  | HC : RES | -0.01 | [-0.09, 0.06] | 0.99 | [0.91, 1.07] | 0.771 |
| Corticospinal | MA : RES | -0.11 | [-0.29, 0.07] | 0.90 | [0.75, 1.07] | 0.708 |
|  | VU : RES | -0.05 | [-0.21, 0.1] | 0.95 | [0.81, 1.1] | 0.608 |
|  | HC : RES | 0.03 | [-0.04, 0.1] | 1.03 | [0.96, 1.11] | 0.707 |
| Forceps major | MA : RES | 0.00 | [-0.18, 0.18] | 1.00 | [0.83, 1.2] | 0.994 |
|  | VU : RES | -0.10 | [-0.26, 0.05] | 0.90 | [0.77, 1.05] | 0.303 |
|  | HC : RES | -0.06 | [-0.13, 0.02] | 0.95 | [0.88, 1.02] | 0.577 |
| Forceps minor | MA : RES | -0.07 | [-0.26, 0.11] | 0.93 | [0.77, 1.12] | 0.814 |
|  | VU : RES | -0.12 | [-0.28, 0.03] | 0.88 | [0.76, 1.03] | 0.268 |
|  | HC : RES | -0.02 | [-0.09, 0.05] | 0.98 | [0.91, 1.06] | 0.730 |
| Fornix | MA : RES | -0.01 | [-0.2, 0.18] | 0.99 | [0.82, 1.2] | 0.994 |
|  | VU : RES | -0.19 | [-0.36, -0.02] | 0.83 | [0.7, 0.98] | 0.268 |
|  | HC : RES | -0.05 | [-0.13, 0.03] | 0.95 | [0.88, 1.03] | 0.577 |
| Inferior longitudinal fasiculus | MA : RES | -0.13 | [-0.32, 0.05] | 0.88 | [0.73, 1.05] | 0.570 |
|  | VU : RES | -0.12 | [-0.27, 0.04] | 0.89 | [0.76, 1.04] | 0.268 |
|  | HC : RES | -0.02 | [-0.1, 0.05] | 0.98 | [0.91, 1.05] | 0.730 |
| Inferior frontal superior frontal cortex | MA : RES | -0.04 | [-0.21, 0.13] | 0.96 | [0.81, 1.14] | 0.925 |
|  | VU : RES | -0.03 | [-0.18, 0.12] | 0.97 | [0.84, 1.13] | 0.725 |
|  | HC : RES | 0.05 | [-0.01, 0.12] | 1.06 | [0.99, 1.13] | 0.577 |
| Inferior fronto-occipital fasiculus | MA : RES | -0.16 | [-0.35, 0.04] | 0.85 | [0.7, 1.04] | 0.570 |
|  | VU : RES | -0.16 | [-0.32, 0.01] | 0.86 | [0.73, 1.01] | 0.268 |
|  | HC : RES | -0.11 | [-0.19, -0.03] | 0.89 | [0.83, 0.97] | 0.083 |
| Parahippocampal cingulum | MA : RES | -0.06 | [-0.26, 0.14] | 0.94 | [0.77, 1.15] | 0.925 |
|  | VU : RES | -0.14 | [-0.31, 0.04] | 0.87 | [0.73, 1.04] | 0.268 |
|  | HC : RES | -0.05 | [-0.13, 0.03] | 0.95 | [0.88, 1.03] | 0.577 |
| Parietal superior longitudinal fasiculus | MA : RES | -0.04 | [-0.21, 0.14] | 0.96 | [0.81, 1.15] | 0.925 |
|  | VU : RES | -0.12 | [-0.27, 0.03] | 0.89 | [0.76, 1.03] | 0.268 |
|  | HC : RES | -0.02 | [-0.09, 0.05] | 0.98 | [0.91, 1.05] | 0.730 |
| Striatal inferior frontal cortex | MA : RES | -0.04 | [-0.22, 0.14] | 0.96 | [0.8, 1.15] | 0.925 |
|  | VU : RES | -0.07 | [-0.23, 0.08] | 0.93 | [0.8, 1.08] | 0.471 |
|  | HC : RES | -0.03 | [-0.1, 0.05] | 0.97 | [0.91, 1.05] | 0.730 |
| Superior corticostriate-frontal cortex | MA : RES | -0.13 | [-0.29, 0.04] | 0.88 | [0.75, 1.04] | 0.570 |
|  | VU : RES | -0.09 | [-0.23, 0.05] | 0.91 | [0.79, 1.05] | 0.303 |
|  | HC : RES | 0.02 | [-0.04, 0.09] | 1.03 | [0.96, 1.09] | 0.730 |
| Superior corticostriate-parietal cortex | MA : RES | -0.18 | [-0.35, -0.01] | 0.84 | [0.7, 0.99] | 0.471 |
|  | VU : RES | -0.04 | [-0.18, 0.11] | 0.96 | [0.83, 1.11] | 0.676 |
|  | HC : RES | 0.04 | [-0.03, 0.11] | 1.04 | [0.97, 1.11] | 0.577 |
| Superior corticostriatum | MA : RES | -0.17 | [-0.35, 0] | 0.84 | [0.71, 1] | 0.471 |
|  | VU : RES | -0.05 | [-0.2, 0.1] | 0.95 | [0.82, 1.1] | 0.608 |
|  | HC : RES | 0.04 | [-0.03, 0.11] | 1.04 | [0.97, 1.11] | 0.577 |
| Superior longitudinal fasiculus | MA : RES | 0.00 | [-0.17, 0.17] | 1.00 | [0.84, 1.19] | 0.994 |
|  | VU : RES | -0.11 | [-0.26, 0.04] | 0.89 | [0.77, 1.04] | 0.268 |
|  | HC : RES | -0.02 | [-0.09, 0.05] | 0.98 | [0.92, 1.05] | 0.730 |
| Temporal superior longitudinal fasiculus | MA : RES | 0.02 | [-0.15, 0.19] | 1.02 | [0.86, 1.21] | 0.945 |
|  | VU : RES | -0.10 | [-0.25, 0.05] | 0.91 | [0.78, 1.05] | 0.303 |
|  | HC : RES | -0.01 | [-0.08, 0.06] | 0.99 | [0.92, 1.06] | 0.784 |
| Uncinate | MA : RES | -0.02 | [-0.21, 0.16] | 0.98 | [0.81, 1.17] | 0.945 |
|  | VU : RES | -0.14 | [-0.3, 0.02] | 0.87 | [0.74, 1.02] | 0.268 |
|  | HC : RES | -0.08 | [-0.15, 0] | 0.93 | [0.86, 1] | 0.422 |

### Table S7. Externalising group comparisons

| **Variables** | **Group comparisons** | **B** | **95% CI** | **Odds** | **95% CI** | ***p*FDR** |
| --- | --- | --- | --- | --- | --- | --- |
| Anterior thalamus | MA : RES | -0.03 | [-0.31, 0.25] | 0.92 | [0.73, 1.28] | 0.865 |
|  | VU : RES | -0.08 | [-0.36, 0.2] | 0.98 | [0.7, 1.22] | 0.666 |
|  | HC : RES | -0.01 | [-0.08, 0.06] | 0.98 | [0.92, 1.06] | 0.785 |
| Cingulate cingulum | MA : RES | 0.09 | [-0.16, 0.33] | 0.93 | [0.85, 1.39] | 0.765 |
|  | VU : RES | -0.07 | [-0.32, 0.18] | 0.87 | [0.73, 1.2] | 0.666 |
|  | HC : RES | -0.04 | [-0.1, 0.02] | 0.96 | [0.9, 1.02] | 0.420 |
| Corpus callosum | MA : RES | -0.26 | [-0.53, 0.02] | 0.91 | [0.59, 1.02] | 0.430 |
|  | VU : RES | -0.24 | [-0.52, 0.04] | 0.88 | [0.59, 1.04] | 0.292 |
|  | HC : RES | -0.02 | [-0.09, 0.05] | 0.99 | [0.91, 1.06] | 0.635 |
| Corticospinal | MA : RES | 0.13 | [-0.13, 0.38] | 0.90 | [0.88, 1.47] | 0.685 |
|  | VU : RES | -0.02 | [-0.29, 0.25] | 0.95 | [0.75, 1.28] | 0.878 |
|  | HC : RES | 0.04 | [-0.03, 0.11] | 1.03 | [0.97, 1.12] | 0.420 |
| Forceps major | MA : RES | -0.12 | [-0.39, 0.15] | 1.00 | [0.68, 1.17] | 0.685 |
|  | VU : RES | -0.16 | [-0.43, 0.11] | 0.90 | [0.65, 1.11] | 0.453 |
|  | HC : RES | -0.06 | [-0.13, 0.01] | 0.95 | [0.88, 1.01] | 0.369 |
| Forceps minor | MA : RES | -0.09 | [-0.36, 0.19] | 0.93 | [0.7, 1.21] | 0.765 |
|  | VU : RES | -0.10 | [-0.37, 0.18] | 0.88 | [0.69, 1.19] | 0.666 |
|  | HC : RES | -0.02 | [-0.09, 0.05] | 0.98 | [0.91, 1.05] | 0.635 |
| Fornix | MA : RES | -0.03 | [-0.33, 0.27] | 0.99 | [0.72, 1.3] | 0.865 |
|  | VU : RES | -0.09 | [-0.39, 0.2] | 0.83 | [0.68, 1.23] | 0.666 |
|  | HC : RES | -0.06 | [-0.13, 0.02] | 0.95 | [0.87, 1.02] | 0.369 |
| Inferior longitudinal fasiculus | MA : RES | -0.19 | [-0.47, 0.08] | 0.88 | [0.63, 1.08] | 0.501 |
|  | VU : RES | -0.03 | [-0.3, 0.24] | 0.89 | [0.74, 1.28] | 0.878 |
|  | HC : RES | -0.03 | [-0.1, 0.04] | 0.98 | [0.91, 1.04] | 0.580 |
| Inferior frontal superior frontal cortex | MA : RES | -0.21 | [-0.46, 0.04] | 0.96 | [0.63, 1.04] | 0.444 |
|  | VU : RES | -0.19 | [-0.45, 0.06] | 0.97 | [0.64, 1.07] | 0.334 |
|  | HC : RES | 0.05 | [-0.02, 0.12] | 1.06 | [0.98, 1.12] | 0.369 |
| Inferior fronto-occipital fasiculus | MA : RES | -0.16 | [-0.44, 0.13] | 0.85 | [0.64, 1.14] | 0.685 |
|  | VU : RES | -0.28 | [-0.57, 0.01] | 0.86 | [0.57, 1.01] | 0.240 |
|  | HC : RES | -0.10 | [-0.18, -0.03] | 0.89 | [0.84, 0.97] | 0.138 |
| Parahippocampal cingulum | MA : RES | -0.14 | [-0.44, 0.16] | 0.94 | [0.65, 1.18] | 0.685 |
|  | VU : RES | -0.21 | [-0.51, 0.09] | 0.87 | [0.6, 1.1] | 0.365 |
|  | HC : RES | -0.06 | [-0.13, 0.02] | 0.95 | [0.88, 1.02] | 0.369 |
| Parietal superior longitudinal fasiculus | MA : RES | -0.26 | [-0.51, -0.01] | 0.96 | [0.6, 0.99] | 0.430 |
|  | VU : RES | -0.29 | [-0.54, -0.03] | 0.89 | [0.58, 0.97] | 0.171 |
|  | HC : RES | -0.03 | [-0.1, 0.04] | 0.98 | [0.91, 1.04] | 0.580 |
| Striatal inferior frontal cortex | MA : RES | -0.18 | [-0.46, 0.09] | 0.96 | [0.63, 1.09] | 0.501 |
|  | VU : RES | -0.26 | [-0.53, 0.01] | 0.93 | [0.59, 1.01] | 0.240 |
|  | HC : RES | -0.03 | [-0.1, 0.04] | 0.97 | [0.91, 1.04] | 0.580 |
| Superior corticostriate-frontal cortex | MA : RES | -0.06 | [-0.31, 0.19] | 0.88 | [0.73, 1.21] | 0.812 |
|  | VU : RES | -0.13 | [-0.38, 0.12] | 0.91 | [0.68, 1.13] | 0.529 |
|  | HC : RES | 0.03 | [-0.03, 0.09] | 1.03 | [0.97, 1.1] | 0.580 |
| Superior corticostriate-parietal cortex | MA : RES | 0.04 | [-0.21, 0.29] | 0.84 | [0.81, 1.34] | 0.865 |
|  | VU : RES | -0.08 | [-0.34, 0.18] | 0.96 | [0.72, 1.2] | 0.666 |
|  | HC : RES | 0.05 | [-0.01, 0.12] | 1.04 | [0.99, 1.13] | 0.369 |
| Superior corticostriatum | MA : RES | -0.02 | [-0.28, 0.24] | 0.84 | [0.75, 1.27] | 0.865 |
|  | VU : RES | -0.10 | [-0.36, 0.16] | 0.95 | [0.69, 1.18] | 0.666 |
|  | HC : RES | 0.05 | [-0.01, 0.12] | 1.04 | [0.99, 1.13] | 0.369 |
| Superior longitudinal fasiculus | MA : RES | -0.25 | [-0.51, 0] | 1.00 | [0.6, 1] | 0.430 |
|  | VU : RES | -0.34 | [-0.59, -0.08] | 0.89 | [0.56, 0.92] | 0.084 |
|  | HC : RES | -0.03 | [-0.09, 0.04] | 0.98 | [0.91, 1.04] | 0.580 |
| Temporal superior longitudinal fasiculus | MA : RES | -0.21 | [-0.46, 0.05] | 1.02 | [0.63, 1.05] | 0.444 |
|  | VU : RES | -0.36 | [-0.61, -0.11] | 0.91 | [0.54, 0.9] | 0.084 |
|  | HC : RES | -0.02 | [-0.09, 0.05] | 0.99 | [0.92, 1.05] | 0.635 |
| Uncinate | MA : RES | -0.08 | [-0.36, 0.2] | 0.98 | [0.69, 1.22] | 0.765 |
|  | VU : RES | -0.22 | [-0.49, 0.06] | 0.87 | [0.61, 1.06] | 0.334 |
|  | HC : RES | -0.08 | [-0.15, -0.01] | 0.93 | [0.86, 0.99] | 0.300 |

## Sext interaction effects

### Table S8. Internalizing group comparisons

| **Variables** | **Group comparisons** | **B** | **95% CI** | **Odds** | **95% CI** | ***p*FDR** |
| --- | --- | --- | --- | --- | --- | --- |
| Anterior thalamus | MA : RES | 0.08 | [-0.09, 0.24] | 1.08 | [0.91, 1.27] | 0.384 |
|  | VU : RES | 0.11 | [-0.03, 0.25] | 1.12 | [0.97, 1.29] | 0.504 |
|  | HC : RES | 0.07 | [-0.05, 0.2] | 1.08 | [0.95, 1.22] | 0.637 |
| Cingulate cingulum | MA : RES | 0.09 | [-0.07, 0.26] | 1.10 | [0.93, 1.29] | 0.384 |
|  | VU : RES | 0.10 | [-0.04, 0.24] | 1.10 | [0.96, 1.27] | 0.504 |
|  | HC : RES | 0.07 | [-0.05, 0.2] | 1.07 | [0.95, 1.22] | 0.637 |
| Corpus callosum | MA : RES | 0.11 | [-0.06, 0.28] | 1.12 | [0.95, 1.32] | 0.384 |
|  | VU : RES | 0.14 | [0, 0.28] | 1.15 | [1, 1.32] | 0.504 |
|  | HC : RES | 0.10 | [-0.02, 0.23] | 1.11 | [0.98, 1.26] | 0.637 |
| Corticospinal | MA : RES | 0.03 | [-0.13, 0.2] | 1.04 | [0.88, 1.22] | 0.682 |
|  | VU : RES | 0.01 | [-0.13, 0.16] | 1.01 | [0.88, 1.17] | 0.851 |
|  | HC : RES | 0.01 | [-0.11, 0.14] | 1.01 | [0.89, 1.15] | 0.908 |
| Forceps major | MA : RES | 0.11 | [-0.06, 0.27] | 1.11 | [0.94, 1.31] | 0.384 |
|  | VU : RES | 0.22 | [0.07, 0.36] | 1.24 | [1.08, 1.43] | 0.057 |
|  | HC : RES | 0.13 | [0, 0.25] | 1.14 | [1, 1.29] | 0.637 |
| Forceps minor | MA : RES | 0.09 | [-0.07, 0.26] | 1.10 | [0.93, 1.29] | 0.384 |
|  | VU : RES | 0.07 | [-0.07, 0.22] | 1.08 | [0.94, 1.24] | 0.504 |
|  | HC : RES | 0.08 | [-0.04, 0.21] | 1.08 | [0.96, 1.23] | 0.637 |
| Fornix | MA : RES | 0.10 | [-0.07, 0.26] | 1.10 | [0.93, 1.3] | 0.384 |
|  | VU : RES | 0.07 | [-0.07, 0.21] | 1.07 | [0.93, 1.24] | 0.504 |
|  | HC : RES | 0.09 | [-0.04, 0.21] | 1.09 | [0.96, 1.23] | 0.637 |
| Inferior longitudinal fasiculus | MA : RES | 0.12 | [-0.05, 0.29] | 1.13 | [0.95, 1.33] | 0.384 |
|  | VU : RES | 0.09 | [-0.06, 0.23] | 1.09 | [0.95, 1.26] | 0.504 |
|  | HC : RES | 0.02 | [-0.1, 0.15] | 1.02 | [0.9, 1.16] | 0.865 |
| Inferior frontal superior frontal cortex | MA : RES | 0.08 | [-0.09, 0.25] | 1.08 | [0.92, 1.28] | 0.384 |
|  | VU : RES | 0.07 | [-0.07, 0.21] | 1.07 | [0.93, 1.24] | 0.504 |
|  | HC : RES | 0.00 | [-0.12, 0.13] | 1.00 | [0.89, 1.14] | 0.954 |
| Inferior fronto-occipital fasiculus | MA : RES | 0.15 | [-0.02, 0.31] | 1.16 | [0.98, 1.37] | 0.384 |
|  | VU : RES | 0.11 | [-0.03, 0.25] | 1.12 | [0.97, 1.29] | 0.504 |
|  | HC : RES | 0.08 | [-0.04, 0.21] | 1.08 | [0.96, 1.23] | 0.637 |
| Parahippocampal cingulum | MA : RES | 0.11 | [-0.06, 0.27] | 1.11 | [0.94, 1.31] | 0.384 |
|  | VU : RES | 0.05 | [-0.09, 0.19] | 1.05 | [0.91, 1.21] | 0.669 |
|  | HC : RES | 0.01 | [-0.11, 0.13] | 1.01 | [0.89, 1.14] | 0.908 |
| Parietal superior longitudinal fasiculus | MA : RES | 0.10 | [-0.06, 0.27] | 1.11 | [0.94, 1.31] | 0.384 |
|  | VU : RES | 0.07 | [-0.07, 0.22] | 1.08 | [0.93, 1.24] | 0.504 |
|  | HC : RES | 0.06 | [-0.07, 0.18] | 1.06 | [0.93, 1.2] | 0.700 |
| Striatal inferior frontal cortex | MA : RES | 0.12 | [-0.05, 0.29] | 1.13 | [0.96, 1.33] | 0.384 |
|  | VU : RES | 0.10 | [-0.05, 0.24] | 1.10 | [0.95, 1.27] | 0.504 |
|  | HC : RES | 0.06 | [-0.06, 0.18] | 1.06 | [0.94, 1.2] | 0.700 |
| Superior corticostriate-frontal cortex | MA : RES | 0.08 | [-0.09, 0.25] | 1.08 | [0.92, 1.28] | 0.384 |
|  | VU : RES | 0.02 | [-0.12, 0.16] | 1.02 | [0.88, 1.18] | 0.851 |
|  | HC : RES | -0.05 | [-0.17, 0.08] | 0.95 | [0.84, 1.08] | 0.786 |
| Superior corticostriate-parietal cortex | MA : RES | 0.08 | [-0.09, 0.25] | 1.08 | [0.92, 1.28] | 0.384 |
|  | VU : RES | 0.02 | [-0.13, 0.16] | 1.02 | [0.88, 1.17] | 0.851 |
|  | HC : RES | -0.03 | [-0.15, 0.1] | 0.98 | [0.86, 1.11] | 0.865 |
| Superior corticostriatum | MA : RES | 0.08 | [-0.09, 0.25] | 1.09 | [0.92, 1.28] | 0.384 |
|  | VU : RES | 0.02 | [-0.12, 0.17] | 1.02 | [0.89, 1.18] | 0.851 |
|  | HC : RES | -0.03 | [-0.16, 0.09] | 0.97 | [0.85, 1.1] | 0.811 |
| Superior longitudinal fasiculus | MA : RES | 0.11 | [-0.06, 0.28] | 1.12 | [0.95, 1.32] | 0.384 |
|  | VU : RES | 0.08 | [-0.06, 0.22] | 1.08 | [0.94, 1.25] | 0.504 |
|  | HC : RES | 0.04 | [-0.09, 0.16] | 1.04 | [0.92, 1.18] | 0.811 |
| Temporal superior longitudinal fasiculus | MA : RES | 0.12 | [-0.05, 0.29] | 1.13 | [0.96, 1.33] | 0.384 |
|  | VU : RES | 0.08 | [-0.06, 0.22] | 1.09 | [0.94, 1.25] | 0.504 |
|  | HC : RES | 0.03 | [-0.09, 0.16] | 1.03 | [0.91, 1.17] | 0.811 |
| Uncinate | MA : RES | 0.10 | [-0.06, 0.27] | 1.11 | [0.94, 1.31] | 0.384 |
|  | VU : RES | 0.04 | [-0.1, 0.18] | 1.04 | [0.9, 1.2] | 0.737 |
|  | HC : RES | 0.08 | [-0.04, 0.21] | 1.09 | [0.96, 1.23] | 0.637 |

### Table S9. Externalizing group comparisons

| **Variables** | **Group comparisons** | **B** | **95% CI** | **Odds** | **95% CI** | ***p*FDR** |
| --- | --- | --- | --- | --- | --- | --- |
| Anterior thalamus | MA : RES | -0.28 | [-0.7, 0.13] | 0.75 | [0.5, 1.14] | 0.373 |
|  | VU : RES | 0.30 | [-0.13, 0.72] | 1.34 | [0.88, 2.05] | 0.399 |
|  | HC : RES | 0.05 | [-0.05, 0.15] | 1.06 | [0.95, 1.17] | 0.939 |
| Cingulate cingulum | MA : RES | -0.47 | [-0.87, -0.08] | 0.62 | [0.42, 0.93] | 0.351 |
|  | VU : RES | 0.22 | [-0.19, 0.63] | 1.25 | [0.82, 1.89] | 0.437 |
|  | HC : RES | 0.01 | [-0.09, 0.11] | 1.01 | [0.91, 1.12] | 0.939 |
| Corpus callosum | MA : RES | -0.36 | [-0.79, 0.07] | 0.70 | [0.46, 1.08] | 0.373 |
|  | VU : RES | 0.35 | [-0.07, 0.77] | 1.42 | [0.93, 2.15] | 0.399 |
|  | HC : RES | 0.06 | [-0.04, 0.16] | 1.06 | [0.96, 1.18] | 0.939 |
| Corticospinal | MA : RES | -0.11 | [-0.51, 0.29] | 0.90 | [0.6, 1.34] | 0.645 |
|  | VU : RES | 0.16 | [-0.26, 0.58] | 1.18 | [0.77, 1.79] | 0.517 |
|  | HC : RES | 0.02 | [-0.08, 0.12] | 1.02 | [0.92, 1.13] | 0.939 |
| Forceps major | MA : RES | -0.31 | [-0.73, 0.12] | 0.74 | [0.48, 1.13] | 0.373 |
|  | VU : RES | 0.42 | [0.01, 0.83] | 1.52 | [1.01, 2.29] | 0.399 |
|  | HC : RES | 0.10 | [0, 0.2] | 1.11 | [1, 1.23] | 0.924 |
| Forceps minor | MA : RES | -0.29 | [-0.71, 0.13] | 0.75 | [0.49, 1.13] | 0.373 |
|  | VU : RES | 0.25 | [-0.17, 0.66] | 1.28 | [0.85, 1.93] | 0.420 |
|  | HC : RES | 0.03 | [-0.07, 0.13] | 1.03 | [0.93, 1.14] | 0.939 |
| Fornix | MA : RES | -0.11 | [-0.53, 0.31] | 0.90 | [0.59, 1.37] | 0.645 |
|  | VU : RES | 0.04 | [-0.38, 0.47] | 1.04 | [0.68, 1.6] | 0.846 |
|  | HC : RES | 0.05 | [-0.05, 0.15] | 1.05 | [0.95, 1.16] | 0.939 |
| Inferior longitudinal fasiculus | MA : RES | -0.19 | [-0.61, 0.23] | 0.83 | [0.54, 1.26] | 0.559 |
|  | VU : RES | 0.34 | [-0.09, 0.77] | 1.40 | [0.91, 2.16] | 0.399 |
|  | HC : RES | -0.02 | [-0.12, 0.08] | 0.98 | [0.89, 1.08] | 0.939 |
| Inferior frontal superior frontal cortex | MA : RES | -0.42 | [-0.83, -0.01] | 0.66 | [0.43, 0.99] | 0.351 |
|  | VU : RES | 0.27 | [-0.14, 0.68] | 1.31 | [0.87, 1.98] | 0.399 |
|  | HC : RES | -0.01 | [-0.11, 0.1] | 0.99 | [0.9, 1.1] | 0.939 |
| Inferior fronto-occipital fasiculus | MA : RES | -0.21 | [-0.64, 0.21] | 0.81 | [0.53, 1.23] | 0.557 |
|  | VU : RES | 0.39 | [-0.03, 0.82] | 1.48 | [0.97, 2.27] | 0.399 |
|  | HC : RES | 0.03 | [-0.07, 0.13] | 1.03 | [0.93, 1.14] | 0.939 |
| Parahippocampal cingulum | MA : RES | 0.02 | [-0.4, 0.45] | 1.02 | [0.67, 1.56] | 0.922 |
|  | VU : RES | 0.29 | [-0.14, 0.72] | 1.34 | [0.87, 2.06] | 0.399 |
|  | HC : RES | 0.00 | [-0.1, 0.1] | 1.00 | [0.91, 1.11] | 0.939 |
| Parietal superior longitudinal fasiculus | MA : RES | -0.12 | [-0.52, 0.28] | 0.89 | [0.59, 1.33] | 0.645 |
|  | VU : RES | 0.26 | [-0.15, 0.67] | 1.30 | [0.86, 1.95] | 0.399 |
|  | HC : RES | 0.03 | [-0.08, 0.13] | 1.03 | [0.93, 1.14] | 0.939 |
| Striatal inferior frontal cortex | MA : RES | -0.12 | [-0.54, 0.29] | 0.88 | [0.58, 1.34] | 0.645 |
|  | VU : RES | 0.23 | [-0.18, 0.64] | 1.26 | [0.84, 1.9] | 0.426 |
|  | HC : RES | 0.02 | [-0.08, 0.12] | 1.02 | [0.92, 1.13] | 0.939 |
| Superior corticostriate-frontal cortex | MA : RES | -0.39 | [-0.8, 0.01] | 0.68 | [0.45, 1.01] | 0.351 |
|  | VU : RES | 0.14 | [-0.27, 0.56] | 1.16 | [0.77, 1.74] | 0.517 |
|  | HC : RES | -0.05 | [-0.16, 0.05] | 0.95 | [0.86, 1.05] | 0.939 |
| Superior corticostriate-parietal cortex | MA : RES | -0.23 | [-0.63, 0.18] | 0.80 | [0.53, 1.2] | 0.520 |
|  | VU : RES | 0.15 | [-0.26, 0.56] | 1.16 | [0.77, 1.75] | 0.517 |
|  | HC : RES | -0.02 | [-0.12, 0.09] | 0.98 | [0.89, 1.09] | 0.939 |
| Superior corticostriatum | MA : RES | -0.31 | [-0.72, 0.1] | 0.73 | [0.49, 1.11] | 0.373 |
|  | VU : RES | 0.15 | [-0.27, 0.56] | 1.16 | [0.77, 1.75] | 0.517 |
|  | HC : RES | -0.03 | [-0.13, 0.07] | 0.97 | [0.88, 1.08] | 0.939 |
| Superior longitudinal fasiculus | MA : RES | -0.15 | [-0.56, 0.25] | 0.86 | [0.57, 1.29] | 0.619 |
|  | VU : RES | 0.29 | [-0.12, 0.69] | 1.33 | [0.89, 1.99] | 0.399 |
|  | HC : RES | 0.01 | [-0.09, 0.11] | 1.01 | [0.92, 1.12] | 0.939 |
| Temporal superior longitudinal fasiculus | MA : RES | -0.18 | [-0.6, 0.23] | 0.83 | [0.55, 1.26] | 0.559 |
|  | VU : RES | 0.30 | [-0.1, 0.7] | 1.35 | [0.9, 2.02] | 0.399 |
|  | HC : RES | 0.01 | [-0.09, 0.11] | 1.01 | [0.91, 1.11] | 0.939 |
| Uncinate | MA : RES | -0.38 | [-0.79, 0.04] | 0.69 | [0.45, 1.04] | 0.351 |
|  | VU : RES | 0.20 | [-0.22, 0.61] | 1.22 | [0.8, 1.85] | 0.488 |
|  | HC : RES | 0.02 | [-0.08, 0.13] | 1.02 | [0.93, 1.13] | 0.939 |

# Appendix S9. Full sample results – Resting-state functional connectivity

## Main effects

### Table S10. Internalizing group comparisons

| **Variables** | **Group comparisons** | **B** | **95% CI** | **Odds** | **95% CI** | ***p*FDR** |
| --- | --- | --- | --- | --- | --- | --- |
| AN-AN | MA : RES | -0.07 | [-0.22, 0.08] | 0.94 | [0.8, 1.09] | 0.917 |
|  | VU : RES | -0.02 | [-0.15, 0.1] | 0.98 | [0.86, 1.11] | 0.879 |
|  | HC : RES | 0.10 | [0.02, 0.18] | 1.10 | [1.02, 1.19] | 0.249 |
| AN-Amygdala | MA : RES | 0.04 | [-0.11, 0.19] | 1.04 | [0.9, 1.21] | 0.917 |
|  | VU : RES | -0.01 | [-0.14, 0.11] | 0.99 | [0.87, 1.12] | 0.959 |
|  | HC : RES | -0.01 | [-0.09, 0.07] | 0.99 | [0.91, 1.07] | 0.929 |
| AN-CPN | MA : RES | 0.04 | [-0.1, 0.19] | 1.05 | [0.9, 1.21] | 0.917 |
|  | VU : RES | 0.05 | [-0.08, 0.18] | 1.05 | [0.93, 1.19] | 0.820 |
|  | HC : RES | -0.07 | [-0.15, 0.01] | 0.94 | [0.86, 1.01] | 0.387 |
| AN-CON | MA : RES | -0.05 | [-0.2, 0.1] | 0.95 | [0.82, 1.11] | 0.917 |
|  | VU : RES | 0.00 | [-0.13, 0.12] | 1.00 | [0.88, 1.13] | 0.987 |
|  | HC : RES | 0.07 | [-0.01, 0.15] | 1.07 | [0.99, 1.16] | 0.339 |
| AN-DAN | MA : RES | -0.08 | [-0.22, 0.06] | 0.92 | [0.8, 1.07] | 0.879 |
|  | VU : RES | -0.10 | [-0.22, 0.03] | 0.91 | [0.8, 1.03] | 0.562 |
|  | HC : RES | -0.11 | [-0.19, -0.03] | 0.90 | [0.83, 0.97] | 0.153 |
| AN-DMN | MA : RES | 0.13 | [-0.02, 0.28] | 1.13 | [0.98, 1.32] | 0.513 |
|  | VU : RES | 0.13 | [0, 0.25] | 1.13 | [1, 1.29] | 0.425 |
|  | HC : RES | 0.07 | [-0.01, 0.15] | 1.07 | [0.99, 1.16] | 0.377 |
| AN-FPN | MA : RES | 0.03 | [-0.11, 0.18] | 1.03 | [0.89, 1.2] | 0.917 |
|  | VU : RES | 0.07 | [-0.05, 0.2] | 1.07 | [0.95, 1.22] | 0.651 |
|  | HC : RES | -0.05 | [-0.13, 0.03] | 0.95 | [0.87, 1.03] | 0.484 |
| AN-hippocampus | MA : RES | -0.02 | [-0.17, 0.12] | 0.98 | [0.85, 1.13] | 0.918 |
|  | VU : RES | -0.07 | [-0.19, 0.06] | 0.93 | [0.82, 1.06] | 0.651 |
|  | HC : RES | -0.01 | [-0.09, 0.07] | 0.99 | [0.92, 1.08] | 0.962 |
| AN-RTN | MA : RES | 0.04 | [-0.11, 0.19] | 1.04 | [0.9, 1.21] | 0.917 |
|  | VU : RES | 0.14 | [0.01, 0.26] | 1.15 | [1.01, 1.3] | 0.346 |
|  | HC : RES | -0.03 | [-0.11, 0.05] | 0.97 | [0.9, 1.05] | 0.726 |
| AN-SN | MA : RES | 0.16 | [0.01, 0.3] | 1.17 | [1.01, 1.35] | 0.513 |
|  | VU : RES | 0.07 | [-0.05, 0.2] | 1.08 | [0.95, 1.22] | 0.651 |
|  | HC : RES | 0.07 | [-0.01, 0.15] | 1.08 | [0.99, 1.16] | 0.339 |
| AN-SMN(H) | MA : RES | -0.15 | [-0.3, 0] | 0.86 | [0.74, 1] | 0.513 |
|  | VU : RES | -0.05 | [-0.18, 0.07] | 0.95 | [0.83, 1.08] | 0.803 |
|  | HC : RES | 0.05 | [-0.03, 0.13] | 1.05 | [0.97, 1.14] | 0.538 |
| AN-SMN(M) | MA : RES | -0.06 | [-0.21, 0.08] | 0.94 | [0.81, 1.09] | 0.917 |
|  | VU : RES | -0.01 | [-0.14, 0.12] | 0.99 | [0.87, 1.12] | 0.968 |
|  | HC : RES | 0.05 | [-0.03, 0.13] | 1.05 | [0.97, 1.14] | 0.537 |
| AN-VN | MA : RES | 0.04 | [-0.11, 0.18] | 1.04 | [0.9, 1.2] | 0.917 |
|  | VU : RES | 0.01 | [-0.12, 0.13] | 1.01 | [0.89, 1.14] | 0.968 |
|  | HC : RES | -0.08 | [-0.16, 0] | 0.92 | [0.85, 1] | 0.304 |
| AN-VAN | MA : RES | 0.04 | [-0.11, 0.19] | 1.04 | [0.89, 1.21] | 0.917 |
|  | VU : RES | 0.06 | [-0.07, 0.18] | 1.06 | [0.93, 1.2] | 0.767 |
|  | HC : RES | 0.05 | [-0.03, 0.13] | 1.05 | [0.97, 1.14] | 0.537 |
| CPN-Amygdala | MA : RES | -0.13 | [-0.27, 0.02] | 0.88 | [0.76, 1.02] | 0.513 |
|  | VU : RES | -0.06 | [-0.19, 0.06] | 0.94 | [0.83, 1.06] | 0.691 |
|  | HC : RES | -0.07 | [-0.15, 0.01] | 0.93 | [0.86, 1.01] | 0.339 |
| CPN-CPN | MA : RES | 0.07 | [-0.08, 0.21] | 1.07 | [0.92, 1.24] | 0.917 |
|  | VU : RES | 0.01 | [-0.12, 0.13] | 1.01 | [0.89, 1.14] | 0.968 |
|  | HC : RES | -0.05 | [-0.13, 0.03] | 0.95 | [0.88, 1.03] | 0.537 |
| CPN-CON | MA : RES | 0.04 | [-0.11, 0.19] | 1.04 | [0.9, 1.21] | 0.917 |
|  | VU : RES | 0.09 | [-0.04, 0.22] | 1.09 | [0.97, 1.24] | 0.562 |
|  | HC : RES | -0.03 | [-0.11, 0.05] | 0.97 | [0.9, 1.05] | 0.726 |
| CPN-DAN | MA : RES | 0.14 | [-0.01, 0.29] | 1.15 | [0.99, 1.33] | 0.513 |
|  | VU : RES | 0.10 | [-0.03, 0.22] | 1.10 | [0.97, 1.25] | 0.562 |
|  | HC : RES | 0.05 | [-0.03, 0.13] | 1.05 | [0.97, 1.13] | 0.565 |
| CPN-DMN | MA : RES | -0.03 | [-0.17, 0.12] | 0.97 | [0.84, 1.13] | 0.918 |
|  | VU : RES | -0.09 | [-0.21, 0.04] | 0.92 | [0.81, 1.04] | 0.567 |
|  | HC : RES | -0.02 | [-0.1, 0.06] | 0.98 | [0.91, 1.06] | 0.868 |
| CPN-FPN | MA : RES | -0.01 | [-0.15, 0.14] | 0.99 | [0.86, 1.15] | 0.973 |
|  | VU : RES | 0.04 | [-0.09, 0.16] | 1.04 | [0.92, 1.18] | 0.820 |
|  | HC : RES | 0.00 | [-0.08, 0.08] | 1.00 | [0.93, 1.08] | 0.979 |
| CPN-Hippocampus | MA : RES | -0.04 | [-0.2, 0.11] | 0.96 | [0.82, 1.11] | 0.917 |
|  | VU : RES | -0.05 | [-0.18, 0.08] | 0.95 | [0.84, 1.08] | 0.820 |
|  | HC : RES | 0.00 | [-0.08, 0.09] | 1.00 | [0.93, 1.09] | 0.972 |
| CPN-RTN | MA : RES | 0.05 | [-0.1, 0.19] | 1.05 | [0.9, 1.21] | 0.917 |
|  | VU : RES | 0.02 | [-0.1, 0.15] | 1.02 | [0.9, 1.16] | 0.879 |
|  | HC : RES | 0.11 | [0.03, 0.19] | 1.12 | [1.03, 1.21] | 0.153 |
| CPN-SN | MA : RES | -0.01 | [-0.16, 0.13] | 0.99 | [0.85, 1.14] | 0.949 |
|  | VU : RES | 0.16 | [0.03, 0.28] | 1.17 | [1.03, 1.33] | 0.265 |
|  | HC : RES | -0.03 | [-0.11, 0.05] | 0.97 | [0.9, 1.05] | 0.726 |
| CPN-SMN(H) | MA : RES | 0.06 | [-0.08, 0.2] | 1.06 | [0.92, 1.23] | 0.917 |
|  | VU : RES | 0.07 | [-0.06, 0.19] | 1.07 | [0.94, 1.21] | 0.651 |
|  | HC : RES | 0.03 | [-0.05, 0.11] | 1.03 | [0.95, 1.11] | 0.726 |
| CPN-SMN(M) | MA : RES | -0.10 | [-0.24, 0.04] | 0.90 | [0.78, 1.04] | 0.616 |
|  | VU : RES | 0.01 | [-0.12, 0.13] | 1.01 | [0.89, 1.14] | 0.968 |
|  | HC : RES | -0.04 | [-0.12, 0.04] | 0.96 | [0.89, 1.04] | 0.609 |
| CPN-VN | MA : RES | -0.02 | [-0.16, 0.13] | 0.98 | [0.85, 1.14] | 0.931 |
|  | VU : RES | 0.03 | [-0.09, 0.15] | 1.03 | [0.91, 1.17] | 0.858 |
|  | HC : RES | 0.03 | [-0.05, 0.11] | 1.03 | [0.95, 1.12] | 0.726 |
| CPN-VAN | MA : RES | -0.12 | [-0.26, 0.03] | 0.89 | [0.77, 1.03] | 0.558 |
|  | VU : RES | -0.14 | [-0.27, -0.02] | 0.87 | [0.77, 0.98] | 0.302 |
|  | HC : RES | -0.05 | [-0.13, 0.02] | 0.95 | [0.87, 1.02] | 0.484 |
| CON-Amygdala | MA : RES | 0.14 | [-0.01, 0.28] | 1.14 | [0.99, 1.33] | 0.513 |
|  | VU : RES | 0.07 | [-0.05, 0.2] | 1.07 | [0.95, 1.22] | 0.651 |
|  | HC : RES | 0.04 | [-0.04, 0.12] | 1.04 | [0.96, 1.13] | 0.610 |
| CON-CON | MA : RES | -0.14 | [-0.3, 0.01] | 0.87 | [0.74, 1.01] | 0.513 |
|  | VU : RES | -0.04 | [-0.17, 0.09] | 0.96 | [0.84, 1.09] | 0.820 |
|  | HC : RES | 0.07 | [-0.01, 0.15] | 1.07 | [0.99, 1.17] | 0.339 |
| CON-DAN | MA : RES | -0.14 | [-0.29, 0.01] | 0.87 | [0.75, 1.01] | 0.513 |
|  | VU : RES | 0.00 | [-0.12, 0.13] | 1.00 | [0.89, 1.13] | 0.987 |
|  | HC : RES | 0.00 | [-0.08, 0.08] | 1.00 | [0.92, 1.08] | 0.972 |
| CON-DMN | MA : RES | 0.20 | [0.05, 0.35] | 1.22 | [1.05, 1.42] | 0.513 |
|  | VU : RES | 0.11 | [-0.02, 0.24] | 1.12 | [0.98, 1.27] | 0.497 |
|  | HC : RES | -0.01 | [-0.09, 0.07] | 0.99 | [0.91, 1.07] | 0.929 |
| CON-FPN | MA : RES | -0.01 | [-0.16, 0.14] | 0.99 | [0.85, 1.15] | 0.968 |
|  | VU : RES | 0.03 | [-0.1, 0.15] | 1.03 | [0.91, 1.16] | 0.879 |
|  | HC : RES | -0.07 | [-0.15, 0.01] | 0.93 | [0.86, 1.01] | 0.339 |
| CON-Hippocampus | MA : RES | 0.04 | [-0.11, 0.19] | 1.04 | [0.89, 1.2] | 0.917 |
|  | VU : RES | 0.12 | [-0.02, 0.25] | 1.12 | [0.98, 1.28] | 0.497 |
|  | HC : RES | 0.06 | [-0.02, 0.15] | 1.07 | [0.98, 1.16] | 0.415 |
| CON-RTN | MA : RES | 0.15 | [0, 0.3] | 1.17 | [1, 1.36] | 0.513 |
|  | VU : RES | 0.09 | [-0.04, 0.22] | 1.10 | [0.96, 1.25] | 0.562 |
|  | HC : RES | -0.01 | [-0.09, 0.07] | 0.99 | [0.91, 1.08] | 0.945 |
| CON-SN | MA : RES | 0.13 | [-0.02, 0.27] | 1.14 | [0.98, 1.31] | 0.513 |
|  | VU : RES | 0.05 | [-0.08, 0.17] | 1.05 | [0.93, 1.19] | 0.820 |
|  | HC : RES | 0.08 | [0.01, 0.16] | 1.09 | [1.01, 1.18] | 0.266 |
| CON-SMN(H) | MA : RES | -0.11 | [-0.26, 0.04] | 0.90 | [0.77, 1.04] | 0.616 |
|  | VU : RES | 0.03 | [-0.09, 0.16] | 1.03 | [0.91, 1.17] | 0.858 |
|  | HC : RES | 0.07 | [-0.01, 0.15] | 1.07 | [0.99, 1.16] | 0.339 |
| CON-SMN(M) | MA : RES | -0.07 | [-0.21, 0.08] | 0.94 | [0.81, 1.09] | 0.917 |
|  | VU : RES | 0.00 | [-0.12, 0.13] | 1.00 | [0.88, 1.14] | 0.987 |
|  | HC : RES | 0.06 | [-0.02, 0.14] | 1.06 | [0.98, 1.15] | 0.468 |
| CON-VN | MA : RES | 0.00 | [-0.14, 0.15] | 1.00 | [0.87, 1.16] | 0.988 |
|  | VU : RES | 0.01 | [-0.12, 0.13] | 1.01 | [0.89, 1.14] | 0.968 |
|  | HC : RES | -0.01 | [-0.09, 0.07] | 0.99 | [0.92, 1.07] | 0.929 |
| CON-VAN | MA : RES | 0.03 | [-0.12, 0.19] | 1.04 | [0.89, 1.2] | 0.917 |
|  | VU : RES | 0.04 | [-0.09, 0.17] | 1.04 | [0.92, 1.18] | 0.820 |
|  | HC : RES | -0.03 | [-0.11, 0.05] | 0.97 | [0.89, 1.05] | 0.726 |
| DAN-Amygdala | MA : RES | 0.02 | [-0.13, 0.16] | 1.02 | [0.88, 1.18] | 0.931 |
|  | VU : RES | 0.09 | [-0.04, 0.21] | 1.09 | [0.96, 1.23] | 0.568 |
|  | HC : RES | -0.03 | [-0.11, 0.05] | 0.97 | [0.9, 1.05] | 0.726 |
| DAN-DAN | MA : RES | -0.04 | [-0.19, 0.1] | 0.96 | [0.83, 1.11] | 0.917 |
|  | VU : RES | 0.06 | [-0.07, 0.18] | 1.06 | [0.94, 1.2] | 0.746 |
|  | HC : RES | 0.06 | [-0.02, 0.14] | 1.06 | [0.98, 1.15] | 0.436 |
| DAN-DMN | MA : RES | 0.06 | [-0.1, 0.21] | 1.06 | [0.91, 1.24] | 0.917 |
|  | VU : RES | -0.05 | [-0.18, 0.08] | 0.95 | [0.83, 1.08] | 0.820 |
|  | HC : RES | -0.07 | [-0.16, 0.01] | 0.93 | [0.86, 1.01] | 0.339 |
| DAN-FPN | MA : RES | 0.04 | [-0.1, 0.19] | 1.05 | [0.9, 1.21] | 0.917 |
|  | VU : RES | 0.02 | [-0.1, 0.15] | 1.03 | [0.9, 1.16] | 0.879 |
|  | HC : RES | 0.04 | [-0.04, 0.12] | 1.04 | [0.96, 1.12] | 0.683 |
| DAN-Hippocampus | MA : RES | -0.08 | [-0.23, 0.07] | 0.92 | [0.8, 1.07] | 0.902 |
|  | VU : RES | 0.04 | [-0.09, 0.16] | 1.04 | [0.91, 1.18] | 0.820 |
|  | HC : RES | -0.04 | [-0.12, 0.04] | 0.96 | [0.89, 1.04] | 0.664 |
| DAN-RTN | MA : RES | -0.05 | [-0.19, 0.1] | 0.96 | [0.82, 1.11] | 0.917 |
|  | VU : RES | -0.01 | [-0.13, 0.12] | 0.99 | [0.88, 1.13] | 0.968 |
|  | HC : RES | 0.03 | [-0.05, 0.11] | 1.03 | [0.95, 1.11] | 0.726 |
| DAN-SN | MA : RES | -0.05 | [-0.19, 0.1] | 0.96 | [0.83, 1.11] | 0.917 |
|  | VU : RES | -0.04 | [-0.17, 0.08] | 0.96 | [0.85, 1.08] | 0.820 |
|  | HC : RES | -0.04 | [-0.12, 0.04] | 0.96 | [0.89, 1.04] | 0.609 |
| DAN-SMN(H) | MA : RES | -0.07 | [-0.21, 0.07] | 0.93 | [0.81, 1.07] | 0.917 |
|  | VU : RES | -0.03 | [-0.15, 0.1] | 0.98 | [0.86, 1.1] | 0.879 |
|  | HC : RES | -0.04 | [-0.12, 0.03] | 0.96 | [0.89, 1.03] | 0.596 |
| DAN-SMN(M) | MA : RES | 0.01 | [-0.13, 0.16] | 1.01 | [0.88, 1.17] | 0.949 |
|  | VU : RES | 0.04 | [-0.09, 0.16] | 1.04 | [0.92, 1.18] | 0.820 |
|  | HC : RES | -0.01 | [-0.09, 0.07] | 0.99 | [0.91, 1.07] | 0.929 |
| DAN-VN | MA : RES | -0.03 | [-0.18, 0.11] | 0.97 | [0.83, 1.12] | 0.917 |
|  | VU : RES | 0.02 | [-0.1, 0.15] | 1.02 | [0.9, 1.16] | 0.879 |
|  | HC : RES | 0.04 | [-0.04, 0.12] | 1.04 | [0.96, 1.13] | 0.664 |
| DAN-VAN | MA : RES | -0.07 | [-0.22, 0.08] | 0.94 | [0.8, 1.09] | 0.917 |
|  | VU : RES | -0.15 | [-0.28, -0.02] | 0.86 | [0.76, 0.98] | 0.302 |
|  | HC : RES | -0.13 | [-0.21, -0.04] | 0.88 | [0.81, 0.96] | 0.153 |
| DMN-Amygdala | MA : RES | -0.03 | [-0.18, 0.11] | 0.97 | [0.84, 1.12] | 0.917 |
|  | VU : RES | 0.04 | [-0.08, 0.17] | 1.04 | [0.92, 1.18] | 0.820 |
|  | HC : RES | -0.02 | [-0.1, 0.06] | 0.98 | [0.91, 1.06] | 0.829 |
| DMN-DMN | MA : RES | -0.14 | [-0.29, 0.01] | 0.87 | [0.75, 1.01] | 0.513 |
|  | VU : RES | -0.07 | [-0.2, 0.06] | 0.93 | [0.82, 1.06] | 0.651 |
|  | HC : RES | 0.03 | [-0.05, 0.11] | 1.03 | [0.95, 1.12] | 0.726 |
| DMN-FPN | MA : RES | 0.02 | [-0.13, 0.16] | 1.02 | [0.88, 1.18] | 0.931 |
|  | VU : RES | -0.02 | [-0.15, 0.1] | 0.98 | [0.86, 1.11] | 0.889 |
|  | HC : RES | -0.01 | [-0.09, 0.07] | 0.99 | [0.91, 1.07] | 0.929 |
| DMN-Hippocampus | MA : RES | 0.11 | [-0.04, 0.26] | 1.12 | [0.96, 1.3] | 0.588 |
|  | VU : RES | 0.14 | [0.01, 0.27] | 1.15 | [1.01, 1.31] | 0.302 |
|  | HC : RES | 0.10 | [0.01, 0.18] | 1.10 | [1.01, 1.19] | 0.249 |
| DMN-RTN | MA : RES | -0.02 | [-0.17, 0.13] | 0.98 | [0.84, 1.13] | 0.918 |
|  | VU : RES | -0.10 | [-0.22, 0.03] | 0.91 | [0.8, 1.03] | 0.562 |
|  | HC : RES | -0.03 | [-0.11, 0.05] | 0.97 | [0.9, 1.05] | 0.726 |
| DMN-SN | MA : RES | 0.00 | [-0.15, 0.14] | 1.00 | [0.86, 1.15] | 0.985 |
|  | VU : RES | 0.11 | [-0.02, 0.23] | 1.11 | [0.98, 1.26] | 0.497 |
|  | HC : RES | -0.01 | [-0.09, 0.07] | 0.99 | [0.91, 1.07] | 0.929 |
| DMN-SMN(H) | MA : RES | 0.13 | [-0.02, 0.28] | 1.14 | [0.98, 1.32] | 0.513 |
|  | VU : RES | 0.01 | [-0.12, 0.13] | 1.01 | [0.89, 1.14] | 0.968 |
|  | HC : RES | 0.03 | [-0.05, 0.11] | 1.03 | [0.95, 1.11] | 0.732 |
| DMN-SMN(M) | MA : RES | 0.11 | [-0.04, 0.25] | 1.11 | [0.96, 1.29] | 0.616 |
|  | VU : RES | -0.02 | [-0.14, 0.11] | 0.98 | [0.87, 1.12] | 0.929 |
|  | HC : RES | -0.04 | [-0.12, 0.04] | 0.96 | [0.89, 1.04] | 0.664 |
| DMN-VN | MA : RES | 0.03 | [-0.12, 0.18] | 1.03 | [0.89, 1.2] | 0.917 |
|  | VU : RES | 0.07 | [-0.06, 0.2] | 1.07 | [0.94, 1.22] | 0.651 |
|  | HC : RES | -0.04 | [-0.12, 0.04] | 0.96 | [0.88, 1.04] | 0.609 |
| DMN-VAN | MA : RES | 0.06 | [-0.09, 0.21] | 1.06 | [0.91, 1.23] | 0.917 |
|  | VU : RES | 0.07 | [-0.06, 0.19] | 1.07 | [0.94, 1.21] | 0.651 |
|  | HC : RES | 0.08 | [0, 0.16] | 1.08 | [1, 1.17] | 0.318 |
| FPN-Amygdala | MA : RES | 0.23 | [0.08, 0.38] | 1.26 | [1.08, 1.46] | 0.286 |
|  | VU : RES | 0.18 | [0.06, 0.31] | 1.20 | [1.06, 1.36] | 0.246 |
|  | HC : RES | 0.12 | [0.04, 0.2] | 1.13 | [1.04, 1.22] | 0.153 |
| FPN-FPN | MA : RES | 0.02 | [-0.12, 0.17] | 1.02 | [0.88, 1.18] | 0.918 |
|  | VU : RES | -0.09 | [-0.21, 0.04] | 0.92 | [0.81, 1.04] | 0.567 |
|  | HC : RES | 0.01 | [-0.07, 0.09] | 1.01 | [0.93, 1.09] | 0.929 |
| FPN-Hippocampus | MA : RES | 0.10 | [-0.05, 0.25] | 1.11 | [0.95, 1.29] | 0.622 |
|  | VU : RES | 0.09 | [-0.03, 0.22] | 1.10 | [0.97, 1.24] | 0.562 |
|  | HC : RES | 0.09 | [0.01, 0.17] | 1.09 | [1.01, 1.19] | 0.249 |
| FPN-RTN | MA : RES | 0.03 | [-0.12, 0.17] | 1.03 | [0.89, 1.19] | 0.918 |
|  | VU : RES | 0.08 | [-0.05, 0.2] | 1.08 | [0.95, 1.23] | 0.637 |
|  | HC : RES | 0.03 | [-0.05, 0.11] | 1.03 | [0.95, 1.11] | 0.740 |
| FPN-SN | MA : RES | -0.14 | [-0.29, 0.01] | 0.87 | [0.75, 1.01] | 0.513 |
|  | VU : RES | -0.02 | [-0.15, 0.1] | 0.98 | [0.86, 1.11] | 0.879 |
|  | HC : RES | -0.08 | [-0.16, 0] | 0.93 | [0.85, 1] | 0.339 |
| FPN-SMN(H) | MA : RES | 0.11 | [-0.03, 0.26] | 1.12 | [0.97, 1.3] | 0.588 |
|  | VU : RES | 0.04 | [-0.08, 0.17] | 1.04 | [0.92, 1.18] | 0.820 |
|  | HC : RES | 0.03 | [-0.05, 0.11] | 1.03 | [0.95, 1.11] | 0.726 |
| FPN-SMN(M) | MA : RES | 0.03 | [-0.12, 0.17] | 1.03 | [0.89, 1.19] | 0.918 |
|  | VU : RES | 0.03 | [-0.09, 0.16] | 1.03 | [0.91, 1.17] | 0.858 |
|  | HC : RES | -0.09 | [-0.17, -0.01] | 0.91 | [0.84, 0.99] | 0.249 |
| FPN-VN | MA : RES | -0.04 | [-0.18, 0.11] | 0.96 | [0.83, 1.12] | 0.917 |
|  | VU : RES | 0.04 | [-0.09, 0.16] | 1.04 | [0.91, 1.18] | 0.820 |
|  | HC : RES | -0.02 | [-0.1, 0.06] | 0.98 | [0.9, 1.06] | 0.829 |
| FPN-VAN | MA : RES | -0.04 | [-0.18, 0.11] | 0.96 | [0.83, 1.12] | 0.917 |
|  | VU : RES | -0.13 | [-0.25, 0] | 0.88 | [0.78, 1] | 0.379 |
|  | HC : RES | -0.06 | [-0.13, 0.02] | 0.95 | [0.87, 1.02] | 0.484 |
| RTN-Amygdala | MA : RES | -0.04 | [-0.18, 0.11] | 0.96 | [0.83, 1.12] | 0.917 |
|  | VU : RES | -0.08 | [-0.2, 0.05] | 0.92 | [0.82, 1.05] | 0.630 |
|  | HC : RES | 0.02 | [-0.06, 0.09] | 1.02 | [0.94, 1.1] | 0.886 |
| RTN-Hippocampus | MA : RES | 0.03 | [-0.12, 0.17] | 1.03 | [0.89, 1.19] | 0.918 |
|  | VU : RES | 0.09 | [-0.03, 0.21] | 1.09 | [0.97, 1.24] | 0.562 |
|  | HC : RES | 0.00 | [-0.08, 0.08] | 1.00 | [0.92, 1.08] | 0.980 |
| RTN-RTN | MA : RES | -0.18 | [-0.33, -0.03] | 0.83 | [0.72, 0.97] | 0.513 |
|  | VU : RES | -0.03 | [-0.16, 0.1] | 0.97 | [0.86, 1.1] | 0.876 |
|  | HC : RES | 0.06 | [-0.02, 0.14] | 1.06 | [0.98, 1.15] | 0.433 |
| RTN-SN | MA : RES | 0.14 | [0, 0.29] | 1.16 | [1, 1.34] | 0.513 |
|  | VU : RES | 0.14 | [0.01, 0.27] | 1.15 | [1.01, 1.31] | 0.302 |
|  | HC : RES | -0.03 | [-0.11, 0.05] | 0.97 | [0.89, 1.05] | 0.726 |
| RTN-SMN(H) | MA : RES | -0.06 | [-0.2, 0.09] | 0.94 | [0.82, 1.09] | 0.917 |
|  | VU : RES | 0.03 | [-0.09, 0.15] | 1.03 | [0.91, 1.17] | 0.866 |
|  | HC : RES | -0.05 | [-0.13, 0.03] | 0.95 | [0.88, 1.03] | 0.494 |
| RTN-SMN(M) | MA : RES | -0.14 | [-0.29, 0.01] | 0.87 | [0.75, 1.01] | 0.513 |
|  | VU : RES | -0.07 | [-0.2, 0.06] | 0.93 | [0.82, 1.06] | 0.651 |
|  | HC : RES | -0.08 | [-0.16, 0] | 0.92 | [0.85, 1] | 0.304 |
| RTN-VN | MA : RES | -0.06 | [-0.22, 0.09] | 0.94 | [0.81, 1.1] | 0.917 |
|  | VU : RES | -0.02 | [-0.15, 0.11] | 0.98 | [0.86, 1.12] | 0.929 |
|  | HC : RES | 0.02 | [-0.06, 0.11] | 1.03 | [0.94, 1.11] | 0.767 |
| RTN-VAN | MA : RES | 0.12 | [-0.02, 0.27] | 1.13 | [0.98, 1.31] | 0.513 |
|  | VU : RES | 0.03 | [-0.09, 0.16] | 1.03 | [0.91, 1.17] | 0.858 |
|  | HC : RES | 0.00 | [-0.08, 0.08] | 1.00 | [0.92, 1.08] | 0.980 |
| SN-Amygdala | MA : RES | 0.00 | [-0.14, 0.15] | 1.00 | [0.87, 1.16] | 0.985 |
|  | VU : RES | -0.09 | [-0.21, 0.04] | 0.92 | [0.81, 1.04] | 0.567 |
|  | HC : RES | 0.02 | [-0.06, 0.1] | 1.02 | [0.95, 1.11] | 0.767 |
| SN-Hippocampus | MA : RES | 0.06 | [-0.09, 0.2] | 1.06 | [0.91, 1.23] | 0.917 |
|  | VU : RES | 0.16 | [0.03, 0.28] | 1.17 | [1.03, 1.33] | 0.265 |
|  | HC : RES | 0.02 | [-0.06, 0.09] | 1.02 | [0.94, 1.1] | 0.886 |
| SN-SN | MA : RES | -0.01 | [-0.16, 0.14] | 0.99 | [0.85, 1.15] | 0.973 |
|  | VU : RES | 0.11 | [-0.02, 0.24] | 1.12 | [0.98, 1.27] | 0.497 |
|  | HC : RES | 0.04 | [-0.04, 0.12] | 1.04 | [0.96, 1.13] | 0.664 |
| SN-SMN(H) | MA : RES | 0.05 | [-0.09, 0.2] | 1.06 | [0.91, 1.22] | 0.917 |
|  | VU : RES | 0.04 | [-0.08, 0.17] | 1.05 | [0.92, 1.18] | 0.820 |
|  | HC : RES | 0.09 | [0.01, 0.17] | 1.09 | [1.01, 1.18] | 0.249 |
| SN-SMN(M) | MA : RES | 0.09 | [-0.06, 0.23] | 1.09 | [0.94, 1.26] | 0.788 |
|  | VU : RES | -0.06 | [-0.18, 0.07] | 0.94 | [0.83, 1.07] | 0.742 |
|  | HC : RES | 0.00 | [-0.07, 0.08] | 1.00 | [0.93, 1.09] | 0.972 |
| SN-VN | MA : RES | 0.05 | [-0.09, 0.2] | 1.06 | [0.91, 1.22] | 0.917 |
|  | VU : RES | 0.00 | [-0.13, 0.13] | 1.00 | [0.88, 1.13] | 0.997 |
|  | HC : RES | 0.00 | [-0.08, 0.08] | 1.00 | [0.92, 1.08] | 0.972 |
| SN-VAN | MA : RES | 0.00 | [-0.15, 0.15] | 1.00 | [0.86, 1.16] | 0.988 |
|  | VU : RES | 0.01 | [-0.11, 0.14] | 1.01 | [0.89, 1.15] | 0.959 |
|  | HC : RES | 0.03 | [-0.05, 0.11] | 1.03 | [0.95, 1.12] | 0.726 |
| SMN(H)-Amygdala | MA : RES | 0.02 | [-0.13, 0.17] | 1.02 | [0.88, 1.18] | 0.918 |
|  | VU : RES | 0.10 | [-0.02, 0.23] | 1.11 | [0.98, 1.26] | 0.544 |
|  | HC : RES | 0.00 | [-0.08, 0.08] | 1.00 | [0.93, 1.09] | 0.972 |
| SMN(H)-Hippocampus | MA : RES | -0.04 | [-0.18, 0.11] | 0.96 | [0.83, 1.12] | 0.917 |
|  | VU : RES | 0.09 | [-0.03, 0.22] | 1.10 | [0.97, 1.24] | 0.562 |
|  | HC : RES | 0.02 | [-0.06, 0.1] | 1.02 | [0.94, 1.1] | 0.844 |
| SMN(H)-SMN(H) | MA : RES | -0.08 | [-0.23, 0.06] | 0.92 | [0.79, 1.07] | 0.849 |
|  | VU : RES | -0.08 | [-0.21, 0.05] | 0.93 | [0.81, 1.05] | 0.638 |
|  | HC : RES | 0.01 | [-0.07, 0.08] | 1.01 | [0.93, 1.09] | 0.972 |
| SMN(H)-SMN(M) | MA : RES | -0.04 | [-0.19, 0.11] | 0.96 | [0.83, 1.11] | 0.917 |
|  | VU : RES | 0.03 | [-0.1, 0.15] | 1.03 | [0.91, 1.17] | 0.870 |
|  | HC : RES | 0.01 | [-0.07, 0.09] | 1.01 | [0.93, 1.09] | 0.929 |
| SMN(H)-VN | MA : RES | 0.03 | [-0.12, 0.17] | 1.03 | [0.89, 1.19] | 0.918 |
|  | VU : RES | 0.07 | [-0.05, 0.2] | 1.07 | [0.95, 1.22] | 0.651 |
|  | HC : RES | -0.06 | [-0.14, 0.02] | 0.94 | [0.87, 1.02] | 0.468 |
| SMN(H)-VAN | MA : RES | 0.01 | [-0.14, 0.15] | 1.01 | [0.87, 1.16] | 0.973 |
|  | VU : RES | -0.04 | [-0.16, 0.09] | 0.96 | [0.85, 1.09] | 0.820 |
|  | HC : RES | -0.01 | [-0.09, 0.06] | 0.99 | [0.91, 1.07] | 0.901 |
| SMN(M)-Amygdala | MA : RES | 0.02 | [-0.13, 0.16] | 1.02 | [0.88, 1.18] | 0.931 |
|  | VU : RES | 0.01 | [-0.12, 0.13] | 1.01 | [0.89, 1.14] | 0.968 |
|  | HC : RES | 0.06 | [-0.02, 0.13] | 1.06 | [0.98, 1.14] | 0.484 |
| SMN(M)-Hippocampus | MA : RES | 0.00 | [-0.15, 0.15] | 1.00 | [0.86, 1.16] | 0.985 |
|  | VU : RES | 0.05 | [-0.08, 0.17] | 1.05 | [0.92, 1.19] | 0.820 |
|  | HC : RES | 0.09 | [0.01, 0.17] | 1.10 | [1.01, 1.19] | 0.249 |
| SMN(M)-SMN(M) | MA : RES | -0.05 | [-0.19, 0.1] | 0.95 | [0.82, 1.11] | 0.917 |
|  | VU : RES | 0.08 | [-0.05, 0.21] | 1.08 | [0.96, 1.23] | 0.630 |
|  | HC : RES | 0.03 | [-0.05, 0.11] | 1.03 | [0.95, 1.12] | 0.726 |
| SMN(M)-VN | MA : RES | -0.11 | [-0.25, 0.04] | 0.90 | [0.78, 1.04] | 0.588 |
|  | VU : RES | -0.09 | [-0.22, 0.03] | 0.91 | [0.8, 1.03] | 0.562 |
|  | HC : RES | -0.01 | [-0.09, 0.07] | 0.99 | [0.92, 1.07] | 0.929 |
| SMN(M)-VAN | MA : RES | 0.10 | [-0.05, 0.25] | 1.10 | [0.95, 1.28] | 0.625 |
|  | VU : RES | -0.05 | [-0.17, 0.08] | 0.96 | [0.84, 1.08] | 0.820 |
|  | HC : RES | 0.01 | [-0.07, 0.08] | 1.01 | [0.93, 1.09] | 0.972 |
| VN-Amygdala | MA : RES | 0.11 | [-0.04, 0.26] | 1.12 | [0.96, 1.3] | 0.588 |
|  | VU : RES | 0.12 | [-0.01, 0.24] | 1.12 | [0.99, 1.27] | 0.497 |
|  | HC : RES | 0.10 | [0.02, 0.18] | 1.10 | [1.02, 1.19] | 0.249 |
| VN-Hippocampus | MA : RES | 0.13 | [-0.02, 0.28] | 1.14 | [0.98, 1.33] | 0.513 |
|  | VU : RES | 0.15 | [0.02, 0.28] | 1.16 | [1.02, 1.32] | 0.302 |
|  | HC : RES | 0.07 | [-0.01, 0.15] | 1.07 | [0.99, 1.16] | 0.339 |
| VN-VN | MA : RES | -0.08 | [-0.24, 0.08] | 0.92 | [0.79, 1.08] | 0.917 |
|  | VU : RES | -0.20 | [-0.34, -0.07] | 0.82 | [0.71, 0.94] | 0.246 |
|  | HC : RES | 0.05 | [-0.04, 0.13] | 1.05 | [0.96, 1.14] | 0.596 |
| VN-VAN | MA : RES | -0.04 | [-0.19, 0.11] | 0.96 | [0.83, 1.12] | 0.917 |
|  | VU : RES | -0.02 | [-0.15, 0.11] | 0.98 | [0.86, 1.11] | 0.879 |
|  | HC : RES | -0.04 | [-0.12, 0.04] | 0.96 | [0.89, 1.05] | 0.700 |
| VAN-Amygdala | MA : RES | 0.02 | [-0.12, 0.17] | 1.02 | [0.88, 1.19] | 0.918 |
|  | VU : RES | -0.12 | [-0.24, 0.01] | 0.89 | [0.79, 1.01] | 0.497 |
|  | HC : RES | -0.09 | [-0.17, -0.01] | 0.91 | [0.84, 0.99] | 0.249 |
| VAN-Hippocampus | MA : RES | -0.03 | [-0.18, 0.12] | 0.97 | [0.84, 1.13] | 0.918 |
|  | VU : RES | 0.05 | [-0.08, 0.18] | 1.05 | [0.93, 1.2] | 0.820 |
|  | HC : RES | 0.09 | [0.01, 0.18] | 1.10 | [1.01, 1.19] | 0.249 |
| VAN-VAN | MA : RES | 0.01 | [-0.14, 0.16] | 1.01 | [0.87, 1.17] | 0.973 |
|  | VU : RES | 0.17 | [0.04, 0.29] | 1.18 | [1.04, 1.34] | 0.265 |
|  | HC : RES | 0.07 | [-0.01, 0.15] | 1.07 | [0.99, 1.16] | 0.339 |

*Note*. Abbreviations explained: auditory network (AN), Cingulo-parietal network (CPN), Cingulo-opecular network (CON), dorsal attention network (DAN), default mode network (DMN), frontal-parietal network (FPN), retrosplenial temporal network (RTN), salience network (SN), sensorimotor hand network (SMN[H]), sensorimotor mouth network (SMN[M]), visual network (VN), ventral attention network (VAN)

### Table S11. Externalizing group comparisons

| **Variables** | **Group comparisons** | **B** | **95% CI** | **Odds** | **95% CI** | ***p*FDR** |
| --- | --- | --- | --- | --- | --- | --- |
| AN-AN | MA : RES | -0.09 | [-0.31, 0.13] | 0.91 | [0.74, 1.13] | 0.777 |
|  | VU : RES | 0.01 | [-0.19, 0.22] | 1.01 | [0.83, 1.25] | 0.965 |
|  | HC : RES | 0.01 | [-0.06, 0.08] | 1.01 | [0.95, 1.08] | 0.900 |
| AN-Amygdala | MA : RES | -0.17 | [-0.38, 0.04] | 0.84 | [0.68, 1.04] | 0.538 |
|  | VU : RES | -0.04 | [-0.24, 0.16] | 0.96 | [0.78, 1.17] | 0.965 |
|  | HC : RES | 0.00 | [-0.06, 0.07] | 1.00 | [0.94, 1.07] | 0.971 |
| AN-CPN | MA : RES | 0.27 | [0.06, 0.49] | 1.31 | [1.06, 1.63] | 0.314 |
|  | VU : RES | 0.09 | [-0.12, 0.29] | 1.09 | [0.89, 1.34] | 0.965 |
|  | HC : RES | 0.02 | [-0.04, 0.09] | 1.02 | [0.96, 1.09] | 0.802 |
| AN-CON | MA : RES | 0.01 | [-0.21, 0.22] | 1.01 | [0.81, 1.25] | 0.983 |
|  | VU : RES | 0.07 | [-0.14, 0.27] | 1.07 | [0.87, 1.31] | 0.965 |
|  | HC : RES | 0.04 | [-0.03, 0.1] | 1.04 | [0.97, 1.11] | 0.697 |
| AN-DAN | MA : RES | -0.05 | [-0.26, 0.16] | 0.95 | [0.77, 1.17] | 0.840 |
|  | VU : RES | 0.04 | [-0.17, 0.24] | 1.04 | [0.85, 1.27] | 0.965 |
|  | HC : RES | -0.05 | [-0.11, 0.02] | 0.95 | [0.89, 1.02] | 0.558 |
| AN-DMN | MA : RES | -0.06 | [-0.27, 0.15] | 0.94 | [0.76, 1.16] | 0.839 |
|  | VU : RES | 0.08 | [-0.13, 0.28] | 1.08 | [0.88, 1.33] | 0.965 |
|  | HC : RES | 0.03 | [-0.03, 0.1] | 1.03 | [0.97, 1.1] | 0.736 |
| AN-FPN | MA : RES | -0.07 | [-0.28, 0.13] | 0.93 | [0.76, 1.14] | 0.839 |
|  | VU : RES | 0.00 | [-0.2, 0.2] | 1.00 | [0.82, 1.22] | 0.987 |
|  | HC : RES | -0.01 | [-0.07, 0.06] | 0.99 | [0.93, 1.06] | 0.927 |
| AN-hippocampus | MA : RES | -0.12 | [-0.34, 0.09] | 0.88 | [0.71, 1.09] | 0.748 |
|  | VU : RES | -0.16 | [-0.37, 0.05] | 0.85 | [0.69, 1.05] | 0.965 |
|  | HC : RES | -0.06 | [-0.12, 0.01] | 0.94 | [0.88, 1.01] | 0.456 |
| AN-RTN | MA : RES | 0.25 | [0.03, 0.46] | 1.28 | [1.03, 1.59] | 0.314 |
|  | VU : RES | 0.16 | [-0.05, 0.36] | 1.17 | [0.95, 1.44] | 0.965 |
|  | HC : RES | 0.01 | [-0.05, 0.08] | 1.01 | [0.95, 1.08] | 0.851 |
| AN-SN | MA : RES | 0.18 | [-0.03, 0.39] | 1.19 | [0.97, 1.47] | 0.538 |
|  | VU : RES | -0.01 | [-0.22, 0.19] | 0.99 | [0.81, 1.21] | 0.965 |
|  | HC : RES | 0.03 | [-0.03, 0.1] | 1.03 | [0.97, 1.1] | 0.736 |
| AN-SMN(H) | MA : RES | -0.25 | [-0.48, -0.03] | 0.78 | [0.62, 0.97] | 0.316 |
|  | VU : RES | -0.16 | [-0.38, 0.05] | 0.85 | [0.69, 1.05] | 0.965 |
|  | HC : RES | 0.00 | [-0.07, 0.06] | 1.00 | [0.93, 1.06] | 0.932 |
| AN-SMN(M) | MA : RES | -0.18 | [-0.4, 0.03] | 0.83 | [0.67, 1.03] | 0.538 |
|  | VU : RES | -0.02 | [-0.23, 0.18] | 0.98 | [0.8, 1.2] | 0.965 |
|  | HC : RES | -0.01 | [-0.08, 0.05] | 0.99 | [0.93, 1.06] | 0.900 |
| AN-VN | MA : RES | 0.09 | [-0.12, 0.31] | 1.10 | [0.89, 1.36] | 0.777 |
|  | VU : RES | -0.02 | [-0.23, 0.19] | 0.98 | [0.8, 1.21] | 0.965 |
|  | HC : RES | -0.02 | [-0.08, 0.05] | 0.98 | [0.92, 1.05] | 0.843 |
| AN-VAN | MA : RES | -0.08 | [-0.3, 0.14] | 0.93 | [0.74, 1.15] | 0.839 |
|  | VU : RES | -0.03 | [-0.24, 0.18] | 0.97 | [0.79, 1.2] | 0.965 |
|  | HC : RES | 0.00 | [-0.07, 0.06] | 1.00 | [0.93, 1.06] | 0.932 |
| CPN-Amygdala | MA : RES | -0.18 | [-0.39, 0.03] | 0.84 | [0.68, 1.03] | 0.538 |
|  | VU : RES | -0.08 | [-0.28, 0.13] | 0.93 | [0.76, 1.13] | 0.965 |
|  | HC : RES | -0.06 | [-0.13, 0] | 0.94 | [0.88, 1] | 0.406 |
| CPN-CPN | MA : RES | -0.02 | [-0.23, 0.19] | 0.98 | [0.79, 1.21] | 0.925 |
|  | VU : RES | 0.05 | [-0.15, 0.25] | 1.05 | [0.86, 1.29] | 0.965 |
|  | HC : RES | -0.02 | [-0.08, 0.05] | 0.98 | [0.92, 1.05] | 0.843 |
| CPN-CON | MA : RES | 0.21 | [0, 0.43] | 1.24 | [1, 1.53] | 0.444 |
|  | VU : RES | 0.05 | [-0.15, 0.26] | 1.05 | [0.86, 1.29] | 0.965 |
|  | HC : RES | 0.03 | [-0.03, 0.1] | 1.03 | [0.97, 1.1] | 0.736 |
| CPN-DAN | MA : RES | 0.18 | [-0.04, 0.39] | 1.19 | [0.96, 1.48] | 0.538 |
|  | VU : RES | -0.06 | [-0.26, 0.14] | 0.94 | [0.77, 1.15] | 0.965 |
|  | HC : RES | 0.03 | [-0.03, 0.1] | 1.03 | [0.97, 1.1] | 0.736 |
| CPN-DMN | MA : RES | -0.35 | [-0.57, -0.13] | 0.71 | [0.57, 0.87] | 0.153 |
|  | VU : RES | 0.02 | [-0.18, 0.22] | 1.02 | [0.83, 1.25] | 0.965 |
|  | HC : RES | -0.03 | [-0.09, 0.03] | 0.97 | [0.91, 1.04] | 0.778 |
| CPN-FPN | MA : RES | -0.07 | [-0.28, 0.14] | 0.93 | [0.75, 1.15] | 0.839 |
|  | VU : RES | 0.06 | [-0.14, 0.26] | 1.06 | [0.87, 1.3] | 0.965 |
|  | HC : RES | -0.02 | [-0.09, 0.04] | 0.98 | [0.92, 1.04] | 0.806 |
| CPN-Hippocampus | MA : RES | -0.02 | [-0.24, 0.2] | 0.98 | [0.79, 1.22] | 0.925 |
|  | VU : RES | -0.03 | [-0.24, 0.18] | 0.97 | [0.79, 1.2] | 0.965 |
|  | HC : RES | 0.02 | [-0.05, 0.09] | 1.02 | [0.95, 1.09] | 0.836 |
| CPN-RTN | MA : RES | -0.05 | [-0.26, 0.17] | 0.95 | [0.77, 1.18] | 0.840 |
|  | VU : RES | -0.09 | [-0.29, 0.12] | 0.92 | [0.75, 1.13] | 0.965 |
|  | HC : RES | 0.05 | [-0.01, 0.12] | 1.05 | [0.99, 1.13] | 0.456 |
| CPN-SN | MA : RES | 0.00 | [-0.21, 0.21] | 1.00 | [0.81, 1.24] | 0.987 |
|  | VU : RES | 0.04 | [-0.16, 0.24] | 1.04 | [0.85, 1.28] | 0.965 |
|  | HC : RES | 0.00 | [-0.07, 0.06] | 1.00 | [0.94, 1.06] | 0.971 |
| CPN-SMN(H) | MA : RES | 0.16 | [-0.05, 0.37] | 1.17 | [0.95, 1.45] | 0.538 |
|  | VU : RES | 0.18 | [-0.02, 0.39] | 1.20 | [0.98, 1.47] | 0.965 |
|  | HC : RES | 0.05 | [-0.01, 0.12] | 1.06 | [0.99, 1.13] | 0.456 |
| CPN-SMN(M) | MA : RES | 0.27 | [0.05, 0.48] | 1.30 | [1.06, 1.61] | 0.314 |
|  | VU : RES | 0.20 | [0, 0.4] | 1.22 | [1, 1.5] | 0.857 |
|  | HC : RES | 0.03 | [-0.03, 0.1] | 1.03 | [0.97, 1.1] | 0.736 |
| CPN-VN | MA : RES | 0.25 | [0.04, 0.46] | 1.28 | [1.04, 1.58] | 0.314 |
|  | VU : RES | 0.01 | [-0.2, 0.21] | 1.01 | [0.82, 1.24] | 0.965 |
|  | HC : RES | 0.04 | [-0.02, 0.11] | 1.04 | [0.98, 1.11] | 0.636 |
| CPN-VAN | MA : RES | -0.18 | [-0.39, 0.03] | 0.84 | [0.68, 1.03] | 0.538 |
|  | VU : RES | 0.02 | [-0.18, 0.22] | 1.02 | [0.84, 1.25] | 0.965 |
|  | HC : RES | -0.01 | [-0.07, 0.06] | 0.99 | [0.93, 1.06] | 0.932 |
| CON-Amygdala | MA : RES | 0.04 | [-0.16, 0.25] | 1.05 | [0.85, 1.29] | 0.840 |
|  | VU : RES | -0.06 | [-0.26, 0.14] | 0.94 | [0.77, 1.15] | 0.965 |
|  | HC : RES | 0.06 | [-0.01, 0.12] | 1.06 | [0.99, 1.13] | 0.456 |
| CON-CON | MA : RES | -0.13 | [-0.35, 0.1] | 0.88 | [0.7, 1.1] | 0.758 |
|  | VU : RES | -0.09 | [-0.3, 0.13] | 0.92 | [0.74, 1.13] | 0.965 |
|  | HC : RES | 0.05 | [-0.01, 0.12] | 1.06 | [0.99, 1.13] | 0.472 |
| CON-DAN | MA : RES | 0.00 | [-0.21, 0.21] | 1.00 | [0.81, 1.24] | 0.987 |
|  | VU : RES | 0.02 | [-0.18, 0.22] | 1.02 | [0.84, 1.25] | 0.965 |
|  | HC : RES | 0.01 | [-0.05, 0.07] | 1.01 | [0.95, 1.08] | 0.900 |
| CON-DMN | MA : RES | 0.05 | [-0.17, 0.27] | 1.05 | [0.85, 1.31] | 0.840 |
|  | VU : RES | 0.02 | [-0.18, 0.23] | 1.02 | [0.83, 1.26] | 0.965 |
|  | HC : RES | -0.04 | [-0.1, 0.03] | 0.96 | [0.9, 1.03] | 0.717 |
| CON-FPN | MA : RES | -0.06 | [-0.28, 0.15] | 0.94 | [0.76, 1.16] | 0.839 |
|  | VU : RES | -0.04 | [-0.24, 0.16] | 0.96 | [0.79, 1.18] | 0.965 |
|  | HC : RES | -0.04 | [-0.1, 0.03] | 0.96 | [0.9, 1.03] | 0.692 |
| CON-Hippocampus | MA : RES | -0.10 | [-0.3, 0.11] | 0.91 | [0.74, 1.11] | 0.772 |
|  | VU : RES | 0.04 | [-0.16, 0.25] | 1.05 | [0.85, 1.29] | 0.965 |
|  | HC : RES | 0.09 | [0.03, 0.16] | 1.10 | [1.03, 1.18] | 0.095 |
| CON-RTN | MA : RES | 0.25 | [0.04, 0.47] | 1.29 | [1.04, 1.6] | 0.314 |
|  | VU : RES | 0.08 | [-0.13, 0.28] | 1.08 | [0.88, 1.33] | 0.965 |
|  | HC : RES | 0.00 | [-0.07, 0.06] | 1.00 | [0.94, 1.07] | 0.985 |
| CON-SN | MA : RES | 0.15 | [-0.05, 0.36] | 1.17 | [0.95, 1.43] | 0.538 |
|  | VU : RES | -0.07 | [-0.28, 0.13] | 0.93 | [0.76, 1.14] | 0.965 |
|  | HC : RES | 0.03 | [-0.04, 0.09] | 1.03 | [0.96, 1.09] | 0.802 |
| CON-SMN(H) | MA : RES | -0.16 | [-0.38, 0.05] | 0.85 | [0.69, 1.05] | 0.538 |
|  | VU : RES | 0.08 | [-0.12, 0.28] | 1.08 | [0.88, 1.32] | 0.965 |
|  | HC : RES | 0.05 | [-0.02, 0.11] | 1.05 | [0.98, 1.12] | 0.576 |
| CON-SMN(M) | MA : RES | -0.02 | [-0.24, 0.2] | 0.98 | [0.79, 1.22] | 0.930 |
|  | VU : RES | 0.09 | [-0.12, 0.29] | 1.09 | [0.89, 1.34] | 0.965 |
|  | HC : RES | 0.03 | [-0.03, 0.1] | 1.04 | [0.97, 1.1] | 0.736 |
| CON-VN | MA : RES | 0.00 | [-0.21, 0.21] | 1.00 | [0.81, 1.24] | 0.987 |
|  | VU : RES | 0.01 | [-0.19, 0.21] | 1.01 | [0.83, 1.24] | 0.965 |
|  | HC : RES | 0.05 | [-0.02, 0.11] | 1.05 | [0.98, 1.12] | 0.550 |
| CON-VAN | MA : RES | -0.05 | [-0.27, 0.17] | 0.95 | [0.76, 1.18] | 0.840 |
|  | VU : RES | 0.05 | [-0.16, 0.26] | 1.05 | [0.85, 1.29] | 0.965 |
|  | HC : RES | -0.05 | [-0.11, 0.02] | 0.95 | [0.89, 1.02] | 0.558 |
| DAN-Amygdala | MA : RES | 0.05 | [-0.16, 0.27] | 1.06 | [0.85, 1.31] | 0.840 |
|  | VU : RES | -0.01 | [-0.21, 0.19] | 0.99 | [0.81, 1.21] | 0.965 |
|  | HC : RES | -0.04 | [-0.1, 0.03] | 0.96 | [0.9, 1.03] | 0.692 |
| DAN-DAN | MA : RES | -0.07 | [-0.29, 0.14] | 0.93 | [0.75, 1.15] | 0.839 |
|  | VU : RES | 0.01 | [-0.2, 0.21] | 1.01 | [0.82, 1.23] | 0.965 |
|  | HC : RES | 0.08 | [0.01, 0.14] | 1.08 | [1.01, 1.15] | 0.246 |
| DAN-DMN | MA : RES | 0.10 | [-0.12, 0.32] | 1.10 | [0.89, 1.37] | 0.772 |
|  | VU : RES | -0.01 | [-0.22, 0.2] | 0.99 | [0.8, 1.22] | 0.965 |
|  | HC : RES | -0.10 | [-0.17, -0.04] | 0.90 | [0.84, 0.97] | 0.095 |
| DAN-FPN | MA : RES | -0.14 | [-0.36, 0.08] | 0.87 | [0.7, 1.08] | 0.661 |
|  | VU : RES | 0.15 | [-0.05, 0.36] | 1.16 | [0.95, 1.43] | 0.965 |
|  | HC : RES | 0.02 | [-0.05, 0.08] | 1.02 | [0.95, 1.09] | 0.843 |
| DAN-Hippocampus | MA : RES | -0.02 | [-0.24, 0.19] | 0.98 | [0.79, 1.21] | 0.912 |
|  | VU : RES | 0.05 | [-0.16, 0.25] | 1.05 | [0.85, 1.29] | 0.965 |
|  | HC : RES | -0.03 | [-0.09, 0.04] | 0.97 | [0.91, 1.04] | 0.778 |
| DAN-RTN | MA : RES | -0.03 | [-0.25, 0.19] | 0.97 | [0.78, 1.21] | 0.908 |
|  | VU : RES | -0.07 | [-0.28, 0.14] | 0.93 | [0.76, 1.14] | 0.965 |
|  | HC : RES | 0.04 | [-0.02, 0.11] | 1.04 | [0.98, 1.12] | 0.601 |
| DAN-SN | MA : RES | 0.06 | [-0.15, 0.28] | 1.07 | [0.86, 1.32] | 0.839 |
|  | VU : RES | 0.07 | [-0.14, 0.27] | 1.07 | [0.87, 1.31] | 0.965 |
|  | HC : RES | -0.05 | [-0.11, 0.02] | 0.95 | [0.89, 1.02] | 0.550 |
| DAN-SMN(H) | MA : RES | -0.15 | [-0.36, 0.06] | 0.86 | [0.7, 1.06] | 0.562 |
|  | VU : RES | 0.01 | [-0.19, 0.22] | 1.01 | [0.83, 1.24] | 0.965 |
|  | HC : RES | -0.02 | [-0.09, 0.04] | 0.98 | [0.92, 1.04] | 0.803 |
| DAN-SMN(M) | MA : RES | 0.12 | [-0.09, 0.34] | 1.13 | [0.91, 1.4] | 0.748 |
|  | VU : RES | 0.22 | [0.01, 0.42] | 1.25 | [1.01, 1.53] | 0.857 |
|  | HC : RES | 0.01 | [-0.05, 0.08] | 1.01 | [0.95, 1.08] | 0.851 |
| DAN-VN | MA : RES | 0.08 | [-0.13, 0.29] | 1.08 | [0.87, 1.34] | 0.839 |
|  | VU : RES | -0.08 | [-0.28, 0.13] | 0.92 | [0.75, 1.13] | 0.965 |
|  | HC : RES | 0.07 | [0.01, 0.14] | 1.08 | [1.01, 1.15] | 0.261 |
| DAN-VAN | MA : RES | -0.01 | [-0.23, 0.21] | 0.99 | [0.79, 1.24] | 0.981 |
|  | VU : RES | -0.03 | [-0.24, 0.18] | 0.97 | [0.79, 1.2] | 0.965 |
|  | HC : RES | -0.13 | [-0.19, -0.06] | 0.88 | [0.83, 0.94] | 0.019 |
| DMN-Amygdala | MA : RES | -0.05 | [-0.25, 0.16] | 0.95 | [0.78, 1.17] | 0.840 |
|  | VU : RES | 0.13 | [-0.07, 0.34] | 1.14 | [0.93, 1.4] | 0.965 |
|  | HC : RES | 0.02 | [-0.04, 0.09] | 1.02 | [0.96, 1.09] | 0.809 |
| DMN-DMN | MA : RES | -0.23 | [-0.46, 0] | 0.79 | [0.63, 1] | 0.430 |
|  | VU : RES | -0.02 | [-0.23, 0.19] | 0.98 | [0.79, 1.21] | 0.965 |
|  | HC : RES | 0.01 | [-0.05, 0.08] | 1.01 | [0.95, 1.09] | 0.851 |
| DMN-FPN | MA : RES | 0.10 | [-0.11, 0.31] | 1.10 | [0.9, 1.36] | 0.772 |
|  | VU : RES | -0.12 | [-0.32, 0.08] | 0.89 | [0.73, 1.09] | 0.965 |
|  | HC : RES | -0.02 | [-0.08, 0.05] | 0.98 | [0.92, 1.05] | 0.843 |
| DMN-Hippocampus | MA : RES | 0.08 | [-0.13, 0.3] | 1.09 | [0.87, 1.35] | 0.839 |
|  | VU : RES | 0.12 | [-0.09, 0.33] | 1.13 | [0.92, 1.39] | 0.965 |
|  | HC : RES | 0.07 | [0.01, 0.14] | 1.08 | [1.01, 1.15] | 0.269 |
| DMN-RTN | MA : RES | -0.06 | [-0.28, 0.16] | 0.94 | [0.76, 1.17] | 0.839 |
|  | VU : RES | 0.10 | [-0.1, 0.31] | 1.11 | [0.9, 1.36] | 0.965 |
|  | HC : RES | -0.03 | [-0.1, 0.03] | 0.97 | [0.91, 1.03] | 0.736 |
| DMN-SN | MA : RES | -0.17 | [-0.39, 0.04] | 0.84 | [0.68, 1.04] | 0.538 |
|  | VU : RES | -0.01 | [-0.21, 0.19] | 0.99 | [0.81, 1.21] | 0.965 |
|  | HC : RES | 0.00 | [-0.07, 0.06] | 1.00 | [0.93, 1.06] | 0.960 |
| DMN-SMN(H) | MA : RES | 0.08 | [-0.14, 0.29] | 1.08 | [0.87, 1.34] | 0.839 |
|  | VU : RES | -0.03 | [-0.23, 0.18] | 0.97 | [0.79, 1.2] | 0.965 |
|  | HC : RES | -0.01 | [-0.08, 0.05] | 0.99 | [0.92, 1.05] | 0.869 |
| DMN-SMN(M) | MA : RES | -0.04 | [-0.25, 0.17] | 0.96 | [0.78, 1.19] | 0.840 |
|  | VU : RES | -0.08 | [-0.29, 0.12] | 0.92 | [0.75, 1.13] | 0.965 |
|  | HC : RES | -0.03 | [-0.09, 0.04] | 0.97 | [0.91, 1.04] | 0.778 |
| DMN-VN | MA : RES | 0.16 | [-0.06, 0.39] | 1.18 | [0.94, 1.47] | 0.552 |
|  | VU : RES | 0.04 | [-0.17, 0.25] | 1.04 | [0.84, 1.28] | 0.965 |
|  | HC : RES | -0.01 | [-0.07, 0.06] | 1.00 | [0.93, 1.06] | 0.932 |
| DMN-VAN | MA : RES | -0.06 | [-0.28, 0.15] | 0.94 | [0.75, 1.17] | 0.839 |
|  | VU : RES | 0.06 | [-0.14, 0.27] | 1.07 | [0.87, 1.31] | 0.965 |
|  | HC : RES | 0.06 | [-0.01, 0.12] | 1.06 | [0.99, 1.13] | 0.456 |
| FPN-Amygdala | MA : RES | 0.24 | [0.02, 0.46] | 1.27 | [1.02, 1.59] | 0.316 |
|  | VU : RES | 0.09 | [-0.12, 0.3] | 1.09 | [0.89, 1.35] | 0.965 |
|  | HC : RES | 0.07 | [0, 0.14] | 1.07 | [1, 1.14] | 0.316 |
| FPN-FPN | MA : RES | 0.11 | [-0.1, 0.31] | 1.11 | [0.9, 1.37] | 0.772 |
|  | VU : RES | -0.08 | [-0.29, 0.12] | 0.92 | [0.75, 1.13] | 0.965 |
|  | HC : RES | -0.02 | [-0.08, 0.05] | 0.98 | [0.92, 1.05] | 0.843 |
| FPN-Hippocampus | MA : RES | 0.10 | [-0.12, 0.31] | 1.10 | [0.89, 1.37] | 0.772 |
|  | VU : RES | 0.05 | [-0.16, 0.26] | 1.05 | [0.86, 1.3] | 0.965 |
|  | HC : RES | 0.06 | [-0.01, 0.12] | 1.06 | [0.99, 1.13] | 0.456 |
| FPN-RTN | MA : RES | 0.01 | [-0.2, 0.22] | 1.01 | [0.82, 1.25] | 0.974 |
|  | VU : RES | 0.13 | [-0.07, 0.34] | 1.14 | [0.93, 1.4] | 0.965 |
|  | HC : RES | 0.02 | [-0.05, 0.08] | 1.02 | [0.95, 1.08] | 0.846 |
| FPN-SN | MA : RES | 0.04 | [-0.17, 0.25] | 1.04 | [0.84, 1.29] | 0.840 |
|  | VU : RES | -0.03 | [-0.24, 0.17] | 0.97 | [0.79, 1.19] | 0.965 |
|  | HC : RES | -0.02 | [-0.09, 0.04] | 0.98 | [0.91, 1.04] | 0.802 |
| FPN-SMN(H) | MA : RES | -0.09 | [-0.29, 0.11] | 0.91 | [0.75, 1.11] | 0.772 |
|  | VU : RES | 0.02 | [-0.18, 0.21] | 1.02 | [0.83, 1.24] | 0.965 |
|  | HC : RES | 0.03 | [-0.04, 0.09] | 1.03 | [0.96, 1.1] | 0.778 |
| FPN-SMN(M) | MA : RES | 0.06 | [-0.15, 0.27] | 1.06 | [0.86, 1.31] | 0.839 |
|  | VU : RES | -0.03 | [-0.23, 0.17] | 0.97 | [0.79, 1.18] | 0.965 |
|  | HC : RES | -0.06 | [-0.13, 0] | 0.94 | [0.88, 1] | 0.406 |
| FPN-VN | MA : RES | 0.04 | [-0.17, 0.26] | 1.05 | [0.84, 1.29] | 0.840 |
|  | VU : RES | 0.16 | [-0.05, 0.37] | 1.17 | [0.95, 1.44] | 0.965 |
|  | HC : RES | 0.02 | [-0.05, 0.08] | 1.02 | [0.95, 1.08] | 0.843 |
| FPN-VAN | MA : RES | -0.04 | [-0.25, 0.17] | 0.96 | [0.78, 1.18] | 0.840 |
|  | VU : RES | -0.10 | [-0.3, 0.1] | 0.90 | [0.74, 1.11] | 0.965 |
|  | HC : RES | -0.07 | [-0.13, -0.01] | 0.93 | [0.87, 0.99] | 0.275 |
| RTN-Amygdala | MA : RES | -0.16 | [-0.37, 0.05] | 0.85 | [0.69, 1.05] | 0.538 |
|  | VU : RES | -0.01 | [-0.21, 0.2] | 0.99 | [0.81, 1.22] | 0.965 |
|  | HC : RES | -0.01 | [-0.07, 0.06] | 0.99 | [0.93, 1.06] | 0.927 |
| RTN-Hippocampus | MA : RES | 0.24 | [0.03, 0.45] | 1.28 | [1.03, 1.57] | 0.314 |
|  | VU : RES | 0.20 | [-0.01, 0.4] | 1.22 | [0.99, 1.49] | 0.857 |
|  | HC : RES | 0.01 | [-0.05, 0.08] | 1.01 | [0.95, 1.08] | 0.846 |
| RTN-RTN | MA : RES | -0.29 | [-0.5, -0.08] | 0.75 | [0.6, 0.93] | 0.314 |
|  | VU : RES | -0.07 | [-0.28, 0.13] | 0.93 | [0.76, 1.14] | 0.965 |
|  | HC : RES | 0.08 | [0.01, 0.14] | 1.08 | [1.01, 1.15] | 0.261 |
| RTN-SN | MA : RES | 0.14 | [-0.07, 0.36] | 1.16 | [0.93, 1.43] | 0.605 |
|  | VU : RES | 0.24 | [0.03, 0.44] | 1.27 | [1.03, 1.55] | 0.857 |
|  | HC : RES | -0.03 | [-0.09, 0.04] | 0.97 | [0.91, 1.04] | 0.778 |
| RTN-SMN(H) | MA : RES | 0.05 | [-0.16, 0.26] | 1.05 | [0.85, 1.3] | 0.840 |
|  | VU : RES | 0.04 | [-0.16, 0.25] | 1.05 | [0.85, 1.28] | 0.965 |
|  | HC : RES | -0.02 | [-0.09, 0.04] | 0.98 | [0.92, 1.04] | 0.806 |
| RTN-SMN(M) | MA : RES | 0.12 | [-0.09, 0.33] | 1.13 | [0.92, 1.4] | 0.748 |
|  | VU : RES | -0.01 | [-0.21, 0.19] | 0.99 | [0.81, 1.21] | 0.965 |
|  | HC : RES | 0.01 | [-0.06, 0.07] | 1.01 | [0.94, 1.07] | 0.932 |
| RTN-VN | MA : RES | 0.06 | [-0.16, 0.28] | 1.06 | [0.85, 1.32] | 0.839 |
|  | VU : RES | -0.19 | [-0.4, 0.03] | 0.83 | [0.67, 1.03] | 0.965 |
|  | HC : RES | 0.03 | [-0.04, 0.09] | 1.03 | [0.96, 1.1] | 0.778 |
| RTN-VAN | MA : RES | 0.10 | [-0.12, 0.32] | 1.11 | [0.89, 1.38] | 0.772 |
|  | VU : RES | 0.12 | [-0.09, 0.33] | 1.13 | [0.92, 1.4] | 0.965 |
|  | HC : RES | -0.01 | [-0.07, 0.06] | 0.99 | [0.93, 1.06] | 0.927 |
| SN-Amygdala | MA : RES | -0.19 | [-0.41, 0.02] | 0.82 | [0.67, 1.02] | 0.538 |
|  | VU : RES | -0.28 | [-0.48, -0.08] | 0.76 | [0.62, 0.93] | 0.717 |
|  | HC : RES | 0.02 | [-0.05, 0.08] | 1.02 | [0.95, 1.08] | 0.846 |
| SN-Hippocampus | MA : RES | 0.04 | [-0.17, 0.25] | 1.04 | [0.84, 1.29] | 0.840 |
|  | VU : RES | -0.02 | [-0.22, 0.19] | 0.98 | [0.8, 1.2] | 0.965 |
|  | HC : RES | 0.02 | [-0.04, 0.09] | 1.02 | [0.96, 1.09] | 0.802 |
| SN-SN | MA : RES | -0.04 | [-0.25, 0.17] | 0.96 | [0.78, 1.19] | 0.840 |
|  | VU : RES | 0.03 | [-0.18, 0.23] | 1.03 | [0.84, 1.26] | 0.965 |
|  | HC : RES | 0.02 | [-0.05, 0.08] | 1.02 | [0.95, 1.08] | 0.843 |
| SN-SMN(H) | MA : RES | 0.03 | [-0.17, 0.24] | 1.03 | [0.84, 1.27] | 0.870 |
|  | VU : RES | 0.03 | [-0.17, 0.23] | 1.03 | [0.84, 1.26] | 0.965 |
|  | HC : RES | 0.06 | [0, 0.13] | 1.07 | [1, 1.14] | 0.363 |
| SN-SMN(M) | MA : RES | 0.17 | [-0.04, 0.38] | 1.18 | [0.96, 1.46] | 0.538 |
|  | VU : RES | -0.11 | [-0.31, 0.09] | 0.89 | [0.73, 1.09] | 0.965 |
|  | HC : RES | -0.03 | [-0.1, 0.03] | 0.97 | [0.91, 1.03] | 0.755 |
| SN-VN | MA : RES | 0.12 | [-0.1, 0.33] | 1.12 | [0.91, 1.39] | 0.772 |
|  | VU : RES | 0.07 | [-0.13, 0.28] | 1.07 | [0.88, 1.32] | 0.965 |
|  | HC : RES | 0.02 | [-0.05, 0.08] | 1.02 | [0.95, 1.08] | 0.843 |
| SN-VAN | MA : RES | -0.06 | [-0.27, 0.15] | 0.94 | [0.76, 1.16] | 0.839 |
|  | VU : RES | -0.02 | [-0.23, 0.18] | 0.98 | [0.8, 1.2] | 0.965 |
|  | HC : RES | 0.01 | [-0.06, 0.07] | 1.01 | [0.94, 1.07] | 0.927 |
| SMN(H)-Amygdala | MA : RES | -0.07 | [-0.28, 0.15] | 0.93 | [0.75, 1.16] | 0.839 |
|  | VU : RES | -0.06 | [-0.27, 0.14] | 0.94 | [0.76, 1.16] | 0.965 |
|  | HC : RES | 0.04 | [-0.03, 0.11] | 1.04 | [0.97, 1.11] | 0.692 |
| SMN(H)-Hippocampus | MA : RES | -0.16 | [-0.37, 0.06] | 0.86 | [0.69, 1.06] | 0.538 |
|  | VU : RES | -0.01 | [-0.22, 0.19] | 0.99 | [0.8, 1.21] | 0.965 |
|  | HC : RES | -0.03 | [-0.1, 0.03] | 0.97 | [0.91, 1.03] | 0.736 |
| SMN(H)-SMN(H) | MA : RES | -0.11 | [-0.32, 0.11] | 0.90 | [0.73, 1.11] | 0.772 |
|  | VU : RES | -0.14 | [-0.35, 0.07] | 0.87 | [0.7, 1.07] | 0.965 |
|  | HC : RES | -0.03 | [-0.09, 0.04] | 0.97 | [0.91, 1.04] | 0.778 |
| SMN(H)-SMN(M) | MA : RES | -0.17 | [-0.39, 0.05] | 0.84 | [0.68, 1.05] | 0.538 |
|  | VU : RES | -0.04 | [-0.25, 0.16] | 0.96 | [0.78, 1.18] | 0.965 |
|  | HC : RES | -0.02 | [-0.08, 0.05] | 0.98 | [0.92, 1.05] | 0.836 |
| SMN(H)-VN | MA : RES | 0.06 | [-0.15, 0.27] | 1.06 | [0.86, 1.31] | 0.839 |
|  | VU : RES | -0.04 | [-0.25, 0.16] | 0.96 | [0.78, 1.18] | 0.965 |
|  | HC : RES | -0.03 | [-0.09, 0.04] | 0.97 | [0.91, 1.04] | 0.802 |
| SMN(H)-VAN | MA : RES | -0.09 | [-0.3, 0.12] | 0.92 | [0.74, 1.13] | 0.777 |
|  | VU : RES | -0.20 | [-0.41, 0] | 0.82 | [0.67, 1] | 0.857 |
|  | HC : RES | -0.02 | [-0.09, 0.04] | 0.98 | [0.92, 1.04] | 0.802 |
| SMN(M)-Amygdala | MA : RES | -0.03 | [-0.23, 0.18] | 0.97 | [0.79, 1.2] | 0.908 |
|  | VU : RES | -0.07 | [-0.27, 0.13] | 0.93 | [0.76, 1.14] | 0.965 |
|  | HC : RES | 0.05 | [-0.01, 0.12] | 1.05 | [0.99, 1.12] | 0.456 |
| SMN(M)-Hippocampus | MA : RES | -0.11 | [-0.32, 0.1] | 0.90 | [0.73, 1.11] | 0.772 |
|  | VU : RES | 0.06 | [-0.14, 0.27] | 1.06 | [0.87, 1.31] | 0.965 |
|  | HC : RES | 0.12 | [0.05, 0.19] | 1.13 | [1.06, 1.2] | 0.019 |
| SMN(M)-SMN(M) | MA : RES | -0.09 | [-0.31, 0.12] | 0.91 | [0.74, 1.13] | 0.777 |
|  | VU : RES | -0.14 | [-0.34, 0.07] | 0.87 | [0.71, 1.07] | 0.965 |
|  | HC : RES | -0.02 | [-0.09, 0.04] | 0.98 | [0.91, 1.04] | 0.802 |
| SMN(M)-VN | MA : RES | -0.06 | [-0.27, 0.15] | 0.94 | [0.76, 1.16] | 0.839 |
|  | VU : RES | -0.04 | [-0.24, 0.16] | 0.96 | [0.79, 1.18] | 0.965 |
|  | HC : RES | 0.01 | [-0.05, 0.08] | 1.01 | [0.95, 1.08] | 0.876 |
| SMN(M)-VAN | MA : RES | -0.11 | [-0.33, 0.1] | 0.89 | [0.72, 1.1] | 0.772 |
|  | VU : RES | -0.15 | [-0.36, 0.05] | 0.86 | [0.7, 1.05] | 0.965 |
|  | HC : RES | -0.05 | [-0.12, 0.01] | 0.95 | [0.89, 1.01] | 0.456 |
| VN-Amygdala | MA : RES | 0.03 | [-0.19, 0.24] | 1.03 | [0.83, 1.27] | 0.908 |
|  | VU : RES | -0.12 | [-0.33, 0.08] | 0.88 | [0.72, 1.09] | 0.965 |
|  | HC : RES | 0.02 | [-0.05, 0.08] | 1.02 | [0.96, 1.09] | 0.836 |
| VN-Hippocampus | MA : RES | 0.11 | [-0.1, 0.32] | 1.11 | [0.9, 1.37] | 0.772 |
|  | VU : RES | 0.05 | [-0.15, 0.26] | 1.06 | [0.86, 1.3] | 0.965 |
|  | HC : RES | 0.08 | [0.01, 0.14] | 1.08 | [1.01, 1.15] | 0.258 |
| VN-VN | MA : RES | -0.04 | [-0.27, 0.18] | 0.96 | [0.77, 1.2] | 0.842 |
|  | VU : RES | 0.00 | [-0.21, 0.22] | 1.00 | [0.81, 1.24] | 0.987 |
|  | HC : RES | -0.01 | [-0.07, 0.06] | 0.99 | [0.93, 1.07] | 0.932 |
| VN-VAN | MA : RES | 0.10 | [-0.12, 0.32] | 1.11 | [0.88, 1.38] | 0.772 |
|  | VU : RES | 0.05 | [-0.17, 0.26] | 1.05 | [0.85, 1.29] | 0.965 |
|  | HC : RES | 0.01 | [-0.06, 0.07] | 1.01 | [0.94, 1.08] | 0.927 |
| VAN-Amygdala | MA : RES | -0.16 | [-0.37, 0.05] | 0.85 | [0.69, 1.05] | 0.538 |
|  | VU : RES | -0.21 | [-0.41, -0.01] | 0.81 | [0.66, 0.99] | 0.857 |
|  | HC : RES | -0.09 | [-0.16, -0.03] | 0.91 | [0.85, 0.97] | 0.095 |
| VAN-Hippocampus | MA : RES | -0.10 | [-0.32, 0.12] | 0.90 | [0.73, 1.12] | 0.772 |
|  | VU : RES | 0.02 | [-0.18, 0.23] | 1.02 | [0.83, 1.26] | 0.965 |
|  | HC : RES | 0.10 | [0.03, 0.16] | 1.10 | [1.03, 1.18] | 0.095 |
| VAN-VAN | MA : RES | 0.00 | [-0.21, 0.22] | 1.00 | [0.81, 1.25] | 0.987 |
|  | VU : RES | 0.13 | [-0.07, 0.33] | 1.13 | [0.93, 1.39] | 0.965 |
|  | HC : RES | 0.05 | [-0.01, 0.12] | 1.05 | [0.99, 1.13] | 0.456 |

*Note*. Abbreviations explained: auditory network (AN), Cingulo-parietal network (CPN), Cingulo-opecular network (CON), dorsal attention network (DAN), default mode network (DMN), frontal-parietal network (FPN), retrosplenial temporal network (RTN), salience network (SN), sensorimotor hand network (SMN[H]), sensorimotor mouth network (SMN[M]), visual network (VN), ventral attention network (VAN)

## Sex interaction effects

### Table S12. Internalizing group comparisons

| **Variables** | **Group comparisons** | **B** | **95% CI** | **Odds** | **95% CI** | ***p*FDR** |
| --- | --- | --- | --- | --- | --- | --- |
| AN-AN | MA : RES | -0.07 | [-0.37, 0.22] | 0.93 | [0.69, 1.25] | 0.871 |
|  | VU : RES | -0.23 | [-0.48, 0.02] | 0.80 | [0.62, 1.02] | 0.831 |
|  | HC : RES | -0.07 | [-0.23, 0.09] | 0.93 | [0.8, 1.09] | 0.861 |
| AN-Amygdala | MA : RES | 0.07 | [-0.22, 0.36] | 1.07 | [0.8, 1.44] | 0.871 |
|  | VU : RES | -0.07 | [-0.32, 0.18] | 0.93 | [0.73, 1.19] | 0.960 |
|  | HC : RES | 0.08 | [-0.08, 0.24] | 1.08 | [0.93, 1.27] | 0.838 |
| AN-CPN | MA : RES | -0.08 | [-0.37, 0.21] | 0.93 | [0.69, 1.24] | 0.871 |
|  | VU : RES | -0.06 | [-0.32, 0.19] | 0.94 | [0.73, 1.21] | 0.960 |
|  | HC : RES | -0.12 | [-0.28, 0.04] | 0.89 | [0.76, 1.04] | 0.714 |
| AN-CON | MA : RES | -0.10 | [-0.39, 0.19] | 0.91 | [0.67, 1.22] | 0.863 |
|  | VU : RES | -0.20 | [-0.45, 0.05] | 0.82 | [0.64, 1.05] | 0.831 |
|  | HC : RES | -0.05 | [-0.21, 0.11] | 0.95 | [0.81, 1.11] | 0.874 |
| AN-DAN | MA : RES | -0.05 | [-0.34, 0.23] | 0.95 | [0.71, 1.26] | 0.871 |
|  | VU : RES | -0.11 | [-0.35, 0.14] | 0.90 | [0.7, 1.15] | 0.911 |
|  | HC : RES | -0.01 | [-0.16, 0.15] | 0.99 | [0.85, 1.16] | 1.000 |
| AN-DMN | MA : RES | 0.34 | [0.05, 0.64] | 1.41 | [1.05, 1.89] | 0.267 |
|  | VU : RES | 0.22 | [-0.04, 0.47] | 1.24 | [0.97, 1.6] | 0.831 |
|  | HC : RES | 0.18 | [0.02, 0.34] | 1.20 | [1.02, 1.41] | 0.410 |
| AN-FPN | MA : RES | 0.21 | [-0.08, 0.5] | 1.23 | [0.92, 1.64] | 0.808 |
|  | VU : RES | 0.12 | [-0.13, 0.37] | 1.13 | [0.88, 1.45] | 0.911 |
|  | HC : RES | 0.17 | [0.01, 0.33] | 1.18 | [1.01, 1.38] | 0.410 |
| AN-hippocampus | MA : RES | 0.13 | [-0.16, 0.41] | 1.13 | [0.85, 1.51] | 0.829 |
|  | VU : RES | -0.15 | [-0.4, 0.09] | 0.86 | [0.67, 1.1] | 0.877 |
|  | HC : RES | -0.06 | [-0.21, 0.1] | 0.95 | [0.81, 1.1] | 0.874 |
| AN-RTN | MA : RES | -0.12 | [-0.42, 0.18] | 0.89 | [0.66, 1.2] | 0.829 |
|  | VU : RES | -0.12 | [-0.38, 0.13] | 0.88 | [0.69, 1.14] | 0.911 |
|  | HC : RES | -0.09 | [-0.25, 0.07] | 0.91 | [0.78, 1.07] | 0.838 |
| AN-SN | MA : RES | -0.11 | [-0.4, 0.18] | 0.89 | [0.67, 1.19] | 0.829 |
|  | VU : RES | -0.13 | [-0.37, 0.12] | 0.88 | [0.69, 1.13] | 0.911 |
|  | HC : RES | -0.02 | [-0.18, 0.14] | 0.98 | [0.84, 1.15] | 0.982 |
| AN-SMN(H) | MA : RES | 0.01 | [-0.29, 0.31] | 1.01 | [0.75, 1.37] | 0.966 |
|  | VU : RES | 0.01 | [-0.24, 0.26] | 1.01 | [0.79, 1.3] | 0.986 |
|  | HC : RES | -0.10 | [-0.26, 0.05] | 0.90 | [0.77, 1.06] | 0.811 |
| AN-SMN(M) | MA : RES | 0.03 | [-0.26, 0.32] | 1.03 | [0.77, 1.38] | 0.945 |
|  | VU : RES | -0.08 | [-0.33, 0.17] | 0.92 | [0.72, 1.18] | 0.960 |
|  | HC : RES | 0.03 | [-0.13, 0.18] | 1.03 | [0.88, 1.2] | 0.940 |
| AN-VN | MA : RES | -0.43 | [-0.72, -0.13] | 0.65 | [0.49, 0.88] | 0.088 |
|  | VU : RES | -0.16 | [-0.41, 0.09] | 0.85 | [0.66, 1.09] | 0.838 |
|  | HC : RES | -0.21 | [-0.37, -0.05] | 0.81 | [0.69, 0.95] | 0.292 |
| AN-VAN | MA : RES | 0.13 | [-0.17, 0.43] | 1.14 | [0.85, 1.53] | 0.829 |
|  | VU : RES | -0.16 | [-0.41, 0.09] | 0.85 | [0.66, 1.09] | 0.838 |
|  | HC : RES | -0.01 | [-0.17, 0.15] | 0.99 | [0.84, 1.16] | 1.000 |
| CPN-Amygdala | MA : RES | 0.00 | [-0.29, 0.29] | 1.00 | [0.75, 1.34] | 0.996 |
|  | VU : RES | -0.13 | [-0.38, 0.11] | 0.88 | [0.68, 1.12] | 0.911 |
|  | HC : RES | 0.07 | [-0.08, 0.23] | 1.07 | [0.92, 1.26] | 0.838 |
| CPN-CPN | MA : RES | 0.13 | [-0.16, 0.42] | 1.14 | [0.86, 1.53] | 0.829 |
|  | VU : RES | -0.04 | [-0.29, 0.2] | 0.96 | [0.75, 1.22] | 0.978 |
|  | HC : RES | -0.04 | [-0.19, 0.12] | 0.96 | [0.82, 1.13] | 0.925 |
| CPN-CON | MA : RES | -0.28 | [-0.58, 0.01] | 0.75 | [0.56, 1.01] | 0.405 |
|  | VU : RES | -0.19 | [-0.44, 0.06] | 0.83 | [0.64, 1.06] | 0.838 |
|  | HC : RES | -0.16 | [-0.32, 0] | 0.85 | [0.73, 1] | 0.455 |
| CPN-DAN | MA : RES | -0.48 | [-0.78, -0.18] | 0.62 | [0.46, 0.83] | 0.069 |
|  | VU : RES | -0.03 | [-0.28, 0.22] | 0.97 | [0.76, 1.24] | 0.978 |
|  | HC : RES | 0.01 | [-0.14, 0.17] | 1.01 | [0.87, 1.18] | 1.000 |
| CPN-DMN | MA : RES | 0.36 | [0.07, 0.66] | 1.44 | [1.07, 1.93] | 0.187 |
|  | VU : RES | 0.20 | [-0.04, 0.45] | 1.23 | [0.96, 1.57] | 0.831 |
|  | HC : RES | 0.19 | [0.03, 0.35] | 1.21 | [1.04, 1.42] | 0.410 |
| CPN-FPN | MA : RES | -0.07 | [-0.36, 0.22] | 0.93 | [0.69, 1.24] | 0.871 |
|  | VU : RES | 0.09 | [-0.16, 0.33] | 1.09 | [0.85, 1.4] | 0.960 |
|  | HC : RES | 0.06 | [-0.09, 0.22] | 1.07 | [0.91, 1.25] | 0.864 |
| CPN-Hippocampus | MA : RES | -0.01 | [-0.3, 0.28] | 0.99 | [0.74, 1.32] | 0.966 |
|  | VU : RES | -0.18 | [-0.42, 0.07] | 0.84 | [0.65, 1.07] | 0.838 |
|  | HC : RES | 0.03 | [-0.12, 0.19] | 1.03 | [0.88, 1.21] | 0.925 |
| CPN-RTN | MA : RES | 0.31 | [0.02, 0.6] | 1.37 | [1.02, 1.83] | 0.337 |
|  | VU : RES | 0.03 | [-0.22, 0.28] | 1.03 | [0.8, 1.32] | 0.978 |
|  | HC : RES | 0.01 | [-0.15, 0.16] | 1.01 | [0.86, 1.18] | 1.000 |
| CPN-SN | MA : RES | 0.03 | [-0.27, 0.32] | 1.03 | [0.76, 1.38] | 0.950 |
|  | VU : RES | 0.06 | [-0.19, 0.3] | 1.06 | [0.82, 1.36] | 0.978 |
|  | HC : RES | 0.00 | [-0.15, 0.16] | 1.00 | [0.86, 1.18] | 1.000 |
| CPN-SMN(H) | MA : RES | -0.09 | [-0.37, 0.2] | 0.92 | [0.69, 1.22] | 0.871 |
|  | VU : RES | 0.12 | [-0.13, 0.37] | 1.13 | [0.88, 1.44] | 0.911 |
|  | HC : RES | 0.01 | [-0.15, 0.16] | 1.01 | [0.86, 1.18] | 1.000 |
| CPN-SMN(M) | MA : RES | -0.12 | [-0.41, 0.16] | 0.89 | [0.67, 1.18] | 0.829 |
|  | VU : RES | -0.06 | [-0.31, 0.19] | 0.94 | [0.74, 1.2] | 0.960 |
|  | HC : RES | -0.11 | [-0.26, 0.05] | 0.90 | [0.77, 1.05] | 0.811 |
| CPN-VN | MA : RES | 0.03 | [-0.26, 0.32] | 1.03 | [0.77, 1.38] | 0.944 |
|  | VU : RES | -0.22 | [-0.46, 0.03] | 0.81 | [0.63, 1.03] | 0.831 |
|  | HC : RES | -0.07 | [-0.23, 0.08] | 0.93 | [0.8, 1.09] | 0.838 |
| CPN-VAN | MA : RES | 0.17 | [-0.12, 0.46] | 1.19 | [0.89, 1.59] | 0.812 |
|  | VU : RES | -0.08 | [-0.33, 0.17] | 0.92 | [0.72, 1.18] | 0.960 |
|  | HC : RES | -0.03 | [-0.19, 0.12] | 0.97 | [0.82, 1.13] | 0.925 |
| CON-Amygdala | MA : RES | 0.07 | [-0.22, 0.36] | 1.07 | [0.81, 1.43] | 0.871 |
|  | VU : RES | -0.01 | [-0.26, 0.23] | 0.99 | [0.77, 1.26] | 0.978 |
|  | HC : RES | 0.14 | [-0.01, 0.3] | 1.15 | [0.99, 1.35] | 0.553 |
| CON-CON | MA : RES | -0.03 | [-0.33, 0.26] | 0.97 | [0.72, 1.3] | 0.944 |
|  | VU : RES | -0.16 | [-0.41, 0.09] | 0.85 | [0.66, 1.09] | 0.838 |
|  | HC : RES | -0.06 | [-0.22, 0.1] | 0.94 | [0.81, 1.1] | 0.864 |
| CON-DAN | MA : RES | -0.18 | [-0.47, 0.11] | 0.84 | [0.63, 1.12] | 0.812 |
|  | VU : RES | 0.00 | [-0.25, 0.24] | 1.00 | [0.78, 1.27] | 0.986 |
|  | HC : RES | 0.03 | [-0.12, 0.19] | 1.03 | [0.88, 1.21] | 0.925 |
| CON-DMN | MA : RES | 0.21 | [-0.09, 0.5] | 1.23 | [0.91, 1.65] | 0.808 |
|  | VU : RES | 0.11 | [-0.14, 0.37] | 1.12 | [0.87, 1.44] | 0.911 |
|  | HC : RES | 0.06 | [-0.1, 0.22] | 1.06 | [0.91, 1.25] | 0.864 |
| CON-FPN | MA : RES | 0.10 | [-0.19, 0.39] | 1.10 | [0.83, 1.48] | 0.863 |
|  | VU : RES | -0.02 | [-0.27, 0.22] | 0.98 | [0.76, 1.25] | 0.978 |
|  | HC : RES | 0.06 | [-0.09, 0.22] | 1.07 | [0.91, 1.25] | 0.864 |
| CON-Hippocampus | MA : RES | -0.06 | [-0.34, 0.23] | 0.94 | [0.71, 1.26] | 0.871 |
|  | VU : RES | -0.08 | [-0.33, 0.17] | 0.92 | [0.72, 1.19] | 0.960 |
|  | HC : RES | 0.04 | [-0.11, 0.2] | 1.04 | [0.89, 1.22] | 0.907 |
| CON-RTN | MA : RES | -0.06 | [-0.35, 0.24] | 0.95 | [0.7, 1.27] | 0.871 |
|  | VU : RES | -0.01 | [-0.26, 0.24] | 0.99 | [0.77, 1.28] | 0.986 |
|  | HC : RES | -0.01 | [-0.17, 0.15] | 0.99 | [0.84, 1.16] | 1.000 |
| CON-SN | MA : RES | -0.06 | [-0.35, 0.23] | 0.94 | [0.7, 1.26] | 0.871 |
|  | VU : RES | -0.21 | [-0.46, 0.03] | 0.81 | [0.63, 1.04] | 0.831 |
|  | HC : RES | -0.10 | [-0.26, 0.06] | 0.91 | [0.77, 1.06] | 0.822 |
| CON-SMN(H) | MA : RES | -0.02 | [-0.32, 0.27] | 0.98 | [0.73, 1.32] | 0.961 |
|  | VU : RES | -0.02 | [-0.28, 0.23] | 0.98 | [0.76, 1.26] | 0.978 |
|  | HC : RES | -0.11 | [-0.27, 0.05] | 0.90 | [0.76, 1.05] | 0.811 |
| CON-SMN(M) | MA : RES | 0.10 | [-0.19, 0.4] | 1.11 | [0.83, 1.49] | 0.853 |
|  | VU : RES | 0.13 | [-0.11, 0.38] | 1.14 | [0.89, 1.46] | 0.911 |
|  | HC : RES | 0.05 | [-0.11, 0.2] | 1.05 | [0.89, 1.22] | 0.889 |
| CON-VN | MA : RES | -0.17 | [-0.46, 0.13] | 0.85 | [0.63, 1.13] | 0.812 |
|  | VU : RES | 0.03 | [-0.22, 0.28] | 1.03 | [0.81, 1.32] | 0.978 |
|  | HC : RES | -0.08 | [-0.23, 0.08] | 0.93 | [0.79, 1.08] | 0.838 |
| CON-VAN | MA : RES | 0.14 | [-0.16, 0.44] | 1.15 | [0.86, 1.55] | 0.829 |
|  | VU : RES | -0.17 | [-0.42, 0.08] | 0.84 | [0.65, 1.08] | 0.838 |
|  | HC : RES | 0.00 | [-0.16, 0.16] | 1.00 | [0.85, 1.17] | 1.000 |
| DAN-Amygdala | MA : RES | 0.11 | [-0.18, 0.4] | 1.11 | [0.83, 1.49] | 0.832 |
|  | VU : RES | 0.09 | [-0.16, 0.33] | 1.09 | [0.85, 1.39] | 0.960 |
|  | HC : RES | 0.15 | [-0.01, 0.3] | 1.16 | [0.99, 1.35] | 0.553 |
| DAN-DAN | MA : RES | -0.07 | [-0.36, 0.21] | 0.93 | [0.7, 1.24] | 0.871 |
|  | VU : RES | 0.03 | [-0.21, 0.28] | 1.03 | [0.81, 1.32] | 0.978 |
|  | HC : RES | 0.00 | [-0.16, 0.16] | 1.00 | [0.86, 1.17] | 1.000 |
| DAN-DMN | MA : RES | 0.06 | [-0.23, 0.35] | 1.06 | [0.79, 1.42] | 0.871 |
|  | VU : RES | 0.02 | [-0.22, 0.27] | 1.02 | [0.8, 1.31] | 0.978 |
|  | HC : RES | -0.09 | [-0.24, 0.07] | 0.92 | [0.78, 1.07] | 0.838 |
| DAN-FPN | MA : RES | -0.15 | [-0.44, 0.15] | 0.86 | [0.65, 1.16] | 0.829 |
|  | VU : RES | -0.06 | [-0.31, 0.18] | 0.94 | [0.73, 1.2] | 0.960 |
|  | HC : RES | -0.08 | [-0.24, 0.08] | 0.92 | [0.79, 1.08] | 0.838 |
| DAN-Hippocampus | MA : RES | -0.01 | [-0.31, 0.28] | 0.99 | [0.74, 1.32] | 0.966 |
|  | VU : RES | -0.02 | [-0.27, 0.23] | 0.98 | [0.77, 1.26] | 0.978 |
|  | HC : RES | -0.03 | [-0.18, 0.13] | 0.97 | [0.83, 1.14] | 0.940 |
| DAN-RTN | MA : RES | 0.25 | [-0.04, 0.54] | 1.29 | [0.96, 1.72] | 0.552 |
|  | VU : RES | 0.14 | [-0.11, 0.39] | 1.15 | [0.9, 1.47] | 0.905 |
|  | HC : RES | -0.04 | [-0.2, 0.11] | 0.96 | [0.82, 1.12] | 0.904 |
| DAN-SN | MA : RES | -0.12 | [-0.4, 0.17] | 0.89 | [0.67, 1.19] | 0.829 |
|  | VU : RES | 0.14 | [-0.11, 0.39] | 1.15 | [0.9, 1.47] | 0.905 |
|  | HC : RES | 0.05 | [-0.1, 0.21] | 1.05 | [0.9, 1.23] | 0.874 |
| DAN-SMN(H) | MA : RES | 0.08 | [-0.2, 0.37] | 1.09 | [0.82, 1.44] | 0.871 |
|  | VU : RES | 0.04 | [-0.2, 0.29] | 1.04 | [0.82, 1.33] | 0.978 |
|  | HC : RES | -0.07 | [-0.23, 0.08] | 0.93 | [0.8, 1.09] | 0.838 |
| DAN-SMN(M) | MA : RES | -0.12 | [-0.4, 0.17] | 0.89 | [0.67, 1.18] | 0.829 |
|  | VU : RES | -0.05 | [-0.3, 0.19] | 0.95 | [0.74, 1.21] | 0.978 |
|  | HC : RES | -0.03 | [-0.18, 0.12] | 0.97 | [0.83, 1.13] | 0.925 |
| DAN-VN | MA : RES | 0.03 | [-0.26, 0.32] | 1.03 | [0.77, 1.37] | 0.950 |
|  | VU : RES | 0.09 | [-0.16, 0.34] | 1.09 | [0.86, 1.4] | 0.960 |
|  | HC : RES | 0.08 | [-0.08, 0.24] | 1.08 | [0.93, 1.27] | 0.838 |
| DAN-VAN | MA : RES | 0.07 | [-0.23, 0.36] | 1.07 | [0.79, 1.44] | 0.871 |
|  | VU : RES | 0.06 | [-0.18, 0.31] | 1.07 | [0.83, 1.37] | 0.960 |
|  | HC : RES | 0.00 | [-0.16, 0.16] | 1.00 | [0.85, 1.17] | 1.000 |
| DMN-Amygdala | MA : RES | -0.08 | [-0.37, 0.21] | 0.93 | [0.69, 1.24] | 0.871 |
|  | VU : RES | -0.02 | [-0.27, 0.23] | 0.98 | [0.76, 1.26] | 0.978 |
|  | HC : RES | 0.04 | [-0.12, 0.2] | 1.04 | [0.89, 1.22] | 0.924 |
| DMN-DMN | MA : RES | 0.13 | [-0.16, 0.42] | 1.14 | [0.85, 1.53] | 0.829 |
|  | VU : RES | 0.04 | [-0.21, 0.28] | 1.04 | [0.81, 1.33] | 0.978 |
|  | HC : RES | 0.00 | [-0.16, 0.15] | 1.00 | [0.85, 1.17] | 1.000 |
| DMN-FPN | MA : RES | -0.09 | [-0.38, 0.2] | 0.91 | [0.68, 1.22] | 0.871 |
|  | VU : RES | -0.02 | [-0.26, 0.23] | 0.99 | [0.77, 1.26] | 0.978 |
|  | HC : RES | -0.06 | [-0.22, 0.1] | 0.94 | [0.81, 1.1] | 0.874 |
| DMN-Hippocampus | MA : RES | -0.07 | [-0.36, 0.23] | 0.94 | [0.7, 1.26] | 0.871 |
|  | VU : RES | -0.24 | [-0.49, 0.01] | 0.79 | [0.61, 1.01] | 0.831 |
|  | HC : RES | -0.11 | [-0.27, 0.05] | 0.90 | [0.77, 1.05] | 0.811 |
| DMN-RTN | MA : RES | 0.07 | [-0.22, 0.37] | 1.08 | [0.8, 1.45] | 0.871 |
|  | VU : RES | 0.09 | [-0.17, 0.34] | 1.09 | [0.85, 1.4] | 0.960 |
|  | HC : RES | 0.09 | [-0.07, 0.25] | 1.09 | [0.93, 1.28] | 0.838 |
| DMN-SN | MA : RES | 0.18 | [-0.11, 0.48] | 1.20 | [0.9, 1.61] | 0.812 |
|  | VU : RES | -0.09 | [-0.34, 0.15] | 0.91 | [0.71, 1.17] | 0.960 |
|  | HC : RES | 0.00 | [-0.16, 0.16] | 1.00 | [0.86, 1.17] | 1.000 |
| DMN-SMN(H) | MA : RES | 0.31 | [0.01, 0.6] | 1.36 | [1.01, 1.83] | 0.337 |
|  | VU : RES | 0.04 | [-0.22, 0.29] | 1.04 | [0.81, 1.33] | 0.978 |
|  | HC : RES | 0.17 | [0.01, 0.33] | 1.19 | [1.01, 1.39] | 0.410 |
| DMN-SMN(M) | MA : RES | 0.12 | [-0.17, 0.41] | 1.13 | [0.84, 1.51] | 0.829 |
|  | VU : RES | 0.02 | [-0.23, 0.27] | 1.02 | [0.8, 1.31] | 0.978 |
|  | HC : RES | 0.04 | [-0.12, 0.19] | 1.04 | [0.89, 1.21] | 0.925 |
| DMN-VN | MA : RES | -0.14 | [-0.43, 0.15] | 0.87 | [0.65, 1.16] | 0.829 |
|  | VU : RES | -0.12 | [-0.37, 0.12] | 0.88 | [0.69, 1.13] | 0.911 |
|  | HC : RES | -0.01 | [-0.16, 0.15] | 0.99 | [0.85, 1.16] | 1.000 |
| DMN-VAN | MA : RES | 0.17 | [-0.12, 0.46] | 1.18 | [0.88, 1.58] | 0.812 |
|  | VU : RES | 0.02 | [-0.23, 0.26] | 1.02 | [0.8, 1.3] | 0.978 |
|  | HC : RES | 0.08 | [-0.08, 0.23] | 1.08 | [0.92, 1.26] | 0.838 |
| FPN-Amygdala | MA : RES | 0.11 | [-0.18, 0.4] | 1.12 | [0.83, 1.5] | 0.829 |
|  | VU : RES | -0.15 | [-0.4, 0.1] | 0.86 | [0.67, 1.11] | 0.882 |
|  | HC : RES | 0.13 | [-0.03, 0.29] | 1.14 | [0.97, 1.33] | 0.636 |
| FPN-FPN | MA : RES | -0.18 | [-0.47, 0.11] | 0.83 | [0.63, 1.11] | 0.812 |
|  | VU : RES | -0.08 | [-0.33, 0.17] | 0.92 | [0.72, 1.18] | 0.960 |
|  | HC : RES | -0.17 | [-0.33, -0.02] | 0.84 | [0.72, 0.98] | 0.410 |
| FPN-Hippocampus | MA : RES | 0.21 | [-0.09, 0.5] | 1.23 | [0.92, 1.65] | 0.808 |
|  | VU : RES | 0.11 | [-0.14, 0.36] | 1.12 | [0.87, 1.43] | 0.911 |
|  | HC : RES | 0.16 | [0, 0.32] | 1.17 | [1, 1.37] | 0.455 |
| FPN-RTN | MA : RES | 0.14 | [-0.16, 0.43] | 1.15 | [0.86, 1.54] | 0.829 |
|  | VU : RES | 0.07 | [-0.18, 0.32] | 1.07 | [0.84, 1.38] | 0.960 |
|  | HC : RES | 0.06 | [-0.09, 0.22] | 1.07 | [0.91, 1.25] | 0.864 |
| FPN-SN | MA : RES | -0.06 | [-0.35, 0.24] | 0.94 | [0.7, 1.27] | 0.871 |
|  | VU : RES | 0.07 | [-0.18, 0.32] | 1.08 | [0.84, 1.38] | 0.960 |
|  | HC : RES | -0.03 | [-0.18, 0.13] | 0.97 | [0.83, 1.14] | 0.943 |
| FPN-SMN(H) | MA : RES | 0.45 | [0.15, 0.74] | 1.56 | [1.17, 2.09] | 0.072 |
|  | VU : RES | 0.19 | [-0.06, 0.44] | 1.21 | [0.94, 1.56] | 0.838 |
|  | HC : RES | 0.23 | [0.07, 0.39] | 1.26 | [1.08, 1.48] | 0.205 |
| FPN-SMN(M) | MA : RES | 0.08 | [-0.21, 0.37] | 1.08 | [0.81, 1.44] | 0.871 |
|  | VU : RES | 0.22 | [-0.03, 0.46] | 1.24 | [0.97, 1.59] | 0.831 |
|  | HC : RES | 0.11 | [-0.05, 0.26] | 1.11 | [0.95, 1.3] | 0.811 |
| FPN-VN | MA : RES | 0.19 | [-0.09, 0.48] | 1.21 | [0.91, 1.61] | 0.808 |
|  | VU : RES | 0.06 | [-0.18, 0.31] | 1.07 | [0.83, 1.36] | 0.960 |
|  | HC : RES | -0.02 | [-0.18, 0.13] | 0.98 | [0.84, 1.14] | 0.945 |
| FPN-VAN | MA : RES | 0.24 | [-0.05, 0.53] | 1.27 | [0.95, 1.7] | 0.669 |
|  | VU : RES | -0.04 | [-0.29, 0.21] | 0.96 | [0.75, 1.23] | 0.978 |
|  | HC : RES | 0.05 | [-0.1, 0.21] | 1.06 | [0.9, 1.24] | 0.874 |
| RTN-Amygdala | MA : RES | 0.00 | [-0.29, 0.29] | 1.00 | [0.75, 1.33] | 0.996 |
|  | VU : RES | 0.08 | [-0.16, 0.33] | 1.08 | [0.85, 1.39] | 0.960 |
|  | HC : RES | -0.04 | [-0.2, 0.11] | 0.96 | [0.82, 1.12] | 0.889 |
| RTN-Hippocampus | MA : RES | 0.49 | [0.2, 0.78] | 1.63 | [1.22, 2.17] | 0.069 |
|  | VU : RES | 0.08 | [-0.17, 0.33] | 1.08 | [0.85, 1.39] | 0.960 |
|  | HC : RES | 0.07 | [-0.08, 0.23] | 1.07 | [0.92, 1.26] | 0.838 |
| RTN-RTN | MA : RES | 0.01 | [-0.28, 0.3] | 1.01 | [0.76, 1.35] | 0.966 |
|  | VU : RES | -0.15 | [-0.4, 0.1] | 0.86 | [0.67, 1.1] | 0.882 |
|  | HC : RES | -0.13 | [-0.29, 0.03] | 0.88 | [0.75, 1.03] | 0.636 |
| RTN-SN | MA : RES | 0.17 | [-0.12, 0.46] | 1.18 | [0.88, 1.58] | 0.812 |
|  | VU : RES | 0.11 | [-0.14, 0.36] | 1.12 | [0.87, 1.43] | 0.911 |
|  | HC : RES | 0.05 | [-0.11, 0.2] | 1.05 | [0.89, 1.22] | 0.889 |
| RTN-SMN(H) | MA : RES | -0.04 | [-0.33, 0.25] | 0.96 | [0.72, 1.28] | 0.917 |
|  | VU : RES | 0.00 | [-0.25, 0.25] | 1.00 | [0.78, 1.29] | 0.986 |
|  | HC : RES | -0.08 | [-0.24, 0.08] | 0.92 | [0.79, 1.08] | 0.838 |
| RTN-SMN(M) | MA : RES | -0.37 | [-0.67, -0.08] | 0.69 | [0.51, 0.92] | 0.174 |
|  | VU : RES | -0.24 | [-0.49, 0.01] | 0.79 | [0.61, 1.01] | 0.831 |
|  | HC : RES | -0.14 | [-0.3, 0.02] | 0.87 | [0.74, 1.02] | 0.559 |
| RTN-VN | MA : RES | -0.12 | [-0.43, 0.18] | 0.88 | [0.65, 1.2] | 0.829 |
|  | VU : RES | -0.21 | [-0.46, 0.05] | 0.81 | [0.63, 1.05] | 0.831 |
|  | HC : RES | -0.05 | [-0.21, 0.11] | 0.95 | [0.81, 1.12] | 0.889 |
| RTN-VAN | MA : RES | -0.01 | [-0.31, 0.28] | 0.99 | [0.74, 1.33] | 0.966 |
|  | VU : RES | 0.04 | [-0.21, 0.29] | 1.04 | [0.81, 1.34] | 0.978 |
|  | HC : RES | 0.03 | [-0.12, 0.19] | 1.03 | [0.88, 1.21] | 0.925 |
| SN-Amygdala | MA : RES | 0.41 | [0.12, 0.7] | 1.51 | [1.13, 2.01] | 0.097 |
|  | VU : RES | 0.17 | [-0.08, 0.41] | 1.18 | [0.92, 1.51] | 0.838 |
|  | HC : RES | -0.05 | [-0.21, 0.11] | 0.95 | [0.81, 1.11] | 0.876 |
| SN-Hippocampus | MA : RES | -0.01 | [-0.3, 0.28] | 0.99 | [0.74, 1.32] | 0.966 |
|  | VU : RES | -0.04 | [-0.29, 0.2] | 0.96 | [0.75, 1.22] | 0.978 |
|  | HC : RES | -0.05 | [-0.21, 0.1] | 0.95 | [0.81, 1.11] | 0.874 |
| SN-SN | MA : RES | 0.12 | [-0.18, 0.41] | 1.12 | [0.84, 1.51] | 0.829 |
|  | VU : RES | -0.21 | [-0.46, 0.04] | 0.81 | [0.63, 1.04] | 0.831 |
|  | HC : RES | 0.00 | [-0.16, 0.16] | 1.00 | [0.86, 1.18] | 1.000 |
| SN-SMN(H) | MA : RES | 0.05 | [-0.24, 0.34] | 1.05 | [0.79, 1.41] | 0.871 |
|  | VU : RES | 0.02 | [-0.23, 0.27] | 1.02 | [0.79, 1.31] | 0.978 |
|  | HC : RES | 0.03 | [-0.13, 0.19] | 1.03 | [0.88, 1.21] | 0.925 |
| SN-SMN(M) | MA : RES | 0.03 | [-0.26, 0.32] | 1.03 | [0.77, 1.38] | 0.945 |
|  | VU : RES | 0.24 | [0, 0.49] | 1.28 | [1, 1.64] | 0.831 |
|  | HC : RES | 0.07 | [-0.09, 0.22] | 1.07 | [0.91, 1.25] | 0.861 |
| SN-VN | MA : RES | 0.08 | [-0.21, 0.38] | 1.09 | [0.81, 1.46] | 0.871 |
|  | VU : RES | 0.09 | [-0.15, 0.34] | 1.10 | [0.86, 1.41] | 0.960 |
|  | HC : RES | 0.10 | [-0.06, 0.26] | 1.10 | [0.94, 1.29] | 0.811 |
| SN-VAN | MA : RES | 0.30 | [0.01, 0.59] | 1.35 | [1.01, 1.8] | 0.337 |
|  | VU : RES | -0.26 | [-0.51, -0.01] | 0.77 | [0.6, 0.99] | 0.831 |
|  | HC : RES | -0.01 | [-0.17, 0.14] | 0.99 | [0.84, 1.15] | 1.000 |
| SMN(H)-Amygdala | MA : RES | 0.20 | [-0.09, 0.5] | 1.23 | [0.92, 1.64] | 0.808 |
|  | VU : RES | 0.16 | [-0.08, 0.41] | 1.18 | [0.92, 1.51] | 0.838 |
|  | HC : RES | 0.05 | [-0.11, 0.2] | 1.05 | [0.89, 1.22] | 0.889 |
| SMN(H)-Hippocampus | MA : RES | 0.20 | [-0.09, 0.49] | 1.22 | [0.91, 1.63] | 0.808 |
|  | VU : RES | 0.00 | [-0.25, 0.24] | 1.00 | [0.78, 1.28] | 0.986 |
|  | HC : RES | 0.08 | [-0.08, 0.23] | 1.08 | [0.92, 1.26] | 0.838 |
| SMN(H)-SMN(H) | MA : RES | -0.15 | [-0.45, 0.14] | 0.86 | [0.64, 1.15] | 0.829 |
|  | VU : RES | -0.17 | [-0.42, 0.09] | 0.85 | [0.66, 1.09] | 0.838 |
|  | HC : RES | -0.25 | [-0.41, -0.1] | 0.78 | [0.66, 0.91] | 0.163 |
| SMN(H)-SMN(M) | MA : RES | -0.07 | [-0.36, 0.22] | 0.94 | [0.7, 1.25] | 0.871 |
|  | VU : RES | -0.01 | [-0.26, 0.23] | 0.99 | [0.77, 1.26] | 0.978 |
|  | HC : RES | -0.13 | [-0.28, 0.03] | 0.88 | [0.75, 1.03] | 0.636 |
| SMN(H)-VN | MA : RES | -0.46 | [-0.75, -0.17] | 0.63 | [0.47, 0.85] | 0.069 |
|  | VU : RES | 0.02 | [-0.23, 0.27] | 1.02 | [0.8, 1.31] | 0.978 |
|  | HC : RES | -0.18 | [-0.34, -0.02] | 0.83 | [0.71, 0.98] | 0.410 |
| SMN(H)-VAN | MA : RES | 0.17 | [-0.12, 0.46] | 1.18 | [0.88, 1.58] | 0.812 |
|  | VU : RES | 0.00 | [-0.25, 0.25] | 1.00 | [0.78, 1.29] | 0.986 |
|  | HC : RES | 0.06 | [-0.09, 0.22] | 1.07 | [0.91, 1.25] | 0.864 |
| SMN(M)-Amygdala | MA : RES | 0.28 | [-0.01, 0.57] | 1.32 | [0.99, 1.76] | 0.405 |
|  | VU : RES | 0.12 | [-0.13, 0.37] | 1.13 | [0.88, 1.44] | 0.911 |
|  | HC : RES | 0.10 | [-0.06, 0.26] | 1.10 | [0.94, 1.29] | 0.811 |
| SMN(M)-Hippocampus | MA : RES | 0.14 | [-0.16, 0.43] | 1.15 | [0.85, 1.54] | 0.829 |
|  | VU : RES | 0.04 | [-0.21, 0.29] | 1.04 | [0.81, 1.34] | 0.978 |
|  | HC : RES | -0.01 | [-0.17, 0.15] | 0.99 | [0.85, 1.17] | 1.000 |
| SMN(M)-SMN(M) | MA : RES | 0.09 | [-0.2, 0.38] | 1.09 | [0.82, 1.46] | 0.871 |
|  | VU : RES | -0.09 | [-0.34, 0.15] | 0.91 | [0.71, 1.17] | 0.960 |
|  | HC : RES | 0.13 | [-0.03, 0.28] | 1.14 | [0.97, 1.33] | 0.636 |
| SMN(M)-VN | MA : RES | -0.31 | [-0.6, -0.02] | 0.73 | [0.55, 0.98] | 0.337 |
|  | VU : RES | -0.08 | [-0.32, 0.17] | 0.93 | [0.72, 1.19] | 0.960 |
|  | HC : RES | -0.09 | [-0.24, 0.07] | 0.92 | [0.78, 1.07] | 0.838 |
| SMN(M)-VAN | MA : RES | 0.19 | [-0.1, 0.48] | 1.21 | [0.91, 1.62] | 0.808 |
|  | VU : RES | -0.01 | [-0.26, 0.24] | 0.99 | [0.77, 1.27] | 0.986 |
|  | HC : RES | 0.08 | [-0.08, 0.23] | 1.08 | [0.92, 1.26] | 0.838 |
| VN-Amygdala | MA : RES | -0.19 | [-0.49, 0.1] | 0.82 | [0.61, 1.11] | 0.808 |
|  | VU : RES | -0.11 | [-0.36, 0.14] | 0.90 | [0.7, 1.15] | 0.911 |
|  | HC : RES | 0.02 | [-0.14, 0.18] | 1.02 | [0.87, 1.2] | 0.945 |
| VN-Hippocampus | MA : RES | -0.12 | [-0.41, 0.17] | 0.89 | [0.66, 1.19] | 0.829 |
|  | VU : RES | -0.24 | [-0.49, 0.01] | 0.79 | [0.61, 1.01] | 0.831 |
|  | HC : RES | 0.02 | [-0.13, 0.18] | 1.02 | [0.88, 1.2] | 0.943 |
| VN-VN | MA : RES | 0.12 | [-0.17, 0.42] | 1.13 | [0.84, 1.52] | 0.829 |
|  | VU : RES | 0.02 | [-0.23, 0.28] | 1.02 | [0.79, 1.32] | 0.978 |
|  | HC : RES | 0.09 | [-0.07, 0.25] | 1.09 | [0.93, 1.28] | 0.838 |
| VN-VAN | MA : RES | -0.14 | [-0.43, 0.15] | 0.87 | [0.65, 1.16] | 0.829 |
|  | VU : RES | 0.06 | [-0.19, 0.31] | 1.06 | [0.83, 1.36] | 0.960 |
|  | HC : RES | -0.03 | [-0.19, 0.12] | 0.97 | [0.83, 1.13] | 0.925 |
| VAN-Amygdala | MA : RES | 0.13 | [-0.16, 0.42] | 1.14 | [0.85, 1.53] | 0.829 |
|  | VU : RES | -0.02 | [-0.27, 0.23] | 0.98 | [0.76, 1.26] | 0.978 |
|  | HC : RES | -0.02 | [-0.18, 0.14] | 0.98 | [0.84, 1.15] | 0.999 |
| VAN-Hippocampus | MA : RES | 0.05 | [-0.24, 0.35] | 1.06 | [0.79, 1.42] | 0.871 |
|  | VU : RES | 0.11 | [-0.14, 0.36] | 1.12 | [0.87, 1.44] | 0.911 |
|  | HC : RES | 0.11 | [-0.05, 0.27] | 1.12 | [0.95, 1.31] | 0.811 |
| VAN-VAN | MA : RES | 0.03 | [-0.26, 0.33] | 1.03 | [0.77, 1.39] | 0.944 |
|  | VU : RES | -0.18 | [-0.43, 0.07] | 0.84 | [0.65, 1.07] | 0.838 |
|  | HC : RES | 0.06 | [-0.1, 0.21] | 1.06 | [0.9, 1.24] | 0.874 |

*Note*. Abbreviations explained: auditory network (AN), Cingulo-parietal network (CPN), Cingulo-opecular network (CON), dorsal attention network (DAN), default mode network (DMN), frontal-parietal network (FPN), retrosplenial temporal network (RTN), salience network (SN), sensorimotor hand network (SMN[H]), sensorimotor mouth network (SMN[M]), visual network (VN), ventral attention network (VAN)

### Table S13. Externalizing group comparisons

| **Variables** | **Group comparisons** | **B** | **95% CI** | **Odds** | **95% CI** | ***p*FDR** |
| --- | --- | --- | --- | --- | --- | --- |
| AN-AN | MA : RES | -0.03 | [-0.45, 0.4] | 0.97 | [0.64, 1.48] | 0.963 |
|  | VU : RES | 0.04 | [-0.39, 0.47] | 1.04 | [0.68, 1.59] | 0.963 |
|  | HC : RES | -0.06 | [-0.48, 0.37] | 0.94 | [0.62, 1.44] | 0.963 |
| AN-Amygdala | MA : RES | 0.13 | [-0.3, 0.55] | 1.13 | [0.74, 1.73] | 0.963 |
|  | VU : RES | 0.25 | [-0.17, 0.66] | 1.28 | [0.85, 1.94] | 0.963 |
|  | HC : RES | 0.41 | [0.01, 0.82] | 1.51 | [1.01, 2.26] | 0.936 |
| AN-CPN | MA : RES | -0.09 | [-0.51, 0.33] | 0.92 | [0.6, 1.39] | 0.963 |
|  | VU : RES | -0.04 | [-0.46, 0.37] | 0.96 | [0.63, 1.45] | 0.963 |
|  | HC : RES | -0.07 | [-0.52, 0.37] | 0.93 | [0.6, 1.45] | 0.963 |
| AN-CON | MA : RES | -0.27 | [-0.7, 0.16] | 0.76 | [0.49, 1.17] | 0.963 |
|  | VU : RES | 0.13 | [-0.31, 0.56] | 1.13 | [0.74, 1.75] | 0.963 |
|  | HC : RES | -0.37 | [-0.79, 0.05] | 0.69 | [0.46, 1.05] | 0.936 |
| AN-DAN | MA : RES | 0.14 | [-0.28, 0.57] | 1.16 | [0.75, 1.77] | 0.963 |
|  | VU : RES | 0.14 | [-0.29, 0.57] | 1.15 | [0.75, 1.76] | 0.963 |
|  | HC : RES | 0.28 | [-0.14, 0.7] | 1.32 | [0.87, 2.01] | 0.963 |
| AN-DMN | MA : RES | 0.36 | [-0.06, 0.78] | 1.44 | [0.94, 2.19] | 0.936 |
|  | VU : RES | -0.14 | [-0.57, 0.29] | 0.87 | [0.57, 1.33] | 0.963 |
|  | HC : RES | 0.04 | [-0.38, 0.45] | 1.04 | [0.69, 1.56] | 0.963 |
| AN-FPN | MA : RES | 0.21 | [-0.22, 0.63] | 1.23 | [0.8, 1.88] | 0.963 |
|  | VU : RES | -0.07 | [-0.5, 0.37] | 0.94 | [0.61, 1.44] | 0.963 |
|  | HC : RES | 0.10 | [-0.34, 0.53] | 1.10 | [0.71, 1.7] | 0.963 |
| AN-hippocampus | MA : RES | -0.28 | [-0.7, 0.15] | 0.76 | [0.5, 1.16] | 0.963 |
|  | VU : RES | 0.07 | [-0.35, 0.49] | 1.07 | [0.7, 1.64] | 0.963 |
|  | HC : RES | -0.39 | [-0.8, 0.03] | 0.68 | [0.45, 1.03] | 0.936 |
| AN-RTN | MA : RES | -0.07 | [-0.5, 0.36] | 0.93 | [0.61, 1.43] | 0.963 |
|  | VU : RES | 0.41 | [-0.02, 0.84] | 1.51 | [0.98, 2.31] | 0.936 |
|  | HC : RES | 0.14 | [-0.28, 0.56] | 1.15 | [0.76, 1.76] | 0.963 |
| AN-SN | MA : RES | 0.07 | [-0.36, 0.5] | 1.07 | [0.7, 1.64] | 0.963 |
|  | VU : RES | 0.15 | [-0.27, 0.57] | 1.16 | [0.76, 1.77] | 0.963 |
|  | HC : RES | 0.18 | [-0.24, 0.59] | 1.19 | [0.79, 1.81] | 0.963 |
| AN-SMN(H) | MA : RES | -0.03 | [-0.45, 0.39] | 0.97 | [0.64, 1.48] | 0.963 |
|  | VU : RES | -0.02 | [-0.44, 0.39] | 0.98 | [0.65, 1.48] | 0.968 |
|  | HC : RES | 0.06 | [-0.36, 0.49] | 1.07 | [0.7, 1.63] | 0.963 |
| AN-SMN(M) | MA : RES | -0.01 | [-0.44, 0.42] | 0.99 | [0.64, 1.52] | 0.972 |
|  | VU : RES | 0.10 | [-0.31, 0.52] | 1.11 | [0.73, 1.68] | 0.963 |
|  | HC : RES | -0.10 | [-0.54, 0.33] | 0.90 | [0.59, 1.39] | 0.963 |
| AN-VN | MA : RES | 0.09 | [-0.34, 0.51] | 1.09 | [0.71, 1.67] | 0.963 |
|  | VU : RES | 0.11 | [-0.31, 0.54] | 1.12 | [0.73, 1.71] | 0.963 |
|  | HC : RES | 0.38 | [-0.04, 0.8] | 1.46 | [0.96, 2.23] | 0.936 |
| AN-VAN | MA : RES | 0.10 | [-0.32, 0.53] | 1.11 | [0.72, 1.7] | 0.963 |
|  | VU : RES | 0.12 | [-0.31, 0.55] | 1.12 | [0.73, 1.73] | 0.963 |
|  | HC : RES | 0.11 | [-0.31, 0.53] | 1.11 | [0.73, 1.69] | 0.963 |
| CPN-Amygdala | MA : RES | -0.13 | [-0.55, 0.29] | 0.88 | [0.58, 1.34] | 0.963 |
|  | VU : RES | -0.14 | [-0.57, 0.29] | 0.87 | [0.57, 1.34] | 0.963 |
|  | HC : RES | 0.28 | [-0.15, 0.71] | 1.32 | [0.86, 2.02] | 0.963 |
| CPN-CPN | MA : RES | 0.32 | [-0.1, 0.75] | 1.38 | [0.91, 2.11] | 0.963 |
|  | VU : RES | 0.12 | [-0.29, 0.54] | 1.13 | [0.75, 1.71] | 0.963 |
|  | HC : RES | 0.10 | [-0.32, 0.53] | 1.11 | [0.73, 1.69] | 0.963 |
| CPN-CON | MA : RES | -0.04 | [-0.47, 0.39] | 0.96 | [0.62, 1.47] | 0.963 |
|  | VU : RES | -0.06 | [-0.48, 0.36] | 0.94 | [0.62, 1.43] | 0.963 |
|  | HC : RES | -0.17 | [-0.58, 0.24] | 0.84 | [0.56, 1.27] | 0.963 |
| CPN-DAN | MA : RES | 0.03 | [-0.39, 0.45] | 1.03 | [0.67, 1.58] | 0.963 |
|  | VU : RES | 0.17 | [-0.25, 0.58] | 1.18 | [0.78, 1.79] | 0.963 |
|  | HC : RES | 0.17 | [-0.22, 0.57] | 1.19 | [0.8, 1.76] | 0.963 |
| CPN-DMN | MA : RES | 0.65 | [0.23, 1.07] | 1.92 | [1.26, 2.92] | 0.176 |
|  | VU : RES | 0.23 | [-0.19, 0.65] | 1.26 | [0.83, 1.91] | 0.963 |
|  | HC : RES | 0.08 | [-0.34, 0.5] | 1.08 | [0.71, 1.65] | 0.963 |
| CPN-FPN | MA : RES | 0.11 | [-0.3, 0.53] | 1.12 | [0.74, 1.71] | 0.963 |
|  | VU : RES | 0.07 | [-0.35, 0.49] | 1.08 | [0.71, 1.64] | 0.963 |
|  | HC : RES | 0.09 | [-0.34, 0.51] | 1.09 | [0.71, 1.67] | 0.963 |
| CPN-Hippocampus | MA : RES | -0.11 | [-0.53, 0.31] | 0.90 | [0.59, 1.37] | 0.963 |
|  | VU : RES | 0.04 | [-0.39, 0.48] | 1.05 | [0.68, 1.62] | 0.963 |
|  | HC : RES | 0.31 | [-0.11, 0.74] | 1.37 | [0.89, 2.09] | 0.963 |
| CPN-RTN | MA : RES | 0.05 | [-0.37, 0.47] | 1.05 | [0.69, 1.6] | 0.963 |
|  | VU : RES | 0.19 | [-0.22, 0.59] | 1.20 | [0.8, 1.81] | 0.963 |
|  | HC : RES | 0.33 | [-0.09, 0.75] | 1.40 | [0.92, 2.12] | 0.963 |
| CPN-SN | MA : RES | 0.21 | [-0.21, 0.63] | 1.23 | [0.81, 1.87] | 0.963 |
|  | VU : RES | 0.08 | [-0.34, 0.51] | 1.09 | [0.71, 1.66] | 0.963 |
|  | HC : RES | -0.21 | [-0.64, 0.21] | 0.81 | [0.53, 1.23] | 0.963 |
| CPN-SMN(H) | MA : RES | -0.10 | [-0.53, 0.33] | 0.91 | [0.59, 1.4] | 0.963 |
|  | VU : RES | 0.02 | [-0.4, 0.43] | 1.02 | [0.67, 1.54] | 0.968 |
|  | HC : RES | -0.29 | [-0.7, 0.13] | 0.75 | [0.5, 1.13] | 0.963 |
| CPN-SMN(M) | MA : RES | -0.45 | [-0.87, -0.03] | 0.64 | [0.42, 0.98] | 0.936 |
|  | VU : RES | 0.28 | [-0.14, 0.7] | 1.32 | [0.87, 2.02] | 0.963 |
|  | HC : RES | -0.63 | [-1.05, -0.21] | 0.53 | [0.35, 0.81] | 0.176 |
| CPN-VN | MA : RES | 0.21 | [-0.22, 0.64] | 1.24 | [0.81, 1.89] | 0.963 |
|  | VU : RES | -0.04 | [-0.47, 0.4] | 0.96 | [0.63, 1.49] | 0.963 |
|  | HC : RES | -0.15 | [-0.57, 0.28] | 0.86 | [0.57, 1.32] | 0.963 |
| CPN-VAN | MA : RES | -0.05 | [-0.47, 0.36] | 0.95 | [0.63, 1.43] | 0.963 |
|  | VU : RES | -0.01 | [-0.43, 0.41] | 0.99 | [0.65, 1.51] | 0.972 |
|  | HC : RES | 0.25 | [-0.16, 0.66] | 1.28 | [0.85, 1.93] | 0.963 |
| CON-Amygdala | MA : RES | 0.05 | [-0.34, 0.44] | 1.05 | [0.71, 1.56] | 0.963 |
|  | VU : RES | 0.14 | [-0.28, 0.56] | 1.15 | [0.76, 1.75] | 0.963 |
|  | HC : RES | 0.26 | [-0.16, 0.68] | 1.30 | [0.85, 1.98] | 0.963 |
| CON-CON | MA : RES | -0.19 | [-0.59, 0.22] | 0.83 | [0.55, 1.24] | 0.963 |
|  | VU : RES | -0.19 | [-0.62, 0.23] | 0.83 | [0.54, 1.26] | 0.963 |
|  | HC : RES | 0.01 | [-0.41, 0.43] | 1.01 | [0.66, 1.53] | 0.972 |
| CON-DAN | MA : RES | -0.03 | [-0.45, 0.38] | 0.97 | [0.64, 1.47] | 0.963 |
|  | VU : RES | 0.25 | [-0.17, 0.68] | 1.29 | [0.84, 1.97] | 0.963 |
|  | HC : RES | -0.02 | [-0.44, 0.4] | 0.98 | [0.64, 1.49] | 0.968 |
| CON-DMN | MA : RES | -0.06 | [-0.47, 0.35] | 0.94 | [0.62, 1.42] | 0.963 |
|  | VU : RES | 0.23 | [-0.19, 0.65] | 1.26 | [0.83, 1.92] | 0.963 |
|  | HC : RES | 0.14 | [-0.28, 0.57] | 1.16 | [0.76, 1.76] | 0.963 |
| CON-FPN | MA : RES | -0.14 | [-0.56, 0.28] | 0.87 | [0.57, 1.32] | 0.963 |
|  | VU : RES | 0.39 | [-0.04, 0.81] | 1.48 | [0.96, 2.26] | 0.936 |
|  | HC : RES | -0.02 | [-0.43, 0.4] | 0.98 | [0.65, 1.49] | 0.968 |
| CON-Hippocampus | MA : RES | 0.19 | [-0.22, 0.6] | 1.21 | [0.8, 1.83] | 0.963 |
|  | VU : RES | 0.19 | [-0.22, 0.61] | 1.21 | [0.8, 1.83] | 0.963 |
|  | HC : RES | -0.23 | [-0.64, 0.19] | 0.80 | [0.53, 1.21] | 0.963 |
| CON-RTN | MA : RES | -0.30 | [-0.72, 0.12] | 0.74 | [0.49, 1.13] | 0.963 |
|  | VU : RES | -0.18 | [-0.61, 0.24] | 0.83 | [0.55, 1.27] | 0.963 |
|  | HC : RES | -0.27 | [-0.68, 0.14] | 0.77 | [0.51, 1.15] | 0.963 |
| CON-SN | MA : RES | 0.01 | [-0.39, 0.42] | 1.01 | [0.68, 1.52] | 1.000 |
|  | VU : RES | 0.19 | [-0.22, 0.61] | 1.21 | [0.8, 1.84] | 0.767 |
|  | HC : RES | 0.10 | [-0.31, 0.51] | 1.10 | [0.73, 1.67] | 0.897 |
| CON-SMN(H) | MA : RES | 0.41 | [-0.01, 0.82] | 1.50 | [0.99, 2.27] | 0.742 |
|  | VU : RES | -0.17 | [-0.58, 0.24] | 0.84 | [0.56, 1.27] | 0.772 |
|  | HC : RES | 0.17 | [-0.23, 0.57] | 1.19 | [0.79, 1.78] | 0.772 |
| CON-SMN(M) | MA : RES | -0.02 | [-0.43, 0.39] | 0.98 | [0.65, 1.48] | 1.000 |
|  | VU : RES | 0.37 | [-0.04, 0.78] | 1.45 | [0.96, 2.19] | 0.742 |
|  | HC : RES | 0.09 | [-0.34, 0.52] | 1.10 | [0.71, 1.69] | 0.924 |
| CON-VN | MA : RES | -0.24 | [-0.64, 0.16] | 0.78 | [0.53, 1.17] | 0.742 |
|  | VU : RES | 0.13 | [-0.29, 0.55] | 1.14 | [0.75, 1.73] | 0.854 |
|  | HC : RES | -0.26 | [-0.67, 0.16] | 0.77 | [0.51, 1.17] | 0.742 |
| CON-VAN | MA : RES | 0.28 | [-0.13, 0.7] | 1.32 | [0.87, 2.01] | 0.742 |
|  | VU : RES | -0.07 | [-0.49, 0.35] | 0.93 | [0.61, 1.41] | 0.925 |
|  | HC : RES | 0.21 | [-0.19, 0.62] | 1.24 | [0.82, 1.86] | 0.747 |
| DAN-Amygdala | MA : RES | -0.13 | [-0.54, 0.28] | 0.88 | [0.59, 1.32] | 0.854 |
|  | VU : RES | -0.08 | [-0.49, 0.33] | 0.92 | [0.61, 1.4] | 0.925 |
|  | HC : RES | -0.28 | [-0.69, 0.13] | 0.76 | [0.5, 1.14] | 0.742 |
| DAN-DAN | MA : RES | 0.25 | [-0.17, 0.66] | 1.28 | [0.85, 1.93] | 0.742 |
|  | VU : RES | 0.29 | [-0.12, 0.71] | 1.34 | [0.89, 2.03] | 0.742 |
|  | HC : RES | 0.14 | [-0.28, 0.56] | 1.15 | [0.76, 1.75] | 0.846 |
| DAN-DMN | MA : RES | 0.04 | [-0.37, 0.45] | 1.04 | [0.69, 1.57] | 0.966 |
|  | VU : RES | 0.28 | [-0.13, 0.7] | 1.33 | [0.88, 2.01] | 0.742 |
|  | HC : RES | -0.01 | [-0.41, 0.39] | 0.99 | [0.66, 1.48] | 1.000 |
| DAN-FPN | MA : RES | -0.05 | [-0.46, 0.36] | 0.95 | [0.63, 1.43] | 0.955 |
|  | VU : RES | 0.23 | [-0.17, 0.64] | 1.26 | [0.84, 1.9] | 0.742 |
|  | HC : RES | 0.35 | [-0.05, 0.76] | 1.42 | [0.95, 2.14] | 0.742 |
| DAN-Hippocampus | MA : RES | -0.32 | [-0.74, 0.09] | 0.72 | [0.48, 1.1] | 0.742 |
|  | VU : RES | 0.25 | [-0.16, 0.66] | 1.29 | [0.86, 1.94] | 0.742 |
|  | HC : RES | 0.10 | [-0.3, 0.51] | 1.11 | [0.74, 1.67] | 0.887 |
| DAN-RTN | MA : RES | 0.33 | [-0.08, 0.74] | 1.39 | [0.92, 2.09] | 0.742 |
|  | VU : RES | 0.20 | [-0.21, 0.61] | 1.22 | [0.81, 1.84] | 0.747 |
|  | HC : RES | -0.19 | [-0.6, 0.23] | 0.83 | [0.55, 1.25] | 0.767 |
| DAN-SN | MA : RES | -0.02 | [-0.43, 0.39] | 0.98 | [0.65, 1.48] | 1.000 |
|  | VU : RES | 0.08 | [-0.33, 0.5] | 1.09 | [0.72, 1.64] | 0.925 |
|  | HC : RES | 0.31 | [-0.1, 0.73] | 1.37 | [0.9, 2.08] | 0.742 |
| DAN-SMN(H) | MA : RES | 0.23 | [-0.18, 0.64] | 1.26 | [0.83, 1.9] | 0.742 |
|  | VU : RES | 0.05 | [-0.37, 0.46] | 1.05 | [0.69, 1.58] | 0.955 |
|  | HC : RES | 0.07 | [-0.34, 0.48] | 1.07 | [0.71, 1.61] | 0.925 |
| DAN-SMN(M) | MA : RES | -0.02 | [-0.43, 0.39] | 0.98 | [0.65, 1.47] | 1.000 |
|  | VU : RES | 0.12 | [-0.29, 0.53] | 1.12 | [0.74, 1.69] | 0.884 |
|  | HC : RES | -0.24 | [-0.64, 0.17] | 0.79 | [0.52, 1.18] | 0.742 |
| DAN-VN | MA : RES | 0.37 | [-0.03, 0.78] | 1.45 | [0.97, 2.17] | 0.742 |
|  | VU : RES | -0.24 | [-0.65, 0.18] | 0.79 | [0.52, 1.19] | 0.742 |
|  | HC : RES | 0.24 | [-0.18, 0.66] | 1.27 | [0.84, 1.93] | 0.742 |
| DAN-VAN | MA : RES | -0.46 | [-0.87, -0.04] | 0.63 | [0.42, 0.96] | 0.742 |
|  | VU : RES | 0.08 | [-0.33, 0.48] | 1.08 | [0.72, 1.62] | 0.925 |
|  | HC : RES | 0.38 | [-0.03, 0.79] | 1.46 | [0.97, 2.2] | 0.742 |
| DMN-Amygdala | MA : RES | -0.10 | [-0.51, 0.32] | 0.91 | [0.6, 1.37] | 0.897 |
|  | VU : RES | 0.32 | [-0.09, 0.73] | 1.38 | [0.91, 2.08] | 0.742 |
|  | HC : RES | 0.00 | [-0.41, 0.41] | 1.00 | [0.66, 1.51] | 1.000 |
| DMN-DMN | MA : RES | 0.21 | [-0.21, 0.62] | 1.23 | [0.81, 1.86] | 0.747 |
|  | VU : RES | -0.24 | [-0.64, 0.17] | 0.79 | [0.53, 1.18] | 0.742 |
|  | HC : RES | 0.29 | [-0.1, 0.69] | 1.34 | [0.9, 2] | 0.742 |
| DMN-FPN | MA : RES | 0.21 | [-0.2, 0.62] | 1.23 | [0.82, 1.85] | 0.747 |
|  | VU : RES | -0.01 | [-0.41, 0.4] | 0.99 | [0.66, 1.49] | 1.000 |
|  | HC : RES | 0.00 | [-0.41, 0.42] | 1.00 | [0.66, 1.51] | 1.000 |
| DMN-Hippocampus | MA : RES | 0.03 | [-0.38, 0.43] | 1.03 | [0.69, 1.54] | 1.000 |
|  | VU : RES | 0.32 | [-0.09, 0.73] | 1.38 | [0.91, 2.08] | 0.742 |
|  | HC : RES | -0.22 | [-0.63, 0.19] | 0.81 | [0.53, 1.21] | 0.747 |
| DMN-RTN | MA : RES | -0.19 | [-0.59, 0.22] | 0.83 | [0.55, 1.25] | 0.767 |
|  | VU : RES | 0.25 | [-0.17, 0.67] | 1.28 | [0.85, 1.95] | 0.742 |
|  | HC : RES | 0.08 | [-0.35, 0.51] | 1.09 | [0.71, 1.67] | 0.925 |
| DMN-SN | MA : RES | -0.16 | [-0.56, 0.24] | 0.85 | [0.57, 1.27] | 0.772 |
|  | VU : RES | 0.05 | [-0.35, 0.45] | 1.05 | [0.7, 1.57] | 0.955 |
|  | HC : RES | 0.25 | [-0.15, 0.65] | 1.28 | [0.86, 1.92] | 0.742 |
| DMN-SMN(H) | MA : RES | 0.05 | [-0.36, 0.45] | 1.05 | [0.7, 1.58] | 0.955 |
|  | VU : RES | 0.21 | [-0.2, 0.62] | 1.23 | [0.82, 1.86] | 0.747 |
|  | HC : RES | -0.37 | [-0.79, 0.05] | 0.69 | [0.45, 1.05] | 0.742 |
| DMN-SMN(M) | MA : RES | -0.16 | [-0.56, 0.24] | 0.85 | [0.57, 1.28] | 0.772 |
|  | VU : RES | 0.20 | [-0.21, 0.61] | 1.22 | [0.81, 1.85] | 0.747 |
|  | HC : RES | -0.10 | [-0.52, 0.31] | 0.90 | [0.6, 1.36] | 0.887 |
| DMN-VN | MA : RES | -0.29 | [-0.7, 0.12] | 0.75 | [0.5, 1.13] | 0.742 |
|  | VU : RES | -0.16 | [-0.56, 0.25] | 0.85 | [0.57, 1.28] | 0.772 |
|  | HC : RES | 0.01 | [-0.4, 0.42] | 1.01 | [0.67, 1.53] | 1.000 |
| DMN-VAN | MA : RES | -0.12 | [-0.52, 0.27] | 0.88 | [0.59, 1.32] | 0.854 |
|  | VU : RES | 0.06 | [-0.36, 0.48] | 1.06 | [0.7, 1.61] | 0.947 |
|  | HC : RES | 0.11 | [-0.3, 0.51] | 1.11 | [0.74, 1.67] | 0.884 |
| FPN-Amygdala | MA : RES | 0.00 | [-0.4, 0.4] | 1.00 | [0.67, 1.49] | 1.000 |
|  | VU : RES | 0.06 | [-0.35, 0.48] | 1.07 | [0.71, 1.61] | 0.938 |
|  | HC : RES | 0.17 | [-0.22, 0.57] | 1.19 | [0.8, 1.77] | 0.772 |
| FPN-FPN | MA : RES | 0.12 | [-0.28, 0.53] | 1.13 | [0.75, 1.69] | 0.858 |
|  | VU : RES | 0.21 | [-0.19, 0.62] | 1.24 | [0.83, 1.86] | 0.747 |
|  | HC : RES | 0.11 | [-0.3, 0.52] | 1.12 | [0.74, 1.68] | 0.884 |
| FPN-Hippocampus | MA : RES | -0.14 | [-0.54, 0.27] | 0.87 | [0.58, 1.31] | 0.846 |
|  | VU : RES | -0.07 | [-0.48, 0.34] | 0.93 | [0.62, 1.4] | 0.925 |
|  | HC : RES | 0.03 | [-0.38, 0.44] | 1.03 | [0.68, 1.55] | 1.000 |
| FPN-RTN | MA : RES | 0.07 | [-0.33, 0.48] | 1.08 | [0.72, 1.62] | 0.925 |
|  | VU : RES | 0.29 | [-0.12, 0.7] | 1.34 | [0.89, 2.01] | 0.742 |
|  | HC : RES | 0.33 | [-0.08, 0.74] | 1.39 | [0.92, 2.1] | 0.742 |
| FPN-SN | MA : RES | -0.01 | [-0.41, 0.4] | 0.99 | [0.66, 1.49] | 1.000 |
|  | VU : RES | 0.23 | [-0.18, 0.63] | 1.25 | [0.83, 1.88] | 0.742 |
|  | HC : RES | 0.16 | [-0.25, 0.57] | 1.17 | [0.78, 1.76] | 0.772 |
| FPN-SMN(H) | MA : RES | 0.43 | [0.02, 0.84] | 1.53 | [1.02, 2.31] | 0.742 |
|  | VU : RES | 0.39 | [-0.02, 0.8] | 1.47 | [0.98, 2.23] | 0.742 |
|  | HC : RES | 0.17 | [-0.24, 0.57] | 1.18 | [0.79, 1.77] | 0.772 |
| FPN-SMN(M) | MA : RES | 0.22 | [-0.18, 0.62] | 1.25 | [0.84, 1.87] | 0.742 |
|  | VU : RES | 0.25 | [-0.15, 0.66] | 1.29 | [0.86, 1.94] | 0.742 |
|  | HC : RES | -0.19 | [-0.59, 0.22] | 0.83 | [0.55, 1.25] | 0.767 |
| FPN-VN | MA : RES | 0.16 | [-0.25, 0.57] | 1.17 | [0.78, 1.77] | 0.772 |
|  | VU : RES | 0.30 | [-0.1, 0.71] | 1.36 | [0.9, 2.04] | 0.742 |
|  | HC : RES | 0.14 | [-0.26, 0.55] | 1.15 | [0.77, 1.73] | 0.827 |
| FPN-VAN | MA : RES | 0.02 | [-0.11, 0.14] | 1.02 | [0.9, 1.15] | 0.940 |
|  | VU : RES | 0.01 | [-0.12, 0.14] | 1.01 | [0.89, 1.15] | 0.963 |
|  | HC : RES | -0.05 | [-0.18, 0.08] | 0.95 | [0.84, 1.09] | 0.858 |
| RTN-Amygdala | MA : RES | 0.14 | [0.01, 0.27] | 1.15 | [1.01, 1.31] | 0.263 |
|  | VU : RES | 0.03 | [-0.1, 0.16] | 1.03 | [0.9, 1.17] | 0.940 |
|  | HC : RES | 0.16 | [0.03, 0.29] | 1.17 | [1.03, 1.33] | 0.263 |
| RTN-Hippocampus | MA : RES | -0.11 | [-0.24, 0.02] | 0.90 | [0.79, 1.02] | 0.432 |
|  | VU : RES | -0.02 | [-0.15, 0.11] | 0.98 | [0.86, 1.12] | 0.940 |
|  | HC : RES | -0.07 | [-0.2, 0.06] | 0.93 | [0.82, 1.06] | 0.694 |
| RTN-RTN | MA : RES | 0.02 | [-0.11, 0.15] | 1.02 | [0.9, 1.16] | 0.940 |
|  | VU : RES | 0.06 | [-0.07, 0.19] | 1.06 | [0.93, 1.21] | 0.765 |
|  | HC : RES | -0.18 | [-0.31, -0.05] | 0.83 | [0.73, 0.95] | 0.263 |
| RTN-SN | MA : RES | 0.02 | [-0.11, 0.15] | 1.02 | [0.9, 1.16] | 0.940 |
|  | VU : RES | 0.04 | [-0.09, 0.17] | 1.04 | [0.92, 1.19] | 0.871 |
|  | HC : RES | 0.12 | [-0.01, 0.25] | 1.13 | [0.99, 1.28] | 0.369 |
| RTN-SMN(H) | MA : RES | 0.14 | [0.01, 0.27] | 1.15 | [1.01, 1.31] | 0.263 |
|  | VU : RES | -0.11 | [-0.24, 0.02] | 0.90 | [0.79, 1.02] | 0.432 |
|  | HC : RES | -0.04 | [-0.16, 0.09] | 0.96 | [0.85, 1.1] | 0.940 |
| RTN-SMN(M) | MA : RES | 0.01 | [-0.12, 0.14] | 1.01 | [0.89, 1.15] | 0.940 |
|  | VU : RES | 0.03 | [-0.1, 0.16] | 1.03 | [0.91, 1.17] | 0.940 |
|  | HC : RES | 0.01 | [-0.12, 0.14] | 1.01 | [0.89, 1.15] | 0.940 |
| RTN-VN | MA : RES | -0.15 | [-0.28, -0.02] | 0.86 | [0.76, 0.98] | 0.263 |
|  | VU : RES | -0.04 | [-0.17, 0.09] | 0.96 | [0.84, 1.09] | 0.871 |
|  | HC : RES | -0.07 | [-0.19, 0.06] | 0.93 | [0.82, 1.06] | 0.694 |
| RTN-VAN | MA : RES | 0.06 | [-0.07, 0.18] | 1.06 | [0.93, 1.2] | 0.782 |
|  | VU : RES | 0.05 | [-0.07, 0.18] | 1.06 | [0.93, 1.2] | 0.786 |
|  | HC : RES | 0.08 | [-0.05, 0.21] | 1.08 | [0.95, 1.23] | 0.654 |
| SN-Amygdala | MA : RES | -0.04 | [-0.17, 0.09] | 0.96 | [0.84, 1.09] | 0.871 |
|  | VU : RES | 0.11 | [-0.02, 0.24] | 1.12 | [0.98, 1.27] | 0.432 |
|  | HC : RES | -0.03 | [-0.16, 0.1] | 0.97 | [0.86, 1.11] | 0.940 |
| SN-Hippocampus | MA : RES | -0.06 | [-0.19, 0.07] | 0.94 | [0.83, 1.07] | 0.756 |
|  | VU : RES | 0.02 | [-0.11, 0.15] | 1.02 | [0.9, 1.16] | 0.940 |
|  | HC : RES | -0.03 | [-0.16, 0.1] | 0.97 | [0.85, 1.1] | 0.940 |
| SN-SN | MA : RES | -0.02 | [-0.15, 0.11] | 0.98 | [0.86, 1.12] | 0.940 |
|  | VU : RES | -0.02 | [-0.15, 0.11] | 0.98 | [0.86, 1.11] | 0.940 |
|  | HC : RES | 0.07 | [-0.06, 0.2] | 1.07 | [0.94, 1.22] | 0.694 |
| SN-SMN(H) | MA : RES | -0.01 | [-0.14, 0.12] | 0.99 | [0.87, 1.12] | 0.940 |
|  | VU : RES | 0.14 | [0.01, 0.27] | 1.15 | [1.01, 1.3] | 0.295 |
|  | HC : RES | 0.03 | [-0.1, 0.16] | 1.03 | [0.91, 1.17] | 0.940 |
| SN-SMN(M) | MA : RES | 0.02 | [-0.11, 0.15] | 1.02 | [0.9, 1.16] | 0.940 |
|  | VU : RES | 0.04 | [-0.09, 0.17] | 1.04 | [0.92, 1.18] | 0.890 |
|  | HC : RES | -0.05 | [-0.18, 0.08] | 0.95 | [0.84, 1.08] | 0.830 |
| SN-VN | MA : RES | 0.00 | [-0.13, 0.13] | 1.00 | [0.88, 1.13] | 0.981 |
|  | VU : RES | -0.08 | [-0.21, 0.05] | 0.92 | [0.81, 1.05] | 0.654 |
|  | HC : RES | -0.03 | [-0.16, 0.1] | 0.97 | [0.85, 1.1] | 0.940 |
| SN-VAN | MA : RES | 0.12 | [-0.01, 0.25] | 1.13 | [0.99, 1.28] | 0.398 |
|  | VU : RES | -0.06 | [-0.19, 0.07] | 0.94 | [0.83, 1.07] | 0.782 |
|  | HC : RES | 0.02 | [-0.11, 0.15] | 1.02 | [0.9, 1.16] | 0.940 |
| SMN(H)-Amygdala | MA : RES | 0.00 | [-0.13, 0.13] | 1.00 | [0.88, 1.14] | 0.969 |
|  | VU : RES | 0.05 | [-0.08, 0.17] | 1.05 | [0.92, 1.19] | 0.862 |
|  | HC : RES | -0.15 | [-0.28, -0.02] | 0.86 | [0.76, 0.98] | 0.263 |
| SMN(H)-Hippocampus | MA : RES | 0.10 | [-0.03, 0.23] | 1.11 | [0.97, 1.26] | 0.459 |
|  | VU : RES | 0.03 | [-0.1, 0.16] | 1.03 | [0.9, 1.17] | 0.940 |
|  | HC : RES | 0.16 | [0.03, 0.29] | 1.17 | [1.03, 1.33] | 0.263 |
| SMN(H)-SMN(H) | MA : RES | 0.17 | [0.04, 0.3] | 1.19 | [1.04, 1.35] | 0.263 |
|  | VU : RES | 0.01 | [-0.12, 0.14] | 1.01 | [0.89, 1.15] | 0.940 |
|  | HC : RES | 0.01 | [-0.11, 0.14] | 1.01 | [0.89, 1.15] | 0.940 |
| SMN(H)-SMN(M) | MA : RES | -0.07 | [-0.19, 0.06] | 0.94 | [0.82, 1.06] | 0.694 |
|  | VU : RES | 0.07 | [-0.06, 0.2] | 1.07 | [0.94, 1.22] | 0.694 |
|  | HC : RES | -0.15 | [-0.28, -0.02] | 0.86 | [0.76, 0.98] | 0.263 |
| SMN(H)-VN | MA : RES | -0.22 | [-0.35, -0.09] | 0.80 | [0.7, 0.91] | 0.086 |
|  | VU : RES | 0.01 | [-0.12, 0.14] | 1.01 | [0.89, 1.16] | 0.940 |
|  | HC : RES | 0.02 | [-0.11, 0.15] | 1.02 | [0.89, 1.16] | 0.940 |
| SMN(H)-VAN | MA : RES | -0.03 | [-0.16, 0.1] | 0.97 | [0.86, 1.11] | 0.940 |
|  | VU : RES | 0.07 | [-0.06, 0.2] | 1.07 | [0.95, 1.22] | 0.694 |
|  | HC : RES | 0.08 | [-0.05, 0.21] | 1.08 | [0.95, 1.23] | 0.654 |
| SMN(M)-Amygdala | MA : RES | -0.09 | [-0.22, 0.04] | 0.92 | [0.81, 1.04] | 0.544 |
|  | VU : RES | 0.06 | [-0.07, 0.18] | 1.06 | [0.93, 1.2] | 0.782 |
|  | HC : RES | -0.16 | [-0.29, -0.03] | 0.85 | [0.75, 0.97] | 0.263 |
| SMN(M)-Hippocampus | MA : RES | -0.10 | [-0.22, 0.03] | 0.91 | [0.8, 1.03] | 0.511 |
|  | VU : RES | 0.11 | [-0.02, 0.24] | 1.11 | [0.98, 1.27] | 0.432 |
|  | HC : RES | -0.14 | [-0.27, -0.01] | 0.87 | [0.76, 0.99] | 0.263 |
| SMN(M)-SMN(M) | MA : RES | 0.01 | [-0.12, 0.14] | 1.01 | [0.89, 1.15] | 0.963 |
|  | VU : RES | 0.11 | [-0.02, 0.23] | 1.11 | [0.98, 1.26] | 0.432 |
|  | HC : RES | -0.15 | [-0.28, -0.02] | 0.86 | [0.76, 0.98] | 0.263 |
| SMN(M)-VN | MA : RES | -0.01 | [-0.13, 0.12] | 0.99 | [0.87, 1.13] | 0.969 |
|  | VU : RES | 0.00 | [-0.13, 0.13] | 1.00 | [0.88, 1.14] | 0.981 |
|  | HC : RES | 0.05 | [-0.07, 0.18] | 1.06 | [0.93, 1.2] | 0.786 |
| SMN(M)-VAN | MA : RES | 0.12 | [0, 0.25] | 1.13 | [1, 1.29] | 0.360 |
|  | VU : RES | -0.10 | [-0.23, 0.03] | 0.91 | [0.8, 1.03] | 0.502 |
|  | HC : RES | 0.10 | [-0.03, 0.23] | 1.11 | [0.97, 1.26] | 0.459 |
| VN-Amygdala | MA : RES | 0.02 | [-0.11, 0.15] | 1.02 | [0.89, 1.16] | 0.940 |
|  | VU : RES | -0.03 | [-0.16, 0.1] | 0.97 | [0.85, 1.1] | 0.940 |
|  | HC : RES | 0.07 | [-0.06, 0.19] | 1.07 | [0.94, 1.21] | 0.694 |
| VN-Hippocampus | MA : RES | 0.03 | [-0.1, 0.15] | 1.03 | [0.9, 1.17] | 0.940 |
|  | VU : RES | -0.01 | [-0.14, 0.12] | 0.99 | [0.87, 1.13] | 0.969 |
|  | HC : RES | 0.05 | [-0.08, 0.18] | 1.05 | [0.92, 1.19] | 0.858 |
| VN-VN | MA : RES | -0.13 | [-0.26, 0] | 0.88 | [0.77, 1] | 0.342 |
|  | VU : RES | 0.08 | [-0.05, 0.21] | 1.08 | [0.95, 1.23] | 0.654 |
|  | HC : RES | 0.11 | [-0.02, 0.24] | 1.11 | [0.98, 1.27] | 0.432 |
| VN-VAN | MA : RES | -0.07 | [-0.2, 0.06] | 0.93 | [0.82, 1.06] | 0.694 |
|  | VU : RES | 0.10 | [-0.03, 0.22] | 1.10 | [0.97, 1.25] | 0.516 |
|  | HC : RES | -0.01 | [-0.13, 0.12] | 0.99 | [0.88, 1.13] | 0.969 |
| VAN-Amygdala | MA : RES | -0.09 | [-0.22, 0.04] | 0.91 | [0.8, 1.04] | 0.543 |
|  | VU : RES | 0.00 | [-0.13, 0.13] | 1.00 | [0.87, 1.13] | 0.969 |
|  | HC : RES | 0.09 | [-0.04, 0.22] | 1.10 | [0.96, 1.25] | 0.536 |
| VAN-Hippocampus | MA : RES | 0.09 | [-0.04, 0.22] | 1.09 | [0.96, 1.24] | 0.543 |
|  | VU : RES | 0.04 | [-0.09, 0.17] | 1.04 | [0.91, 1.18] | 0.940 |
|  | HC : RES | -0.07 | [-0.2, 0.06] | 0.93 | [0.82, 1.06] | 0.694 |
| VAN-VAN | MA : RES | 0.01 | [-0.12, 0.14] | 1.01 | [0.89, 1.15] | 0.940 |
|  | VU : RES | -0.01 | [-0.14, 0.11] | 0.99 | [0.87, 1.12] | 0.940 |
|  | HC : RES | 0.02 | [-0.1, 0.15] | 1.02 | [0.9, 1.16] | 0.940 |

*Note*. Abbreviations explained: auditory network (AN), Cingulo-parietal network (CPN), Cingulo-opecular network (CON), dorsal attention network (DAN), default mode network (DMN), frontal-parietal network (FPN), retrosplenial temporal network (RTN), salience network (SN), sensorimotor hand network (SMN[H]), sensorimotor mouth network (SMN[M]), visual network (VN), ventral attention network (VAN)
